# An in‐depth benchmark framework for evaluating single cell RNA‐seq dropout imputation methods and the development of an improved algorithm afMF

**DOI:** 10.1002/ctm2.70283

**Published:** 2025-03-22

**Authors:** Jinghan Huang, Anson C. M. Chow, Nelson L. S. Tang, Sheung Chi Phillip Yam

**Affiliations:** ^1^ Department of Chemical Pathology Faculty of Medicine The Chinese University of Hong Kong Hong Kong Hong Kong SAR China; ^2^ Department of Statistics Faculty of Science The Chinese University of Hong Kong Hong Kong Hong Kong SAR China; ^3^ Cytomics Limited, Hong Kong Science Park Hong Kong Hong Kong SAR China; ^4^ Li Ka Shing Institute of Health Sciences and CAS Center for Excellence in Animal Evolution and Genetics Faculty of Medicine The Chinese University of Hong Kong Hong Kong Hong Kong SAR China

1

Dear editor,

The presence of the inflated zeros in single cell RNA‐seq still represents a challenge. Imputation of zeros can be performed but it is not commonly used in real applications because of their uncertain benefits and the lack of in‐depth benchmark for various downstream analyses. Here, we performed two tasks: an in‐depth benchmark framework was developed to compare imputation algorithms; second, an improved algorithm, afMF, was developed. Our results indicated that matrix‐theory‐based algorithms such as afMF had great and stable performance across various applications and generally outperformed raw log‐normalization and others. In contrast, complicated methods were prone to overfitting and data distortion.

Imputation has raised some discussions^1^, ^2^: downstream analyses could benefit from it,^3–^ ^5^ while false‐positives may be introduced and zeros may contain important information too.^6^ No definitive conclusion has been reached so far. Imputation algorithms have been developed for years. Meanwhile, several comparative studies for dropout imputation have been conducted^1^, ^7^, ^8^, ^9^ but had several obvious issues: (1) lack of in‐depth analysis, for example, automatic cell type annotation, pseudobulk DE analysis, GSEA, cell–cell communication, AUCell and SCENIC, integration with spatial transcriptomics, etc.; (2) limited number of datasets, dataset types and tested algorithms, that is, only less than 5 or 6 datasets were used and the evaluated algorithms were developed a few years ago; (3) using biased, unreasonable performance metrics or confined to basic summary statistics only; (4) confined to using many simulated datasets (which have been shown to be much simpler and cannot reflect the complexity of real data). These limitations are also complicated by lack of real datasets with given ground truth. At the moment, most of the imputation algorithms are not used in any real‐world applications or only confined to be used in a limited number of downstream applications (e.g., cell type clustering). A more thorough benchmark of the compatibility between imputation and key downstream applications is required.

Here, we evaluated the compatibility between prior imputation algorithms and various downstream tasks. This issue is obvious when applying downstream algorithms that have in‐situ imputation steps or are designed for sparse data, as prior imputation may be unnecessary or worsen the results. Some researchers used zero‐inflated models instead of imputation but such methods may not perfectly fit for scRNA‐seq.^10^

Motivated by the benchmark review,^2^ we developed an improved benchmark framework to address these issues by including previously well‐established metrics and various novel features (Figure 1). These novel advantages includes: (1) using more than 25 real (mixture/purified cell type/time‐course) or simulated datasets (Additional file 1 Table S1), which is much more than other benchmark studies; (2) including 21 top or new algorithms with acceptable scalability (Table S2), which is the most across various benchmark studies; (3) a pre‐screening test to select algorithms for further evaluations; (4) visualizations (Gene Expression Violin plots; PCA/UMAP plots; Cell–Cell Correlations); (5) Differential Expression (DE) Analysis using Pseudobulk DE analysis; (6) Enrichment Analysis (GSEA); (7) Automatic Cell Type Annotation: SCINA and scType; (8) Pseudotime Trajectory Analysis using popular Monocle3, Slingshot and DPT; (9) AUCell and SCENIC regulatory analysis; (10) Cell–Cell Communication: CellPhoneDB and CellChat; (11) Integration of spatial transcriptomics with scRNA‐seq (Seurat); (12) an improved imputation algorithm ‘afMF’ (**a**daptive **f**ull **M**atrix **F**actorization) (Method S1). These features had made our study exhaustive, unique, and novel.

**FIGURE 1:**
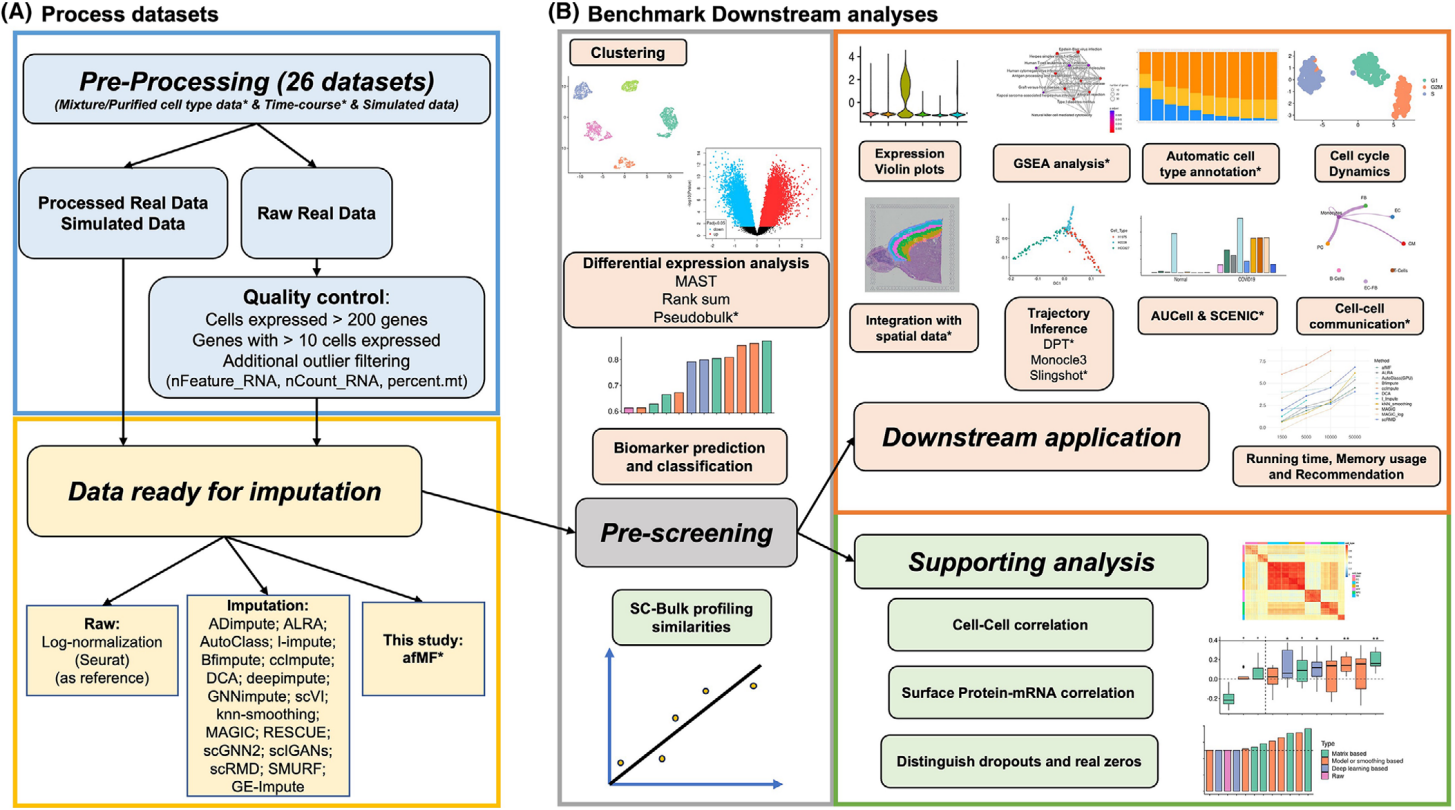
Workflow in this study. Twenty‐six datasets including bulk, mixture/purified cell type, time‐course and simulated mixture data were pre‐processed with quality control, normalization, and imputation. Performances of raw and various imputation methods were evaluated by eleven downstream applications and four types of supporting analyses. A pre‐screening test, including differential expression analysis, biomarker prediction and classification, clustering and single cell‐bulk profiling similarities on two datasets GSE75748 and GSE81861, was performed to initially select the imputation algorithms for in‐depth evaluations. ‘*’ represents novel features in the new developed benchmark framework.

afMF is an improved matrix‐theory‐based algorithm that builds upon another algorithm ‘ALRA’. afMF is different from ALRA in that an iterative process is used to optimize two low‐rank matrices which may account for the added benefits shown in these evaluations. While ALRA employs randomized SVD, afMF applies a different way by utilizing full matrix factorization. Details of the algorithm could be found in Method S1 afMF.

To reduce the variabilities from other factors such as preprocessing, datasets, integrity of annotation that may influence the true impact of imputation, we (1) transformed the data to make all processed/imputed data in log space so that they are more comparable; (2) applied as many high‐quality and various datasets as we can to reduce dataset bias; (3) used datasets with matched bulk or ‘gold standard’ annotations, for example, with wet lab experiment validations or CITE‐seq with surface protein markers, or cell‐cycle/time experiment with well‐known checkpoint description. We compared various imputations with the well‐established Seurat log‐normalization where the data is also in log space. More introductions regarding dropout imputation are placed in Additional file 1 Note S1.

Based on our pre‐screening results (Figure S1, Method S1), ten algorithms with stable performance (i.e., generally not worse than no‐imputation across all pre‐screening evaluations) were selected for further evaluations (Table S2), as performance largely worse than that no‐imputation in any aspect may indicate strong introduction of unwanted patterns and data distortion.

The impact of imputation on the basic data analysis and visualizations was first explored (Additional file 2 Method S2 and Figures S2 to S5) and discussed in Note S2. Combining these results, only afMF, ALRA and scRMD did not fabricate artefactual structure in PCA and provided better visualizations in gene expression violin plots, 2‐D PCA, and cell–cell correlations.

Differential expression (DE) between conditions was analyzed by three methods (Additional file 3 Method S3). Using MAST/rank sum test, higher *p*‐value‐based‐rank concordance between bulk and afMF‐imputed DE results were observed for all data types (Figure 2A; Figures S6 and S7). These conclusions held when limiting the genes to the top 1000 bulk DEGs (Figure S8). The top 500 DEGs showed greater statistical significance in afMF and other three algorithms (Figure 2A; Figures S6 and S7). Only afMF and I_Impute showed generally lower false positive rates in all types of data (Figure 2A and Figure S9). Higher logFC Spearman correlations were observed between bulk and afMF‐imputed results (Figure S10). However, imputation is incompatible with pseudobulk analysis using limma‐trend (Figure S11), which suggested that pseudobulk may work as smoothing and thus heavily decreased the dropout influence in DE analysis. Generally, enhancement for DE was only found in MAST/rank sum test using afMF, but not in pseudobulk. More descriptions and interpretations are placed in Note S3.

**FIGURE 2:**
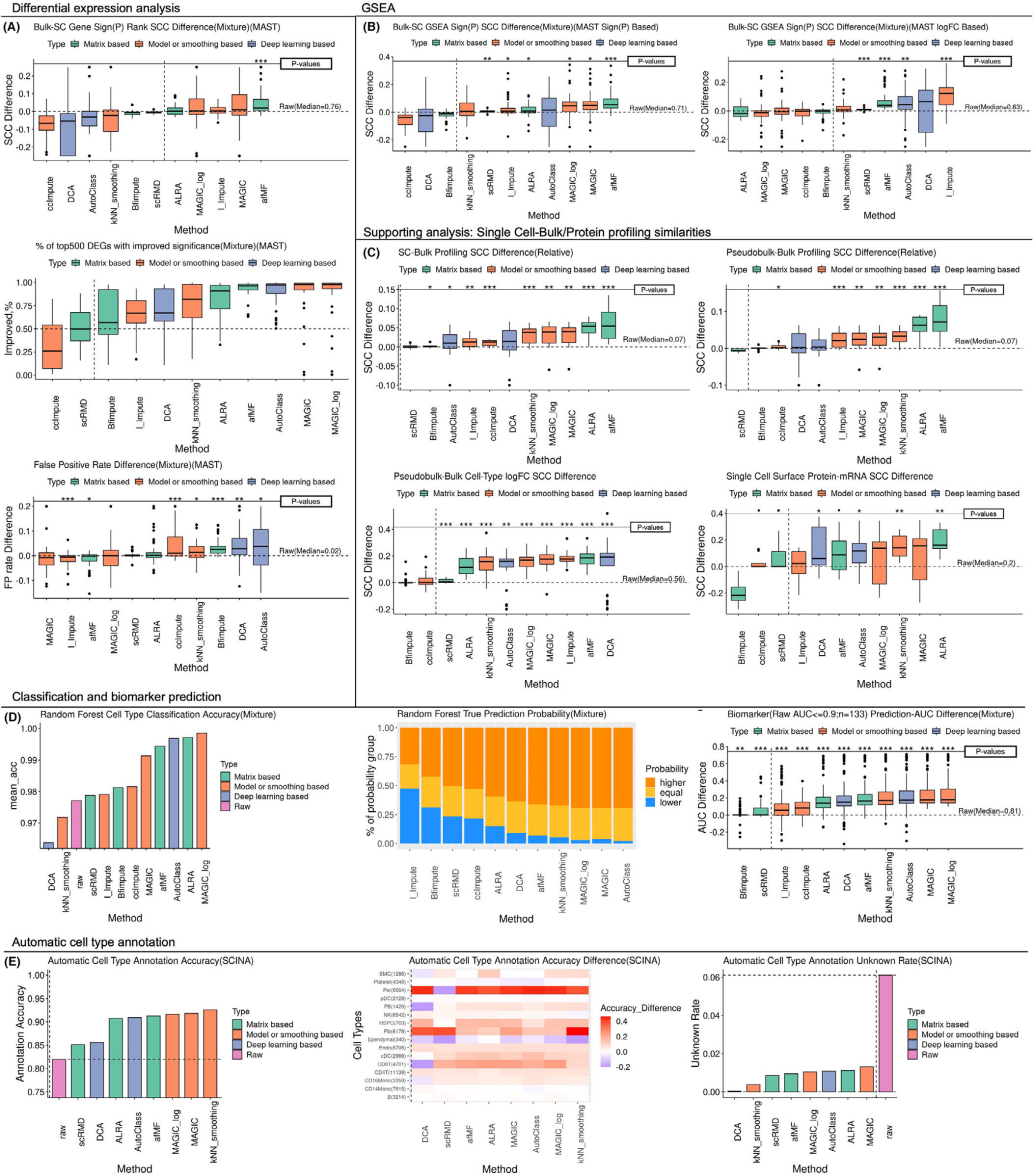
Performance of imputations in differential expression analysis (MAST), GSEA, SC‐bulk/protein profiling similarity, classification, biomarker prediction and automatic cell type annotation. Imputations on DE analysis were evaluated with matched bulk results as ‘gold standard’. The gene expression between pairwise cell types were compared. DE analysis for bulk and scRNA‐seq were performed by limma‐voom and MAST respectively. Results were ranked by sign ‐log10(*p*‐value) and the absolute values of fold changes. The GSEA was performed for each of the DE results based on either sign ‐log10(*p*‐value) or logFC. The gene ontology (GO) terms were used. The bulk GSEA results were used as the gold standard. The Spearman correlation coefficients (SCC) were calculated for term ‘sign (Enrichment Score) × ‐log10(*p*‐value)’ between the bulk GSEA results and the single cell GSEA results. Supporting analysis included SC‐Bulk Profiling Similarity and mRNA‐Surface Protein Correlation. Classifications for different cell types were performed using Random Forest (RF). The top 10 DEGs for each comparison in bulk data were used as input features. For each dataset, 70% samples were used as training set and 30% were for testing. The biomarker predictive performance of different methods was evaluated. Top 10 DEGs between pairwise cell types in bulk data were used as biomarkers. SCINA was applied to evaluate imputations on automatic cell type annotation. Two well‐labelled data (GSE155673 and ROSMAP brain) were used. The cell‐type‐specific genes used as input were collected from Azimuth and PanglaoDB. In these evaluations, some metrics were subtracted by the values of the raw results. Some extreme values have been limited to a cutoff for better visualization. One, two and three ‘*’ represents ‘*p* < 0.05′, ‘*p* < 0.01′ and ‘P < 0.005′ respectively and ‘.’ represents ‘P < 0.1′, compared to raw log‐normalization using Wilcoxon rank sum test. (A) DE: The SCC of the gene ranks between the bulk and the single cell results, the significances of the top 500 DEGs (in bulk) and the false positive rates (the third tertile genes in bulk results presented in the top 500 DEGs in single cell results) were compared; (B) GSEA; (C) Supporting analysis: relative SCC between each single cell/pseudobulk profile and the bulk profile (within same group), SCCs of logFC between different groups between pseudobulk profiles and bulk profiles, SCCs between six marker gene mRNAs in two tissues and corresponding surface protein measurement were compared. (D) Classification and Biomarker Prediction: The classification accuracy and RF prediction probability for true label were calculated. The predictive performance was evaluated by calculating the AUC for each of the biomarkers in each comparison. (E) The annotation accuracy and unknown rate were compared.

GSEA aims to study the enrichments of DEGs in specific biological pathways using results from DE analysis (Method S3). Using either MAST/rank sum sign ‐log_10_P or logFC as input, higher Spearman correlations between bulk and afMF‐imputed GSEA were observed for all data types (Figure 2B; Figures S12 to S14). These conclusions held when limiting to enrichment terms with bulk *p* < 0.05 (Figure S13). Regarding pseudobulk DE results, afMF and other four algorithms increased the correlations only when using logFC as input (Figure S15). Generally, afMF presented the most stable improvements. More descriptions are placed in Note S3.

Additional support for imputation can be gathered from cell sorting datasets or protein assay which may better reflect the ground‐truth (Additional file 4 Method S4). Using datasets with matched bulk data, higher relative and absolute Spearman correlations between same‐cell‐type single cell/pseudobulk and bulk profiling, and correlations of the pairwise‐cell‐type logFC between pseudobulk and bulk, were observed for afMF‐imputed data and most other imputed data (Figure 2C and Figure S16). Using a CITE‐seq dataset with mRNA and surface‐protein measurement, higher Spearman correlations between selected mRNA and surface‐protein were observed in ALRA, afMF, etc. (Figure 2C and Figure S16). More descriptions are placed in Note S4.

Classification (Additional file 5 Method S5) is useful when predicting unknown labels. Using Random Forest model, we observed higher classification accuracy and correct‐cell‐type prediction probabilities in afMF and nearly all the other algorithms for all data types (Figure 2D; Figures S17 and S18). Marker genes can be used to identify different cell types (Method S5). Higher AUCs for marker genes to discriminate cell status were discovered for afMF and other five algorithms (Figure 2D; Figures S17 and S18). Only afMF, kNN‐smoothing and ALRA enhanced the detection while controlling false‐positive rate (Figure S18). Interestingly, the use of imputation for cell type annotation^11^ may be underestimated (Method S5). Using two automatic cell type annotation tools SCINA and ScType, higher annotation accuracy, F1 scores and true prediction probabilities were observed for afMF and all other algorithms (Figure 2E and Figure S19) except for DCA. Notably, all of them resulted in lower unknown annotation rates. More descriptions are placed in Note S5.

Clustering is the essential step for exploring subtypes (Additional file 6 Method S6). Using Louvain and K‐means algorithms with four clustering metrics, afMF and MAGIC‐log showed improvements across all metrics compared to no‐imputation (Figure 3A). In UMAP projection, afMF/ALRA/scRMD remained consistent structure of clusters, while others (e.g., kNN_smoothing) generated unexpected patterns (Figures S20 and S21). Cell‐cycle dynamics have been well‐studied (Method S6). Using a cell‐cycle dataset with ground‐truth, the prediction accuracy and statistical significance of the comparisons of predicted cell‐cycle‐scores between different known cell‐cycles were improved when using afMF/ALRA, etc. (Figure 3B and Figure S22). More distinct separations between different cell‐cycles were also observed using afMF and ALRA in 2‐D UMAP (Figure 3C). More descriptions are placed in Note S6.

**FIGURE 3:**
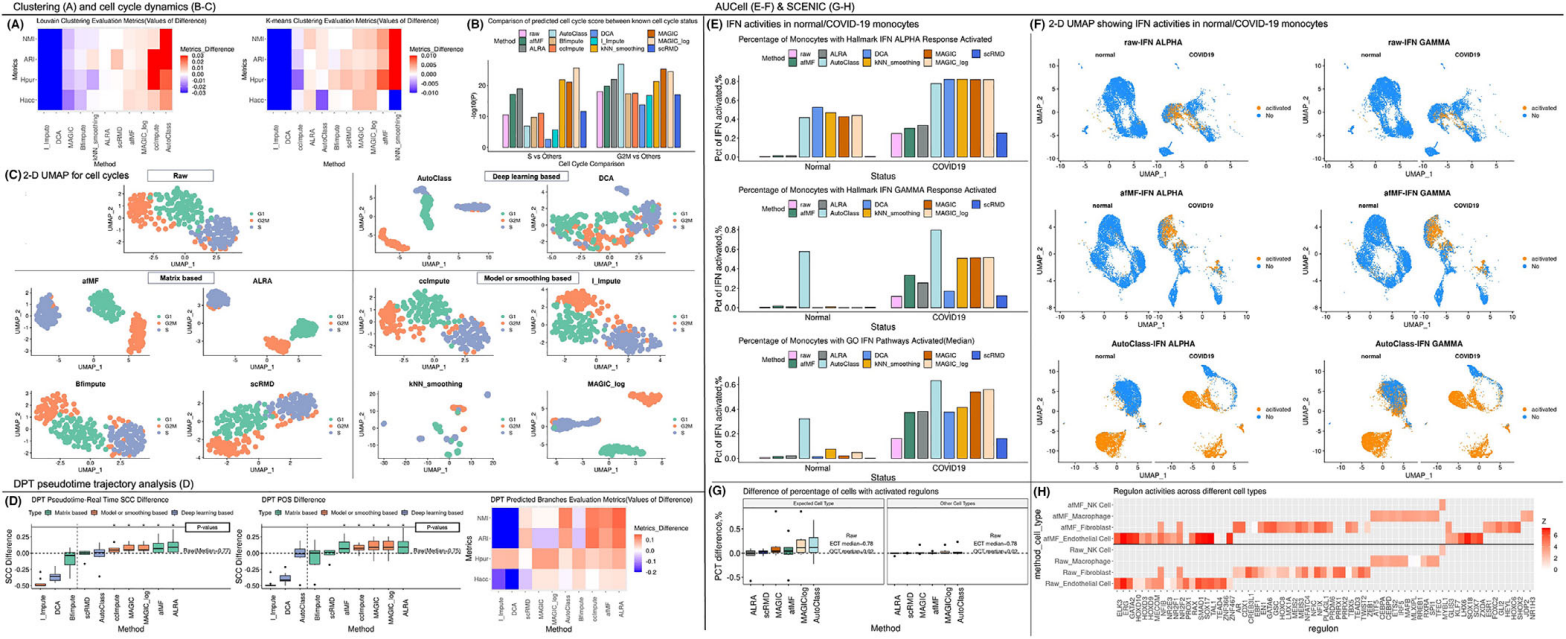
Performance of imputations in dimension reduction, clustering, cell cycle dynamics, pseudotime trajectory analysis and AUCell and SCENIC. Entropy of accuracy ($H_{acc}$), Entropy of purity ($H_{pur}$), Adjusted rand index (ARI) and normalized mutual information (NMI) were used to evaluate the clustering performance with Louvain clustering and K‐means algorithms. Three labelled (i.e., cell type) datasets were used. The number of clusters was set to the number of known cell type labels in each dataset. Top 3000 high variable genes were picked out followed by running PCA. The top 10 PCs were used for clustering. ARI and NMI were subtracted by the results of the raw data (vice versa for $H_{acc}$ and $H_{pur}$ for better visualization) and the medians were taken. In cell cycle dynamics, cell cycle status and score of each cell was predicted by the Seurat algorithm. The performances of imputations on pseudotime trajectory analysis were evaluated with DPT using data with real time and lineage labels. The starting point and end point were set as ‘tips’ based on known information with other parameters set to default. The compatibility between AUCell and different imputations were evaluated, where the activations of the IFN pathways between COVID‐19 and healthy controls were compared among monocytes. The IFN‐related pathways were collected from Hallmark and GO. The compatibility between SCENIC and different imputations were evaluated. In the above evaluations, some metrics were subtracted by the values of the raw results. Extreme values have been set to a cutoff for better visualization. One, two and three ‘*’ represents ‘*p* < 0.05′, ‘*p* < 0.01′ and ‘*p* < 0.005′ respectively and ‘.’ represents ‘*p* < 0.1′, compared to raw log‐normalization using Wilcoxon rank sum test. (A) Clustering: median values of the four metrics in Louvain and K‐means clustering. (B–C) Cell cycle dynamics: rank sum tests were performed on the predicted cell cycle scores between different known cell cycles and the *p*‐values were compared; UMAP plots for ground truth cell cycles. (D) Pseudotime trajectory analysis: SCC and Pseudo‐temporal ordering score (POS) were calculated between the predicted DPT values and the real time labels; the predicted branches by DPT were further evaluated with the four‐clustering metrics and the true branches were used as ground truth. (E–F) AUCell: each cell was assigned a binary status (activated or not) for each pathway by the AUCell pipeline and the percentages of monocytes with activated IFN pathway were compared between COVID‐19 and healthy controls. (G–H) SCENIC: seven well‐established regulons were used to determine the activation status in a selected cell. A cell‐type‐specific regulon was expected to be activated only in the specific cell type but not the other cell types. The percentages of cells that with activated regulons within expected cell type (ECT) and other cell types (OCT) were calculated and compared; the resulted regulons with Z‐score ≥ 3 for raw and afMF were collected and compared.

Trajectory inference enables the study of cell differentiation and development (Additional file 7 Method S7). When using DPT trajectory analysis, afMF and the other three algorithms improved the pseudotime analysis (i.e., correlations between the known time and predicted pseudotime and pseudo‐temporal score) and branch predictions (Figure 3D). In diffusion map, afMF showed better continuum trajectories while others showed less improvements or distorted the patterns (Figure S23). For Monocle3 trajectory analysis, only MAGIC improved the analysis. While afMF and AutoClass showed slight improvements, other algorithms had no or negative influence. Slingshot trajectory analysis was incompatible with most imputation algorithms (Figures S24 and S25). More descriptions are placed in Note S7.

AUCell aims to investigate the activities of pathways (e.g., well‐studied interferon (IFN)) in each cell (Additional file 8 Method S8). In afMF and ALRA‐imputed data, increased percentages of monocytes with IFN response activated were only observed within COVID‐19 subjects but not within healthy controls as expected (Figure 3E,F). In contrast, other algorithms showed false positives in controls. SCENIC incorporates AUCell for exploring gene regulatory networks. Seven well‐established cell‐type‐specific ‘regulons’ were selected (Method S8). afMF and MAGIC performed well as they increased the percentages of cells with activated regulons within expected‐cell‐types while remained consistent levels as no‐imputation within unrelated‐cell‐types (Figure 3G). The comparison of all the identified regulons (Z‐score > 3) across all the cell types revealed that most selected algorithms could recover the raw patterns but also added some unique significant regulons (Figure 3H and Figure S26). Of note, these newly generated significant regulons should be further validated through other experiments. More descriptions are placed in Note S8.

CellPhoneDB and CellChat are tools to study cell–cell communications (Method S8). Our results revealed abnormally huge increments of significant interactions after imputations in both CellPhoneDB and CellChat analysis (Figures S27 to S30). Though no ground truth is available for demonstration, they were believed to be the false positives as the patterns are abnormal and many of the interactions are unique. More descriptions are placed in Note S8.

Integrating with scRNA‐seq data is an important step to study spatial transcriptomics. Using Seurat integration pipeline, we observed a clear recovery of the known spatial localization patterns of both neuronal and non‐neuronal subsets with raw SC‐Transform data and MAGIC‐imputed data as reference (Figure S31). In contrast, ALRA/AutoClass/afMF either led to much weaker pattern (e.g., L4 and L5 PT/IT regions) or raised errors due to the incompatibility between algorithms. More descriptions are placed in Note S8.

Real scRNA‐seq data are complicated and difficult to simulate but simulated datasets have the advantage of having ground‐truth. Using simulated datasets generated from Splatter/SplatPop (Additional file 9 Method S9), we found afMF and ALRA performed generally better in most of the analyses (Figures S32 to S35). More descriptions are placed in Note S9.

Good algorithms should have acceptable running‐time and memory‐usage (Additional file 10 Method S10), and most selected algorithms meet the requirements except for I‐Impute (running‐time) (Figure 4A) and ccImpute and Bfimpute (memory‐usage on large datasets) (Figure 4B). Performances of algorithms were rated by comparing with no‐imputation. Generally, matrix‐theory‐based methods such as afMF and ALRA improved the various task performances steadily, while others showed less or no improvements or were incompatible with some downstream tools (Figure 4C,D; Tables S3 and S4). Specifically, afMF ranked among the top algorithms in various evaluations, for example, DE analysis, GSEA, classification, biomarker prediction, automatic cell type annotation, clustering, DPT trajectory analysis, AUCell and SCENIC, SC‐bulk profiling similarity and mRNA‐surface protein correlation. Within the top matrix‐theory algorithms, afMF outperformed ALRA in multiple evaluations (cell‐level DE analysis, GSEA, classification, biomarker prediction, clustering and SC‐bulk profiling similarity) (Table S4). Besides, MAGIC (smoothing) and AutoClass (deep‐learning) also showed some enhanced output in selected applications but produced false positives in other applications. We also found that most imputations are not compatible with certain downstream algorithms, for example, cell–cell communication and pseudobulk‐limma DE analysis.

**FIGURE 4:**
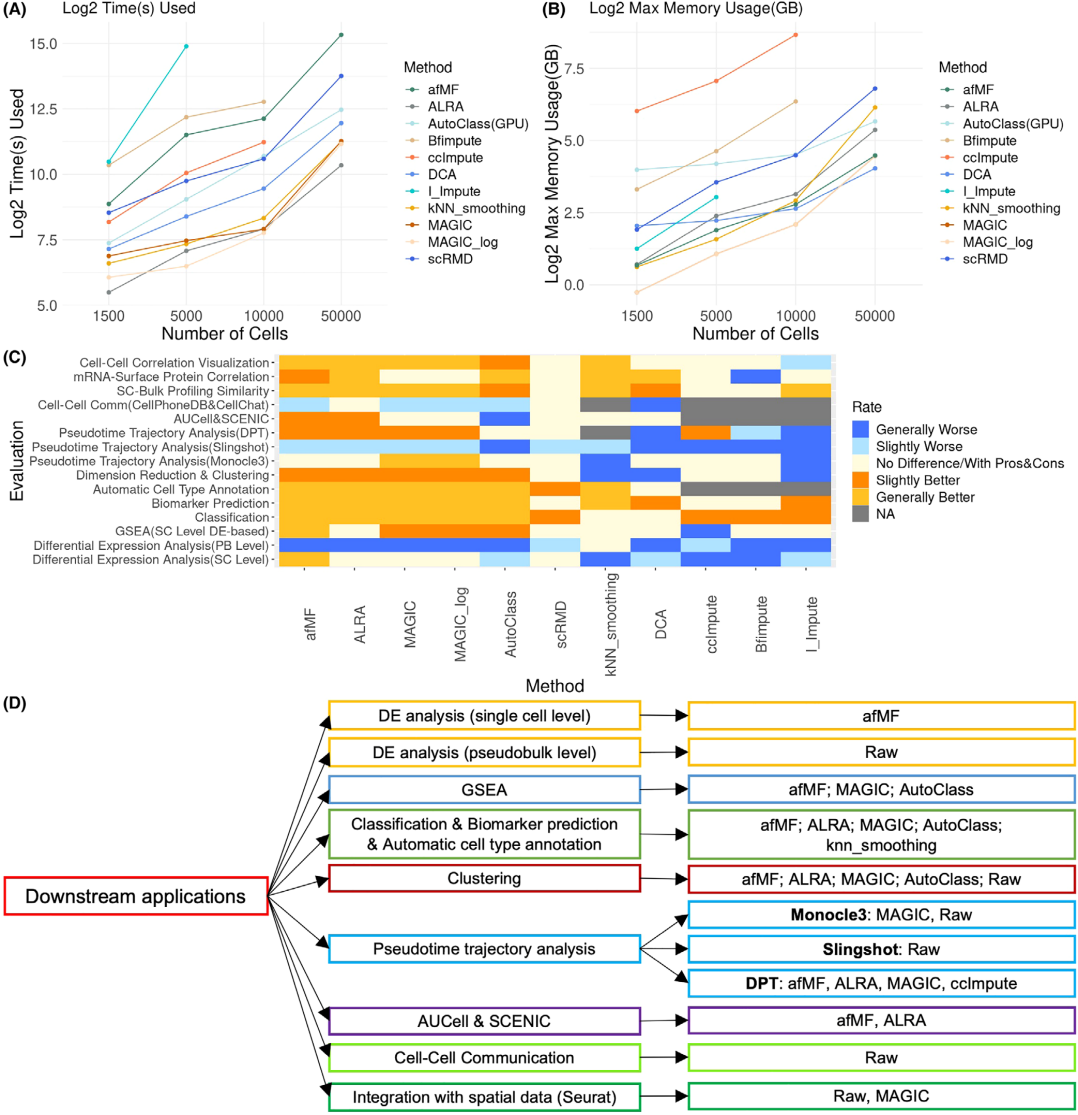
Running time, memory usage and recommendations for imputation algorithms. Four sizes of datasets (10 000× 1500, 10 000×5000, 10 000×10 000 and 10 000×50 000 matrix) were applied to evaluate the (A) time spent and (B) memory usage of different imputation methods. (C) On the basis of evaluations, a summary heatmap was created. The performances were classified into five levels ‘Generally Worse’, ‘Slightly Worse’, ‘No Obvious Difference’, ‘Slightly Better’ and ‘Generally Better’. Note that algorithm‐evaluation were labelled as ‘No Obvious Difference’ if that algorithm had both obvious advantages and disadvantages based on different metrics in that evaluation. (D) Recommendations of imputation algorithms for various downstream analyses.

In this study, we developed an exhaustive benchmark framework for scRNA‐seq imputations and an improved algorithm afMF to handle dropouts. afMF had great and stable performance while kept acceptable scalability. We hope these works can enhance the use of imputation in various downstream tasks as a complement to raw data analysis, and further promote new discoveries. We also have some further discussions in Note S10.

## AUTHOR CONTRIBUTIONS

Jinghan Huang contributed to the design of the work, performed data curation, analysis, interpretation of data, and the creation of new software used in the work, and drafted and revised the work. Anson C. M. Chow contributed to the design of the algorithm and the creation of new software used in the work. Nelson L. S. Tang conceived the research, contributed to the design of the work, interpretation of data, and drafted and revised the work. Sheung Chi Phillip Yam contributed to the conception and design of the algorithm. All authors read and approved the final manuscript.

## CONFLICT OF INTEREST STATEMENT

NT is founding Director and shareholder of the biotechstartup company, Cytomics Ltd, in Hong Kong Science Park. AC was an part‐time employee of Cytomics Ltd during the development of this software afMF.

## FUNDING INFORMATION

Phillip Yam acknowledges the financial supports from HKGRF‐14301321 with the project title “General Theory for InfiniteDimensional Stochastic Control: Mean Field and Some Classical Problems” and HKGRF‐14300123 with the project title“Well‐posedness of SomePoisson‐driven Mean Field Learning Models and their Applications”. He is also supported by a grant from the Germany/Hong Kong Joint Research Scheme funded by the Research Grants Council of Hong Kong and the German Academic Exchange Service of Germany (G‐CUHK411/23) and visiting professor supported by The University of Texas at Dallas, Naveen Jindal School of Management.

## ETHICS APPROVAL

The authors have nothing to report.

## Supporting information



Supporting Information

Supporting Information

Supporting Information

Supporting Information

Supporting Information

Supporting Information

Supporting Information

Supporting Information

Supporting Information

Supporting Information

## Data Availability

The data used in this study are available in public data domains (e.g., GEO and EGA). All public data analyzed during this study are shown in Additional file 1 Table S1. The code for the benchmark evaluations and the new imputation algorithm ‘afMF’ is available at: https://github.com/GO3295/SCImputation. The simulated data used in this study is available at: https://zenodo.org/records/14848073.
