# Supplementary material for: An in‐depth benchmark framework for evaluating single cell RNA‐seq dropout imputation methods and the development of an improved algorithm afMF
Source: Clin Transl Med. 2025 Mar 22;15(4):e70283. doi: 10.1002/ctm2.70283 (PMC11928879; doi:10.1002/ctm2.70283)

**Method S1. Datasets, Algorithms, pre-processing, and pre-screening**

*Datasets and Algorithms*

Datasets including scRNA-seq, bulk RNA-seq and other related datasets used in this study were collected from multiple public resources^1,2–19^ (**Table S1**). Basically, the datasets we used should have at least one of the following features, depending on the downstream tasks: (1) well-established, high-quality datasets that have been validated and used by other benchmark studies; (2) cells with ground truth labels (e.g., cell types, time course) generated or validated by wet-lab experiments or other well-established omics markers; (3) with matched cell line bulk data as near-ground-truth for some specific downstream tasks (e.g., differential expression analysis between cell types, GSEA); (4) cell line data should not have other complicated design (e.g., biological conditions), which may potentially complicate the evaluations of clustering; (5) real-world large datasets for some specific tasks (e.g., automatic cell type annotation, SCENIC) but with less than 60,000 cells due to limited space and scalability.

In order to have ground truth or near ground truth in real data analysis, we preferentially selected datasets from three kinds of experiments, including (1) cell line mixture of known cell types and gene expression profile of component cell types that have been well understood by bulk RNA-seq; (2) cell-cycle/time experiment with well-known checkpoint description. By this approach, downstream analyses can be confirmed and compared according to characteristics of component cell lines (near ground-truth is available). (3) Datasets with individual cells measured for additional ground truth data, e.g., surface protein level. Unlike the data from various tissues or individuals, cell line data is more precise and with less variability and heterogeneity; therefore, the single cell has a better correlation with the bulk, i.e., group of cells from the same cell type. Besides, the cell type labels in each of the data were generated by either wet-lab experiments, or other multi-omics specific markers such as CITE-seq surface proteins, and have been rigorously validated by the authors. In addition to the real data, we also evaluated the methods on the simulated data, where the definite ground truth was available. These simulated datasets were generated by Splatter^20^ and SplatPop^21^. In some evaluations, datasets were divided into mixture/purified cell type and time-course data as they may have different levels of complexity for imputing.

To select imputation algorithms for evaluation, we reviewed literatures^22–24^ and listed twenty-one of them^1,25–42^ (**Table S2**). Basically, these algorithms were either demonstrated to have top performance in previous evaluations or were new algorithms with good design and documentation. Imputed data was generated by each software following the instructions with default parameters. For algorithms required number of clusters as input (e.g., Bfimpute and AutoClass), the number of labeled cell types were used. As a baseline for comparison, Seurat^43^ log-normalized data without imputation was used since it is the most common routine and was demonstrated to have great performance^44^.

*Data pre-processing*

Processed datasets were collected from previous studies. For raw datasets without quality control, we applied the following filtering criteria to remove: (1) cells expressed < 200 genes; (2) outlier cells based on number of genes expressed (nFeature_RNA), number of total counts (nCount_RNA), and percentage of mitochondrial gene expression (percent.mt); (3) Genes expressed in less than 10 cells. For raw data as comparison, Seurat log-normalization was performed. All the imputed data was log-transformed (i.e., either before or after imputing, depending on the software). Simulated datasets were generated using the parameters shown in **Table S1**.

*The improved imputation algorithm using Low-Rank Full Matrix Factorization: afMF and its implementation*

afMF (**a**daptive **f**ull **M**atrix **F**actorization) is an imputation algorithm that builds upon the methodology of another algorithm called ALRA. The algorithm begins by following ALRA's data preprocessing steps, which include normalization, log-transformation, and rank selection. While ALRA employs randomized SVD for imputation, afMF takes a different approach by utilizing full matrix factorization as the imputation method. Once the rank (k) has been chosen, afMF assumes that the target single-cell gene matrix (X) consists of two matrices (P and Q). The first matrix contains the latent information of genes, while the second matrix contains the relative cell trait information of each cell with respect to the hidden gene information.

To solve the two matrices, afMF employs an iterative gradient descent algorithm. The algorithm fixes one matrix and solves for the optimal values of the other matrix based on its first-order derivatives. This iterative process, which is the key feature of this algorithm, allows afMF to refine the matrices and progressively improve the imputation accuracy for the task of matrix completion. Moreover, the algorithms used randomized SVD to approximate the training target which may sacrifice the information enclosed in the original cell-gene information. afMF keeps the original matrix as training target to avoid any information loss with coordinate gradient descent to converge effectively. In the meantime, afMF employ the latent dimension selection methods from ALRA with enhanced training method with no information loss. Overall, afMF presents an adaptive approach to scRNA-seq imputation, combining the preprocessing steps of ALRA with its own full matrix factorization method. By leveraging iterative gradient descent, afMF iteratively updates the matrices to effectively reconstruct the missing values and enhance the quality of the imputed single-cell gene expression data. The iteration will be completed when the difference in norm between imputed matrix at time t and t-1 is smaller than 10^-4^.

After the iteration, the entire single cell gene expression matrix is reconstructed by matrix multiplication. Subsequently, it applies ALRA's approach to rescale the completed matrix product. As definition of biological zero is not clear-cut, only outliers lower than 3 sigma were assigned as biological zero.

The algorithm is available at GitHub: <https://github.com/GO3295/SCImputation>

The details are as follows:

**Regularization**

Penalty Term: $f= \lambda_{P}\left\| P \right\|_{F}^{2}+ \lambda_{Q}\left\| Q \right\|_{F}^{2}$

$J$

$$= \left\| \hat{X}-X \right\|_{F}^{2}+\lambda_{P}\left\| P \right\|_{F}^{2}+ \lambda_{Q}\left\| Q \right\|_{F}^{2}$$

Considering ${PQ}^{T}=\hat{X}$

$=tr\left( \left( {PQ}^{T}-X \right)^{T}\left( {PQ}^{T}-X \right) \right)+\lambda_{P}tr\left( {PP}^{T} \right)+\lambda_{Q}tr\left( {QQ}^{T} \right)$ $=tr\left( \left( {QP}^{T}-X^{T} \right)\left( {PQ}^{T}-X \right) \right)+\lambda_{P}tr\left( {PP}^{T} \right)+\lambda_{Q}tr\left( {QQ}^{T} \right)$ $=tr\left( {QP}^{T}{PQ}^{T}-{QP}^{T}X-X^{T}{PQ}^{T}+X^{T}X \right)+ \lambda_{P}tr\left( {PP}^{T} \right)+\lambda_{Q}tr\left( {QQ}^{T} \right)$ $=tr\left( {QP}^{T}{PQ}^{T} \right)-2tr\left( {QP}^{T}X \right)+tr\left( X^{T}X \right)+ \lambda_{P}tr\left( {PP}^{T} \right)+\lambda_{Q}tr\left( {QQ}^{T} \right)$

Initializing $P^{\left( 0 \right)}, Q^{\left( 0 \right)}$ as random matrix

For $P^{\left( i \right)}$

$$J = tr\left( P^{\left( i \right)}Q^{\left( i-1 \right)T}Q^{\left( i-1 \right)}P^{\left( i \right)T} \right) - 2tr\left( XQ^{\left( i-1 \right)}P^{\left( i \right)T} \right) +tr\left( X^{T}X \right)+\lambda_{P}tr\left( P^{\left( i \right)}P^{\left( t \right)T} \right)+\lambda_{Q}tr\left( Q^{\left( i-1 \right)}Q^{\left( i-1 \right)T} \right)\nabla_{P}J = 2P^{\left( i \right)}Q^{\left( i-1 \right)T}Q^{\left( i-1 \right)}-2XQ^{\left( i-1 \right)}+2\lambda_{P}P^{\left( i \right)}$$

$$\nabla_{P}J = 0 \to P^{\left( i \right)} = \left( XQ^{\left( i-1 \right)} \right)\left( Q^{\left( i-1 \right)T}Q^{\left( i-1 \right)} - \lambda_{P}I_{I} \right)^{-1}$$

For $Q^{\left( i \right)}$

$$J=tr\left( Q^{\left( i \right)}P^{\left( i \right)T}P^{\left( i \right)}Q^{\left( i \right)T} \right)-2tr\left( Q^{\left( i \right)}P^{\left( i \right)T}X \right)+tr\left( X^{T}X \right)+\lambda_{P}tr\left( P^{\left( i \right)}P^{\left( i \right)T} \right)+\lambda_{Q}tr\left( Q^{\left( i \right)}Q^{\left( i \right)T} \right)\nabla_{Q}J=2Q^{\left( i \right)}P^{\left( i \right)T}P^{\left( i \right)}-2X^{T}P^{\left( i \right)}+2\lambda_{Q}Q^{\left( i \right)}$$

$$\nabla_{Q}J=0 \to Q^{\left( i \right)} = \left( X^{T}P^{\left( i \right)} \right)\left( P^{\left( i \right)T}P^{\left( i \right)}-\lambda_{Q}I_{J} \right)^{-1}$$

*Selection of other short-listed imputation algorithms for detailed benchmarking with afMF*

Good imputation algorithms should have good robustness to any types of datasets. A pre-screening test including two datasets (GSE75748 and GSE81861) to select algorithms for further evaluations were performed. The pre-screening evaluations included: (1) Differential Expression (DE) Analysis using Rank Sum Test in pairwise cell type comparisons; (2) Biomarker Prediction (Area Under Curve) and Classification (Random Forest); (3) Clustering (Louvain Algorithms); (4) Single Cell-Bulk Profiling Similarity. Details were described in corresponding **Additional files**. Based on the results (**Figure S1**), eleven methods were used for further in-depth evaluations (**Table S2**).

*Other statistical analysis*

The coding work including imputation algorithms and evaluations was performed using R 4.2.1 or Python 3.8. For each of the evaluations with enough data, Wilcoxon Rank Sum test was performed between the results of the imputation algorithms and the unimputed log-normalization. The significances were indicated with ‘*’ or ‘.’, where one, two and three ‘*’ represented ‘P<0.05’, ‘P<0.01’ and ‘P<0.005’ respectively and ‘.’ represented ‘P<0.1’. Note that some imputation algorithms were not assessed in some of the evaluations due to the processing failures/errors, large memory usage or long running time.

**Note S1.**

Single cell RNA-seq (scRNA-seq) has been widely used in biomedical research in the last ten years. However, the ‘dropout’ (inflated zeros) problem in the data is still an un-resolved issue. Dropouts could be due to both biological and technical reasons. As previously discussed^22,45^, some researchers believed that the inflated zeros should be imputed so that downstream analyses could benefit from it^1,25,26^, while others insisted that imputations may introduce false-positive signals and those zeros may contain important information as well^46,47^. No definitive conclusion has been reached for whether and when imputation should be performed so far, and addressing this question would be beneficial to the biological community. From a biological perspective, we may expect no expression of many genes in particular cell types or conditions (biological zero); on the other hand, it is so common to see well-known housekeeping (HK) genes, e.g., GAPDH, have zero counts in cells caused by technical reasons (technical zero). The technical zeros, which do not represent the ground truth, are the prime targets to be handled by various imputation methods.

There are several origins of technical zeros. Technical zero could be due to sampling problem in a finite total number of reads in the setting of scRNA-seq or a wet-lab measurement error. It is not uncommon to see zero counts for many HK genes in scRNA-seq datasets. Specifically, HK genes account for a large proportion of transcripts inside a cell, and they are found universally among various cell types. Besides, cell type specific (CTS) marker genes are also present in a high abundance but are only confined in specific cell types. Both kinds of genes represent a high proportion of transcripts in a single cell. When random dropout happens to these genes, the downstream normalization and subsequent analysis will be adversely affected. These technical dropouts need to be taken care of to get more representative results in downstream analysis. Furthermore, the presence of zeros prevents log-transformation of the data and therefore investigators need to compromise by using pseudo-count addition rather than using the observed counts. Under these circumstances, imputation would be a more preferred alternative. Nonetheless, imputation is still not a popular approach in real-world applications since investigators have concerns about its effects on downstream applications and its use may probably result in incorrect or false positive inference.

Several imputation algorithms have been developed to recover those zeros in the past few years. These algorithms can be classified into three classes: (1) smoothing or model based, where various statistical distributions are assumed; (2) matrix-theory decomposition / factorization, which generally has good scalability and stable performance; (3) deep learning, which is more advanced, complicated, and currently emerging. For instance, MAGIC is an early and popular algorithm that is based on data smoothing. ALRA applies matrix decomposition with thresholding and shows a conserved but stable performance with outstanding scalability. The new algorithm ‘afMF’ developed in this study is an improved version of ALRA as it takes into account of iterative low-rank full matrix factorization. AutoClass is a deep-learning-based method and has been demonstrated to have some advantages. For now, scientists mainly focused on deep learning methods and foundation models^48,49^ for various scRNA-seq tasks including dropout imputation.

Here, we evaluated the compatibility between prior imputation algorithms and various downstream applications. There are some downstream tools specially designed for sparse data to account for the zero-inflation problems and thus imputation will be again redundant. An in-depth understanding of compatibility between prior imputation algorithms and downstream applications is urgently needed to better standardize scRNA-seq routine.

Therefore, we developed an improved benchmark framework to address these issues by including the following features (**Figure 1**): (1) 21 top or new algorithms with acceptable scalability; (2) more than 25 real (mixture/purified cell type/time-course) or simulated datasets; (3) a pre-screening test to select algorithms for further evaluations; (4) Visualizations (Gene Expression Violin plots; PCA/UMAP plots; Cell-Cell Correlations); (5) Differential Expression (DE) Analysis: Wilcoxon Rank Sum test, MAST^50^ and Pseudobulk DE analysis^51^; (6) Enrichment Analysis (GSEA)^52,53^; (7) Biomarker Prediction and Classification; (8) Automatic Cell Type Annotation: SCINA^54^ and scType^55^; (9) Dimension Reduction and Clustering; (10) Cell Cycle Dynamics; (11) Pseudotime Trajectory Analysis: Monocle3^56^, Slingshot^57^ and DPT^58^; (12) AUCell & SCENIC regulatory analysis^59^; (13) Cell-Cell Communication: CellPhoneDB^60^ & CellChat^61^; (14) Integration of spatial transcriptomics with scRNA-seq (Seurat); (15) Supporting Analysis: Single Cell-Bulk Profiling Similarity & Surface Protein-mRNA Correlation; (16) Running Time, Memory Usage and Recommendation. We used some of the datasets and evaluation metrics developed from previous works^23,24^ as their properties have been well described. To our knowledge, this is the first exhaustive comparative study to perform in-depth benchmark for imputation algorithms incorporating various popular downstream applications (i.e., pseudobulk DE analysis, GSEA, automatic cell type annotation, Monocle3, Slingshot, DPT, AUCell & SCENIC, cell-cell communication and integration of spatial transcriptomics with scRNA-seq) of scRNA-seq. In addition to this framework, based on our knowledges and evaluations we also developed an improved algorithm ‘afMF’ which has been evaluated together.

**Reference**

1. Linderman GC, Zhao J, Roulis M, et al. Zero-preserving imputation of single-cell RNA-seq data. *Nat Commun*. 2022;13(1):192. doi:10.1038/s41467-021-27729-z

2. Jerby-Arnon L, Shah P, Cuoco MS, et al. A Cancer Cell Program Promotes T Cell Exclusion and Resistance to Checkpoint Blockade. *Cell*. 2018;175(4):984-997.e24. doi:10.1016/j.cell.2018.09.006

3. Yang L, Wang WH, Qiu WL, Guo Z, Bi E, Xu CR. A single-cell transcriptomic analysis reveals precise pathways and regulatory mechanisms underlying hepatoblast differentiation. *Hepatology*. 2017;66(5):1387-1401. doi:10.1002/hep.29353

4. Angelidis I, Simon LM, Fernandez IE, et al. An atlas of the aging lung mapped by single cell transcriptomics and deep tissue proteomics. *Nat Commun*. 2019;10(1):963. doi:10.1038/s41467-019-08831-9

5. Tian L, Dong X, Freytag S, et al. Benchmarking single cell RNA-sequencing analysis pipelines using mixture control experiments. *Nat Methods*. 2019;16(6):479-487. doi:10.1038/s41592-019-0425-8

6. Vladoiu MC, El-Hamamy I, Donovan LK, et al. Childhood cerebellar tumours mirror conserved fetal transcriptional programs. *Nature*. 2019;572(7767):67-73. doi:10.1038/s41586-019-1158-7

7. Shumliakivska M, Luxán G, Hemmerling I, et al. DNMT3A clonal hematopoiesis-driver mutations induce cardiac fibrosis by paracrine activation of fibroblasts. *Nat Commun*. 2024;15(1):606. doi:10.1038/s41467-023-43003-w

8. Semrau S, Goldmann JE, Soumillon M, Mikkelsen TS, Jaenisch R, van Oudenaarden A. Dynamics of lineage commitment revealed by single-cell transcriptomics of differentiating embryonic stem cells. *Nat Commun*. 2017;8(1):1096. doi:10.1038/s41467-017-01076-4

9. Leng N, Chu LF, Barry C, et al. Oscope identifies oscillatory genes in unsynchronized single-cell RNA-seq experiments. *Nat Methods*. 2015;12(10):947-950. doi:10.1038/nmeth.3549

10. Torre E, Dueck H, Shaffer S, et al. Rare Cell Detection by Single-Cell RNA Sequencing as Guided by Single-Molecule RNA FISH. *Cell Syst*. 2018;6(2):171-179.e5. doi:10.1016/j.cels.2018.01.014

11. Li H, Courtois ET, Sengupta D, et al. Reference component analysis of single-cell transcriptomes elucidates cellular heterogeneity in human colorectal tumors. *Nat Genet*. 2017;49(5):708-718. doi:10.1038/ng.3818

12. Holik AZ, Law CW, Liu R, et al. RNA-seq mixology: designing realistic control experiments to compare protocols and analysis methods. *Nucleic Acids Res*. 2017;45(5):e30. doi:10.1093/nar/gkw1063

13. Vento-Tormo R, Efremova M, Botting RA, et al. Single-cell reconstruction of the early maternal-fetal interface in humans. *Nature*. 2018;563(7731):347-353. doi:10.1038/s41586-018-0698-6

14. Chu LF, Leng N, Zhang J, et al. Single-cell RNA-seq reveals novel regulators of human embryonic stem cell differentiation to definitive endoderm. *Genome Biol*. 2016;17(1):173. doi:10.1186/s13059-016-1033-x

15. Cano-Gamez E, Soskic B, Roumeliotis TI, et al. Single-cell transcriptomics identifies an effectorness gradient shaping the response of CD4+ T cells to cytokines. *Nat Commun*. 2020;11(1):1801. doi:10.1038/s41467-020-15543-y

16. Sun N, Akay LA, Murdock MH, et al. Single-nucleus multiregion transcriptomic analysis of brain vasculature in Alzheimer’s disease. *Nat Neurosci*. 2023;26(6):970-982. doi:10.1038/s41593-023-01334-3

17. Arunachalam PS, Wimmers F, Mok CKP, et al. Systems biological assessment of immunity to mild versus severe COVID-19 infection in humans. *Science*. 2020;369(6508):1210-1220. doi:10.1126/science.abc6261

18. ENCODE Project Consortium. The ENCODE (ENCyclopedia Of DNA Elements) Project. *Science*. 2004;306(5696):636-640. doi:10.1126/science.1105136

19. Tasic B, Menon V, Nguyen TN, et al. Adult mouse cortical cell taxonomy revealed by single cell transcriptomics. *Nat Neurosci*. 2016;19(2):335-346. doi:10.1038/nn.4216

20. Zappia L, Phipson B, Oshlack A. Splatter: simulation of single-cell RNA sequencing data. *Genome Biol*. 2017;18(1):174. doi:10.1186/s13059-017-1305-0

21. Azodi CB, Zappia L, Oshlack A, McCarthy DJ. splatPop: simulating population scale single-cell RNA sequencing data. *Genome Biol*. 2021;22(1):341. doi:10.1186/s13059-021-02546-1

22. Jiang R, Sun T, Song D, Li JJ. Statistics or biology: the zero-inflation controversy about scRNA-seq data. *Genome Biol*. 2022;23(1):31. doi:10.1186/s13059-022-02601-5

23. Hou W, Ji Z, Ji H, Hicks SC. A systematic evaluation of single-cell RNA-sequencing imputation methods. *Genome Biol*. 2020;21(1):218. doi:10.1186/s13059-020-02132-x

24. Dai C, Jiang Y, Yin C, et al. scIMC: a platform for benchmarking comparison and visualization analysis of scRNA-seq data imputation methods. *Nucleic Acids Res*. 2022;50(9):4877-4899. doi:10.1093/nar/gkac317

25. van Dijk D, Sharma R, Nainys J, et al. Recovering Gene Interactions from Single-Cell Data Using Data Diffusion. *Cell*. 2018;174(3):716-729.e27. doi:10.1016/j.cell.2018.05.061

26. Li H, Brouwer CR, Luo W. A universal deep neural network for in-depth cleaning of single-cell RNA-Seq data. *Nat Commun*. 2022;13(1):1901. doi:10.1038/s41467-022-29576-y

27. Eraslan G, Simon LM, Mircea M, Mueller NS, Theis FJ. Single-cell RNA-seq denoising using a deep count autoencoder. *Nat Commun*. 2019;10(1):390. doi:10.1038/s41467-018-07931-2

28. Wen ZH, Langsam JL, Zhang L, Shen W, Zhou X. A Bayesian factorization method to recover single-cell RNA sequencing data. *Cell Rep Methods*. 2022;2(1):100133. doi:10.1016/j.crmeth.2021.100133

29. Xu C, Cai L, Gao J. An efficient scRNA-seq dropout imputation method using graph attention network. *BMC Bioinformatics*. 2021;22(1):582. doi:10.1186/s12859-021-04493-x

30. Malec M, Kurban H, Dalkilic M. ccImpute: an accurate and scalable consensus clustering based algorithm to impute dropout events in the single-cell RNA-seq data. *BMC Bioinformatics*. 2022;23(1):291. doi:10.1186/s12859-022-04814-8

31. Lopez R, Regier J, Cole MB, Jordan MI, Yosef N. Deep generative modeling for single-cell transcriptomics. *Nat Methods*. 2018;15(12):1053-1058. doi:10.1038/s41592-018-0229-2

32. Arisdakessian C, Poirion O, Yunits B, Zhu X, Garmire LX. DeepImpute: an accurate, fast, and scalable deep neural network method to impute single-cell RNA-seq data. *Genome Biol*. 2019;20(1):211. doi:10.1186/s13059-019-1837-6

33. Feng X, Chen L, Wang Z, Li SC. I-Impute: a self-consistent method to impute single cell RNA sequencing data. *BMC Genomics*. 2020;21(Suppl 10):618. doi:10.1186/s12864-020-07007-w

34. Butler A, Hoffman P, Smibert P, Papalexi E, Satija R. Integrating single-cell transcriptomic data across different conditions, technologies, and species. *Nat Biotechnol*. 2018;36(5):411-420. doi:10.1038/nbt.4096

35. Wagner F, Yan Y, Yanai I. *K-Nearest Neighbor Smoothing for High-Throughput Single-Cell RNA-Seq Data*. Bioinformatics; 2017. doi:10.1101/217737

36. Leote AC, Wu X, Beyer A. Regulatory network-based imputation of dropouts in single-cell RNA sequencing data. *PLoS Comput Biol*. 2022;18(2):e1009849. doi:10.1371/journal.pcbi.1009849

37. Tracy S, Yuan GC, Dries R. RESCUE: imputing dropout events in single-cell RNA-sequencing data. *BMC Bioinformatics*. 2019;20(1):388. doi:10.1186/s12859-019-2977-0

38. Gu H, Cheng H, Ma A, et al. scGNN 2.0: a graph neural network tool for imputation and clustering of single-cell RNA-Seq data. *Bioinformatics*. 2022;38(23):5322-5325. doi:10.1093/bioinformatics/btac684

39. Xu Y, Zhang Z, You L, Liu J, Fan Z, Zhou X. scIGANs: single-cell RNA-seq imputation using generative adversarial networks. *Nucleic Acids Res*. 2020;48(15):e85. doi:10.1093/nar/gkaa506

40. Chen C, Wu C, Wu L, Wang X, Deng M, Xi R. scRMD: imputation for single cell RNA-seq data via robust matrix decomposition. *Bioinformatics*. 2020;36(10):3156-3161. doi:10.1093/bioinformatics/btaa139

41. Pu J, Wang B, Liu X, Chen L, Li SC. SMURF: embedding single-cell RNA-seq data with matrix factorization preserving self-consistency. *Brief Bioinform*. 2023;24(2):bbad026. doi:10.1093/bib/bbad026

42. Wu X, Zhou Y. GE-Impute: graph embedding-based imputation for single-cell RNA-seq data. *Brief Bioinform*. 2022;23(5):bbac313. doi:10.1093/bib/bbac313

43. Hao Y, Hao S, Andersen-Nissen E, et al. Integrated analysis of multimodal single-cell data. *Cell*. 2021;184(13):3573-3587.e29. doi:10.1016/j.cell.2021.04.048

44. Ahlmann-Eltze C, Huber W. Comparison of transformations for single-cell RNA-seq data. *Nat Methods*. 2023;20(5):665-672. doi:10.1038/s41592-023-01814-1

45. Brooks TG, Lahens NF, Mrčela A, Grant GR. Challenges and best practices in omics benchmarking. *Nat Rev Genet*. Published online January 12, 2024. doi:10.1038/s41576-023-00679-6

46. Qiu P. Embracing the dropouts in single-cell RNA-seq analysis. *Nat Commun*. 2020;11(1):1169. doi:10.1038/s41467-020-14976-9

47. Kim TH, Zhou X, Chen M. Demystifying “drop-outs” in single-cell UMI data. *Genome Biol*. 2020;21(1):196. doi:10.1186/s13059-020-02096-y

48. Cui H, Wang C, Maan H, et al. scGPT: toward building a foundation model for single-cell multi-omics using generative AI. *Nat Methods*. 2024;21(8):1470-1480. doi:10.1038/s41592-024-02201-0

49. Hao M, Gong J, Zeng X, et al. Large-scale foundation model on single-cell transcriptomics. *Nat Methods*. 2024;21(8):1481-1491. doi:10.1038/s41592-024-02305-7

50. Finak G, McDavid A, Yajima M, et al. MAST: a flexible statistical framework for assessing transcriptional changes and characterizing heterogeneity in single-cell RNA sequencing data. *Genome Biol*. 2015;16:278. doi:10.1186/s13059-015-0844-5

51. Squair JW, Gautier M, Kathe C, et al. Confronting false discoveries in single-cell differential expression. *Nat Commun*. 2021;12(1):5692. doi:10.1038/s41467-021-25960-2

52. Subramanian A, Tamayo P, Mootha VK, et al. Gene set enrichment analysis: a knowledge-based approach for interpreting genome-wide expression profiles. *Proc Natl Acad Sci U S A*. 2005;102(43):15545-15550. doi:10.1073/pnas.0506580102

53. Yu G, Wang LG, Han Y, He QY. clusterProfiler: an R package for comparing biological themes among gene clusters. *OMICS*. 2012;16(5):284-287. doi:10.1089/omi.2011.0118

54. Zhang Z, Luo D, Zhong X, et al. SCINA: A Semi-Supervised Subtyping Algorithm of Single Cells and Bulk Samples. *Genes (Basel)*. 2019;10(7):531. doi:10.3390/genes10070531

55. Ianevski A, Giri AK, Aittokallio T. Fully-automated and ultra-fast cell-type identification using specific marker combinations from single-cell transcriptomic data. *Nat Commun*. 2022;13(1):1246. doi:10.1038/s41467-022-28803-w

56. Qiu X, Mao Q, Tang Y, et al. Reversed graph embedding resolves complex single-cell trajectories. *Nat Methods*. 2017;14(10):979-982. doi:10.1038/nmeth.4402

57. Street K, Risso D, Fletcher RB, et al. Slingshot: cell lineage and pseudotime inference for single-cell transcriptomics. *BMC Genomics*. 2018;19(1):477. doi:10.1186/s12864-018-4772-0

58. Haghverdi L, Büttner M, Wolf FA, Buettner F, Theis FJ. Diffusion pseudotime robustly reconstructs lineage branching. *Nat Methods*. 2016;13(10):845-848. doi:10.1038/nmeth.3971

59. Aibar S, González-Blas CB, Moerman T, et al. SCENIC: single-cell regulatory network inference and clustering. *Nat Methods*. 2017;14(11):1083-1086. doi:10.1038/nmeth.4463

60. Efremova M, Vento-Tormo M, Teichmann SA, Vento-Tormo R. CellPhoneDB: inferring cell-cell communication from combined expression of multi-subunit ligand-receptor complexes. *Nat Protoc*. 2020;15(4):1484-1506. doi:10.1038/s41596-020-0292-x

61. Jin S, Guerrero-Juarez CF, Zhang L, et al. Inference and analysis of cell-cell communication using CellChat. *Nat Commun*. 2021;12(1):1088. doi:10.1038/s41467-021-21246-9

**Table S1. Datasets used in this study.**

| **Datasets** | **Type** | **Description** | **Evaluation** |
| --- | --- | --- | --- |
| **CellBench 10x 5cl (GSE126906)** | Single cell RNA-seq | Cell mixture sample of 5 cell lines H2228, H1975, A549, H838 and HCC827. | Differential Expression Analysis; GSEA Analysis; Biomarker Prediction & Classification; Clustering; SC-Bulk Similarity. |
| **GSE86337** | Bulk RNA-seq | Bulk samples of 5 cell lines H2228, H1975, HCC827, H838 and A549. | Differential Expression Analysis; GSEA Analysis; Biomarker Prediction; SC-Bulk Similarity. |
| **GSE81861** | Single cell RNA-seq | Cell mixture sample of 5 cell lines A549, GM12878, H1-hESC, IMR90, and K562. | Differential Expression Analysis; GSEA Analysis; Biomarker Prediction & Classification; Clustering; SC-Bulk Similarity. |
| **ENCODE bulk RNA-seq** | Bulk RNA-seq | Bulk samples of 5 cell lines A549, GM12878, H1-hESC, IMR90, and K562. | Differential Expression Analysis; GSEA Analysis; Biomarker Prediction; SC-Bulk Similarity. |
| **GSE75748** | Single cell RNA-seq & Bulk RNA-seq | Cell mixture sample and bulk sample of 7 cell types DEC, EC, H1, H9, HFF, NPC and TB; Time course profiling single cell/bulk sample using H1. | Differential Expression Analysis; GSEA Analysis; Biomarker Prediction & Classification; Clustering; Pseudotime Trajectory Analysis; SC-Bulk Similarity. |
| **GSE124872** | Single cell RNA-seq & Bulk RNA-seq | Alveolar macrophages and type-2 pneumocytes single cell/bulk samples from 24 months and 3 months old mice. | Differential Expression Analysis; GSEA Analysis; Biomarker Prediction & Classification; SC-Bulk Similarity. |
| **EGAS00001003215 and EGAS00001003823** | Single cell RNA-seq & Bulk RNA-seq | Naive or memory T cells (Th0; Th2; Th17; iTreg) single cell/bulk samples: stimulated for 5 d with anti-CD3/anti-CD28 coated beads or resting. | Differential Expression Analysis; GSEA Analysis; Biomarker Prediction & Classification; SC-Bulk Similarity. |
| **GSE79578** | Single cell RNA-seq & Bulk RNA-seq | Mouse embryonic stem cells after different periods of continuous exposure to retinoic acid. | Differential Expression Analysis, Pseudotime Trajectory Analysis; Classification; GSEA analysis. |
| **GSE90047** | Single cell RNA-seq & Bulk RNA-seq | Sorted hepatoblasts, hepatocytes and cholangiocytes from E10.5-E17.5 mouse fetal livers. | Differential Expression Analysis, Pseudotime Trajectory Analysis; Classification; GSEA analysis. |
| **GSE155673** | Single cell RNA-seq | Cells from PBMC with labeled cell types and disease status (COVID-19 and healthy controls). | Differential Expression Analysis & GSEA analysis (real application for COVID-19); Automatic Cell Type Annotation; AUCell. |
| **brain vasculature**  [**http://compbio.mit.edu/scADbbb/**](http://compbio.mit.edu/scADbbb/) | Single cell RNA-seq | Human brain tissues from the ROSMAP with cell types labeled. | Automatic Cell Type Annotation. |
| **GSE64016** | Single cell RNA-seq | H1 and H1-Fucci single cells | Cell Cycle Dynamics. |
| **CellBench cellmix1-4 (GSE118704)** | Single cell RNA-seq | 9 cell mixtures with differentiation information from three cell lines H2228, H1975 and HCC827. | Pseudotime Trajectory Analysis. |
| **GSE118068** | Single cell RNA-seq | Nine mouse hindbrains from different developmental stages (embryonic days and postnatal days). | Pseudotime Trajectory Analysis. |
| **GSE115978** | Single cell RNA-seq | Cells from fresh tumor resections, isolated immune and non-immune cells by FACS based on CD45 staining. Cell types were pre-labeled. | SCENIC |
| **E-MTAB-6701** | Single cell RNA-seq | Cells from matched first trimester samples of maternal blood and decidua, as well as fetal cells from the placenta itself. | CellPhoneDB |
| **E-MTAB-13384** | Single cell RNA-seq | Cardiac muscle cell from mice with WT/DNMT3AR882H knock-in | CellChat |
| **stxBrain (**[**https://support.10xgenomics.com/spatial-gene-expression/datasets**](https://support.10xgenomics.com/spatial-gene-expression/datasets)**)** | Spatial transcriptomics | Sagital mouse brain slices generated using the Visium v1 chemistry. Anterior sections were used. | Integration of spatial transcriptomics with scRNA-seq |
| **GSE71585** | Single cell RNA-seq | Adult mouse cortical cell taxonomy generated with the SMART-Seq2 protocol. | Integration of spatial transcriptomics with scRNA-seq |
| **GSE100866** | Single cell RNA-seq | Cord blood mononuclear cells were profiled by CITE-seq using a panel of 13 antibodies. | Surface Protein-mRNA Correlation |
| **Simulated dataset1 (Mock90)** | Single cell RNA-seq (Simulated) | Splatter: nGenes=10000, batchCells=1500, group.prob=c(0.3,0.25,0.2,0.15,0.1); set dropout.mid to let dropout rate=90%. Others are default. | Differential Expression Analysis; Biomarker Prediction; Classification; Automatic Cell Type Annotation; Clustering; SC-Ground Truth Similarity; Distinguish dropouts and real zeros. |
| **Simulated dataset2 (SplatPop90)** | Single cell RNA-seq (Simulated) | SplatPop: nGenes=8000, batchCells=200, group.prob=c(0.4,0.3,0.3), condition.prob=c(0.5,0.5), similarity.scale=15, de.facLoc=0.5, de.facScale=0.5, cde.facLoc=0.38, cde.facScale=0.38, set dropout.mid to let dropout rate=90%. Others are default. | Differential Expression Analysis; Biomarker Prediction & Classification; Automatic Cell Type Annotation; Clustering. |

Twenty-four real datasets and two simulated datasets (i.e., Splatter and SplatPop) were used in various downstream analyses. In some evaluations, datasets were divided into mixture, purified cell type data and time-course data as they may have different levels of complexity.

**Table S2. Methods used in this study.**

| **Class of method** | **Methods**† | **Platform** | **DOI and Reference** | **Post-logarithm** | **Further Evaluation*** |
| --- | --- | --- | --- | --- | --- |
| Baseline: no imputation (labeled as raw) | **Log-normalization (Seurat)** | R | https://doi.org/10.1016/j.cell.2019.05.031 ^43^ | No | Yes |
| Matrix decomposition / factorization | **afMF** | Python | Developed in this study | No | Yes |
| Matrix decomposition / factorization | **ALRA** | R | https://doi.org/10.1038/s41467-021-27729-z ^1^ | No | Yes |
| Matrix decomposition / factorization | **Bfimpute** | R | <https://doi.org/10.1016/j.crmeth.2021.100133> ^28^ | No | Yes |
| Matrix decomposition / factorization | **scRMD (raw and log)** | R | <https://doi.org/10.1093/bioinformatics/btaa139> ^40^ | Yes/No | Yes |
| Matrix decomposition / factorization | SMURF | Python | <https://doi.org/10.1093/bib/bbad026> ^41^ | Yes | No |
| Model and smoothing based | ADimpute | R | <https://doi.org/10.1371/journal.pcbi.1009849> ^36^ | No | No |
| Model and smoothing based | **ccImpute** | R | <https://doi.org/10.1186/s12859-022-04814-8> ^30^ | No | Yes |
| Model and smoothing based | **I-impute** | Python | <https://doi.org/10.1186/s12864-020-07007-w> ^33^ | Yes | Yes |
| Model and smoothing based | **knn-smoothing (KS)** | Python | <https://doi.org/10.1101/217737> ^35^ | Yes | Yes |
| Model and smoothing based | **MAGIC (square root and log)** | Python | <https://doi.org/10.1016/j.cell.2018.05.061> ^25^ | No | Yes |
| Model and smoothing based | RESCUE | R | <https://doi.org/10.1186/s12859-019-2977-0> ^37^ | No | No |
| Deep learning based | **AutoClass** | Python | <https://doi.org/10.1038/s41467-022-29576-y> ^26^ | No | Yes |
| Deep learning based | scGNN2 | Python | <https://doi.org/10.1093/bioinformatics/btac684> ^38^ | No | No |
| Deep learning based | scIGANs | Python | <https://doi.org/10.1093/nar/gkaa506> ^39^ | Yes | No |
| Deep learning based | **DCA** | Python | <https://doi.org/10.1038/s41467-018-07931-2> ^27^ | No | Yes |
| Deep learning based | scVI | Python | <https://doi.org/10.1038/s41592-018-0229-2> ^31^ | Yes | No |
| Deep learning based | deepimpute | Python | <https://doi.org/10.1186/s13059-019-1837-6> ^32^ | Yes | No |
| Deep learning based | GNNimpute | Python | <https://doi.org/10.1186/s12859-021-04493-x> ^29^ | Yes | No |
| Deep learning based | GE-Impute | Python | <https://doi.org/10.1093/bib/bbac313> ^42^ | No | No |

Twenty-two methods including unimputed log-normalization (Seurat) and twenty-one imputation algorithms based on R or Python were evaluated in this study.

† Ten algorithms showed in Bold Font passed the pre-screening test and were selected for further in-depth evaluations.

**Figure S1. Pre-screening for twenty-one imputation algorithms**


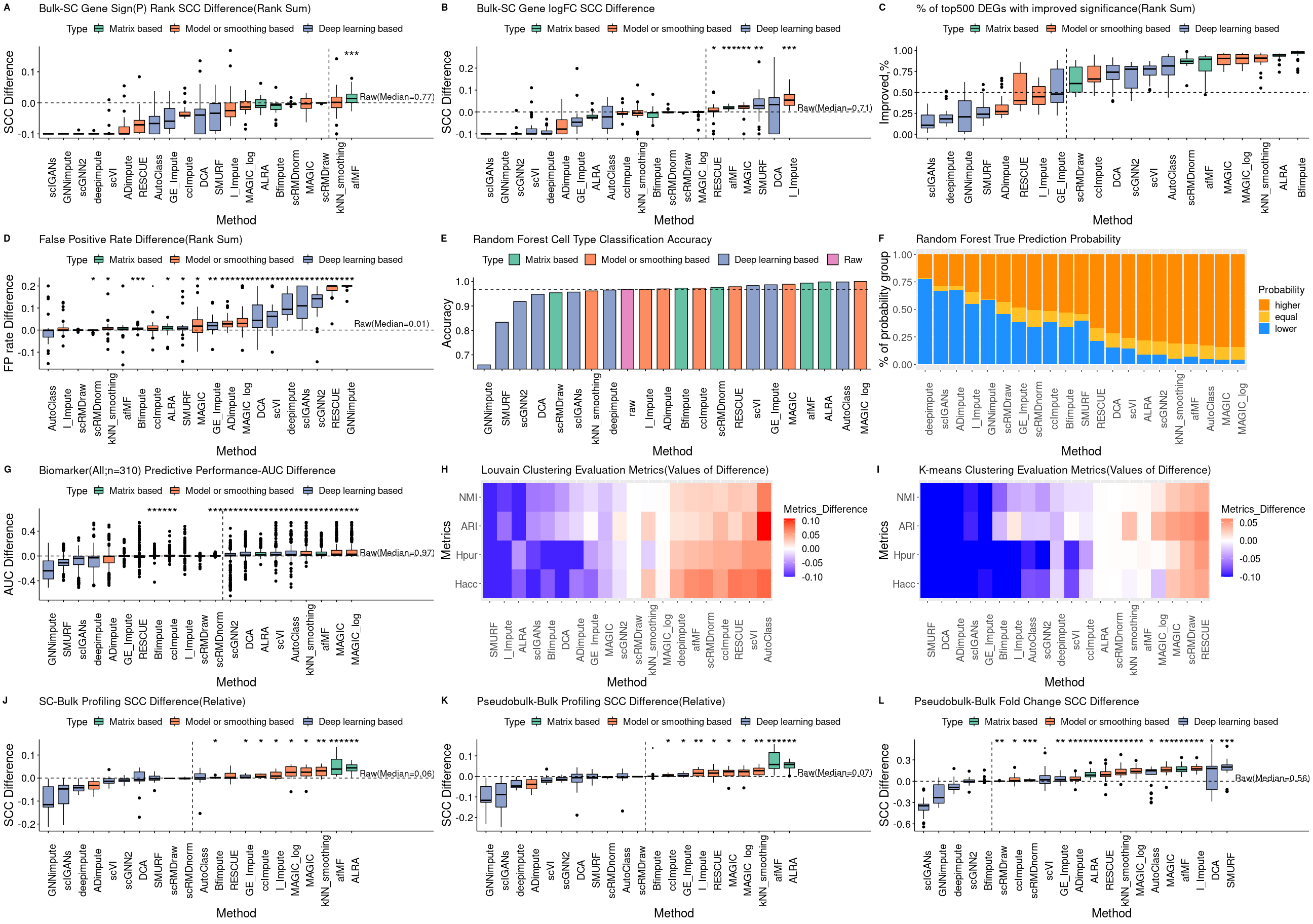

Supplement: Supplementary file 1 — Supporting Information [file CTM2-15-e70283-s003.docx]
