# Supplementary material for: An in‐depth benchmark framework for evaluating single cell RNA‐seq dropout imputation methods and the development of an improved algorithm afMF
Source: Clin Transl Med. 2025 Mar 22;15(4):e70283. doi: 10.1002/ctm2.70283 (PMC11928879; doi:10.1002/ctm2.70283)
Supplement: Supplementary file 2 — Supporting Information [file CTM2-15-e70283-s006.docx]

**Method S2. Visualizations.**

*Gene Expression Violin plots and Cell-Cell Correlation visualizations*

Initially, we explored how the gene expression visualization changed when using various imputation methods. We used GSE155673 and plotted GAPDH, CD8A and CD19 for comparing both housekeeping gene and cell type specific markers across different cell types. The Seurat function VlnPlot() was applied.

We next calculated the cell-cell correlations in CellBench-10X5CL to study if imputation can increase cell-cell similarity within same cell types and improve visualizations. Spearman correlation coefficients were calculated between pairwise cells and results were visualized through heatmaps using R pheatmap function.

**Note S2.**

We observed a heavy zero tail for, e.g., B cells and NK cells in the violin plots of log-count of the housekeeping gene GAPDH; in contrast, after afMF/ALRA imputation, these dropouts were largely ameliorated (**Figure S2A**). For marker genes CD8A and CD19, afMF provided decent distributions in which the marker genes were uniquely higher expressed in CD8+ T cells and B cells respectively with no heavy tail toward zero (**Figure S2A**). DCA is likely to overfit and distort the data. Next, we examined the potential consequence of imputation in generating artefactual structure in low-dimensional space and visualized in 2-D PCA (**Figure S2B** and **Figure S3-4**). Compared to the ground truth (‘true’), raw and many algorithms including afMF could separate Group4 and Group5 without fabricating additional false structure in PCA (**Figure S3**). However, ccImpute, I-impute and MAGIC fabricated fortuitous structure on the PCA embeddings. On the other hand, both Bfimpute and kNN-smoothing provided concentrated clusters with low variance and even outperformed the ground truth, which is likely to be the overfitting problem. Fabrication features were also seen in a dataset generated by SplatPop imputed by AutoClass and Bfimpute (**Figure S4**). Combining these results, only afMF, ALRA and scRMD did not fabricate artefactual structure in PCA. We next visualized cell-cell correlations (**Figure S2C** and **Figure S5**). afMF and other five algorithms showed better separations and patterns for different cell types.

**Figure S2. The impact of imputations on the dropouts: gene expression violin plots, 2-D PCA, and cell-cell correlation heatmaps**

**
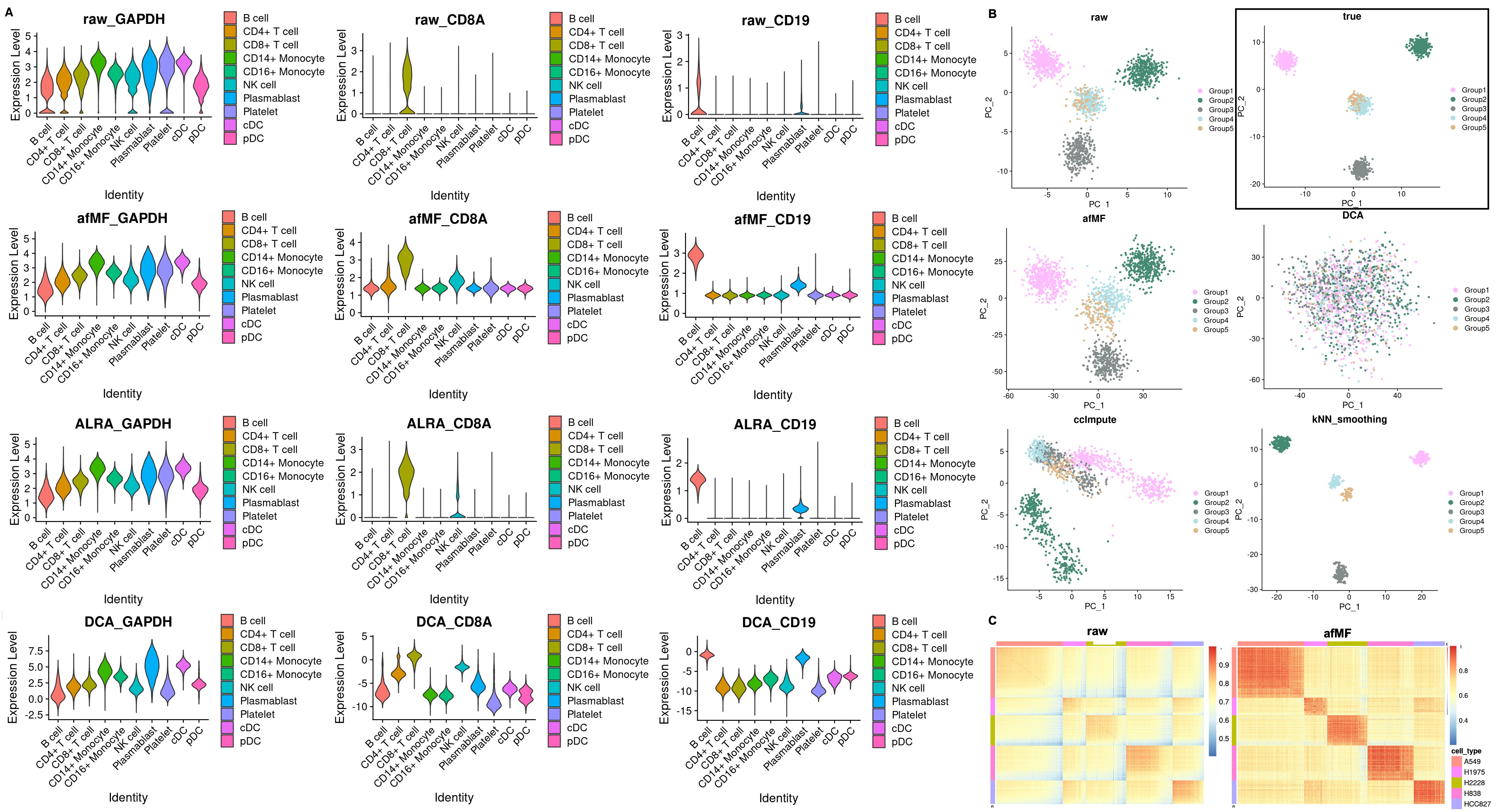
**

The impact of imputations on the dropouts and related visualizations. (**A**) The impact of different imputation algorithms on gene expression were visualized through violin plots across different cell types in dataset GSE155673 using housekeeping gene GAPDH and cell type specific gene CD8A and CD19. (**B**) The impact of different imputation algorithms on 2-D PCA were visualized using simulated dataset Mock90 (dropout rate=90%). Results from the raw (not imputed) and the true (ground truth) data were also shown. (**C**) The impact of different imputation algorithms on cell-cell correlations were visualized through heatmaps by calculating the Spearman correlation coefficients (SCCs) between pairwise cells in dataset CellBench-10X5CL.

**Figure S3. 2-D PCA plots for simulated dataset Mock90**

**
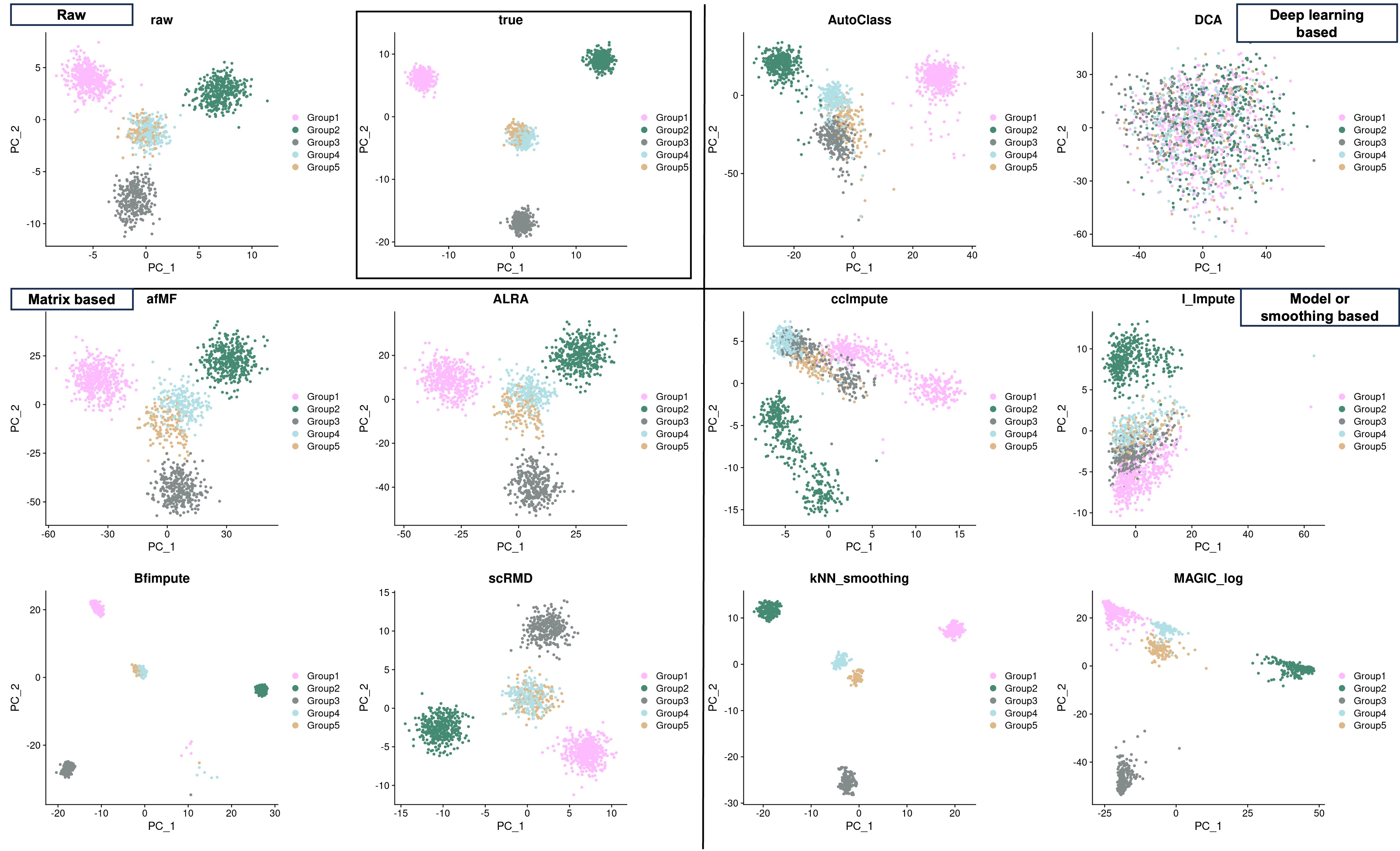
**

**Figure S4. 2-D PCA plots for simulated dataset SplatPop90**

**
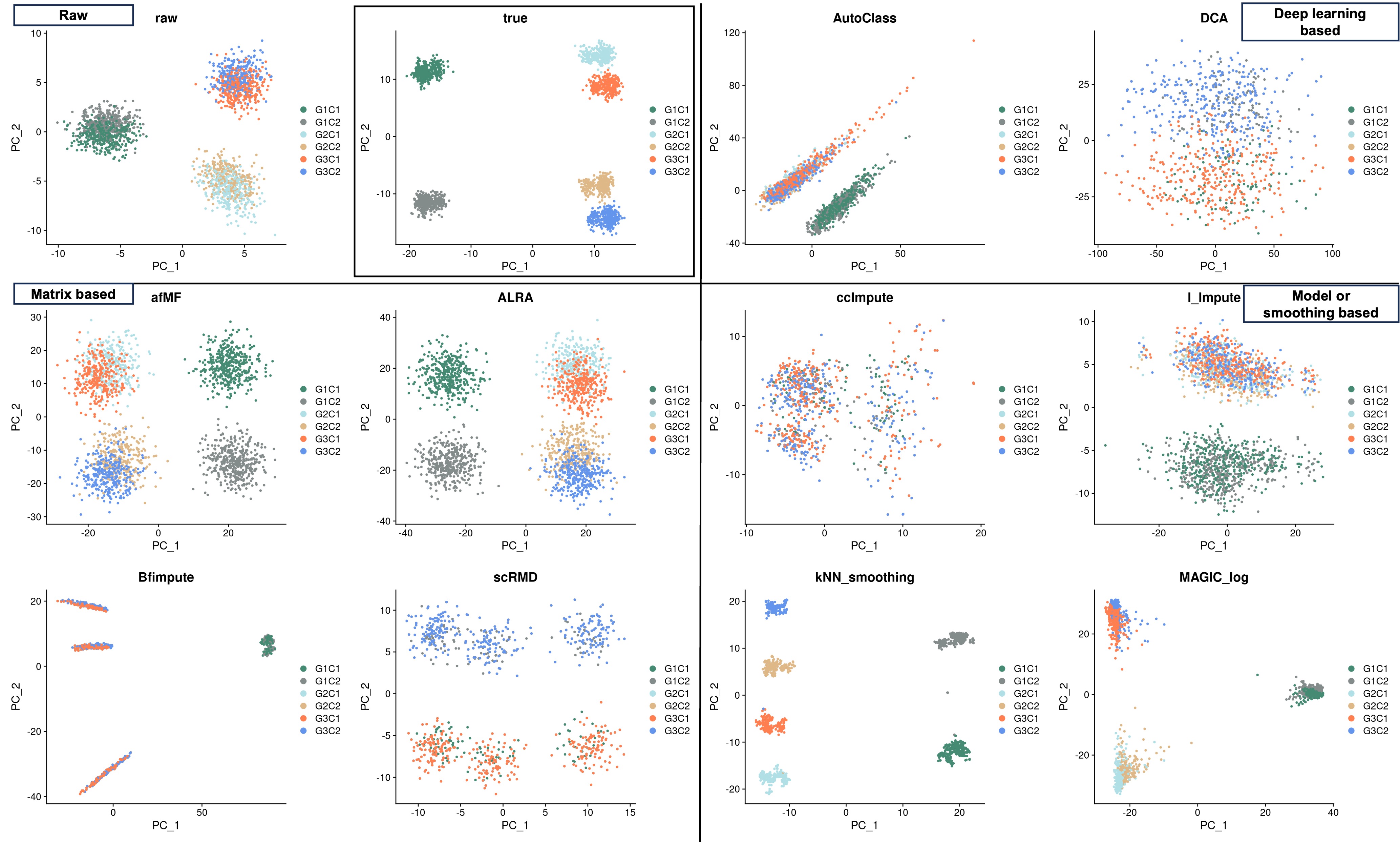
**

**Figure S5. Cell-Cell Correlation heatmaps in different imputations using CellBench-10X5CL**

**
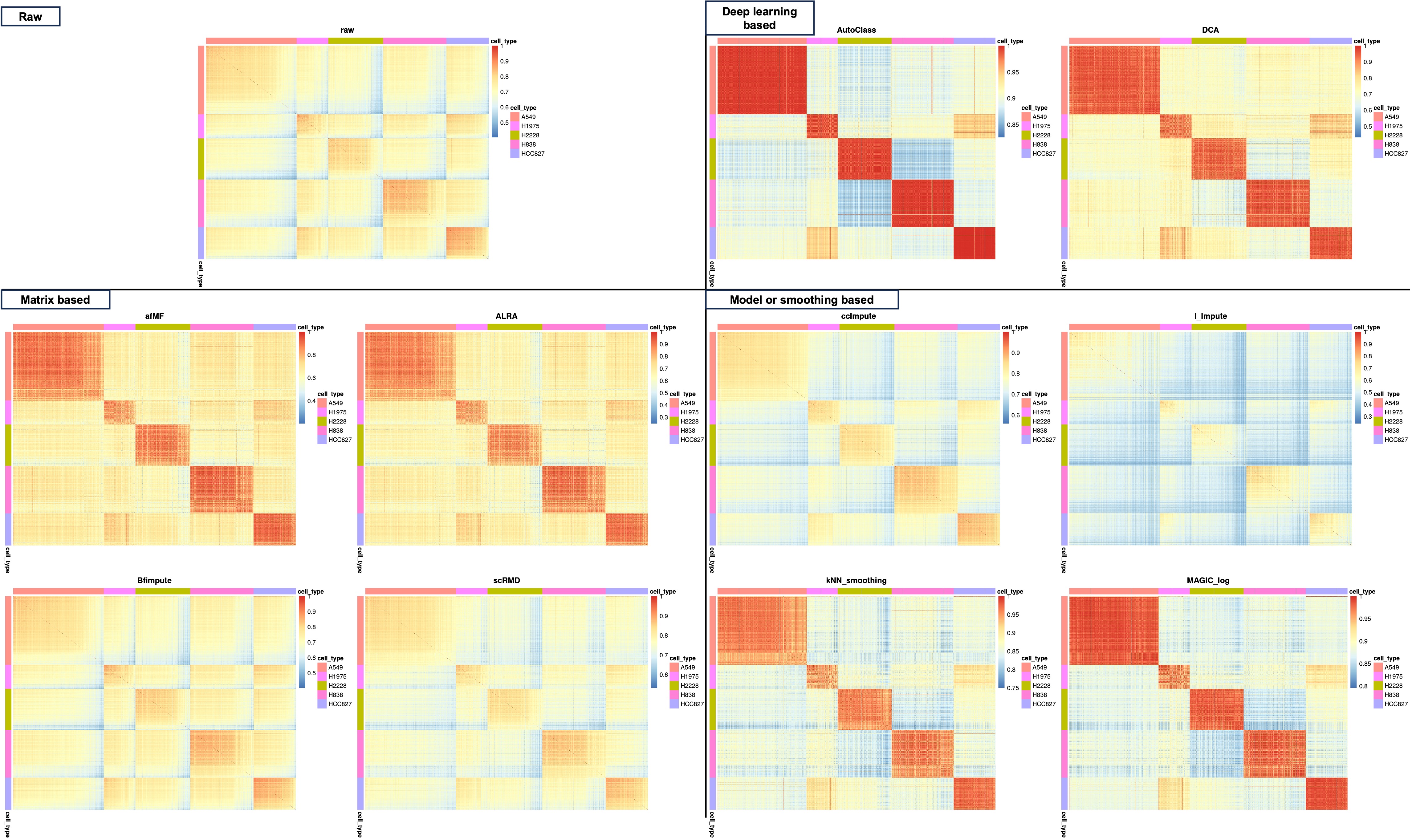
**
