# Supplementary material for: An in‐depth benchmark framework for evaluating single cell RNA‐seq dropout imputation methods and the development of an improved algorithm afMF
Source: Clin Transl Med. 2025 Mar 22;15(4):e70283. doi: 10.1002/ctm2.70283 (PMC11928879; doi:10.1002/ctm2.70283)

**Method S3. Differential expression analysis and GSEA**

*Differential Expression (DE) Analysis*

We evaluated the performance of different imputations methods on DE analysis using seven real scRNA-seq datasets with their matched bulk (GSE75748 (single cell and time-course), GSE81861, CellBench-10X5CL, GSE124872, EGAS00001003215, GSE79578 and GSE90047), where the matched bulk DE results was used as ‘gold standard’. Datasets were divided into three types: mixture cell types, purified cell types with two conditions, and time-course. The gene expression between pairwise cell types, conditions and time were compared respectively. In brief, bulk DE analysis was performed by limma-voom^1^. DE analysis for scRNA-seq was performed by Wilcoxon Rank Sum test, MAST^2^ and pseudobulk-limma-trend^3^. The popular pseudobulk DE analysis aggregates the information from same cell types and individuals and has been demonstrated to be the preferred method for DE analysis^3,4^. Note that the pseudobulk DE analysis was only performed on datasets with multiple individuals (GSE124872 and EGAS00001003215). Results were then ranked by sign (i.e., the sign of the fold change) -log_10_(P-value) and the absolute values of fold changes (FC). Next, the Spearman correlation coefficients were calculated for both gene ranks and logFC between the bulk results and the single cell results. All genes and the top 1000 DEGs (in bulk) were compared. Additionally, the significances of the top 500 DEGs in bulk data were compared among different methods. The false positive rates, defined as the percentages of tailed 10%, 20%, 25%, 33%, and 50% percentiles genes in bulk presented in the top 500 DEGs in single cell results, were also compared. These metric values were subtracted by the results of the unimputed log-normalized data. Extreme values were limited to a cutoff value for better visualization. All the analyses were performed with the default parameters unless other specified. Note that in time-course data the median values of the three datasets were used for bar plots since only three data points presented.

*GSEA*

The GSEA was performed for each DE result using the R package ClusterProfiler^5^, based on either sign -log_10_(P-value) or logFC (i.e., as input). The Gene Ontology (GO) terms were used as the gene sets. The bulk GSEA results were used as the gold standard. The Spearman correlation coefficients were calculated for ‘sign (Enrichment Score) $\times$ -log_10_(P-value)’ between the bulk GSEA results and the single cell GSEA results. All terms and ‘P<0.05’ terms were compared. The metric values were subtracted by the results of the unimputed log-normalized data. Extreme values were limited to a cutoff value for better visualization. All the analyses were performed with the default parameters unless other specified. Note that in time-course data the median values of the three datasets were used for bar plots since only three data points presented.

**Note S3.**

DE analysis is the essential part of downstream applications. For scRNA-seq data, DE can be analyzed by one of these methods: (1) MAST; (2) Wilcoxon rank sum test; or (3) pseudobulk analysis. Our results showed that performance of imputation in DE analysis was dependent on the methods used. Enhancement was found in (1) MAST and (2) Wilcoxon rank sum test, while setback was found when using imputed data with (3) pseudobulk analysis for DE analysis and GSEA.

Majority of the previous evaluations used p-value cutoffs, overlap rates, or simulated data that may have drawbacks: heavily affected by sample size, did not make full use of gene rank information, or unable to reflect the real data distribution. One example is that an algorithm may increase overlap DEGs, but the top rankings may be totally different from ground truth, which is not desired. Therefore, we conducted the evaluation based on gene rank information. Using MAST and Wilcoxon rank sum test, higher p-value-based rank concordance between bulk and afMF-imputed DE results were observed for all types of data (i.e., mixture / purified / time-course) with statistical significance (**Figure 2A upper** and **Figure S6-7 upper**). These conclusions still held when limiting the genes to only the top 1000 DEGs (determined in bulk data) (**Figure S8**). The top 500 DEGs showed greater statistical significance in afMF, ALRA, MAGIC and AutoClass (**Figure 2A middle** and **Figure S6-7 middle**). Next, false positive rates were calculated based on ranks in single cell and bulk (see Methods) and only afMF and I_Impute showed generally lower false positive rates in all types of data (**Figure 2A bottom** and **Figure S6-7 bottom** and **Figure S9**). Additionally, higher logFC Spearman correlations were observed between bulk and afMF, MAGIC, and DCA imputed results for all types of data (**Figure S10**).

Imputation is incompatible with pseudobulk DE analysis using limma-trend. Nearly all the imputation algorithms performed worse than no imputation in pseudobulk DE analysis (**Figure S11**). Only the logFCs of kNN-smoothing and DCA had higher correlations with bulk results. Though most algorithms increased the significance of the top 500 DEGs, they had higher false positive rates as well. For example, 6 out of 10 imputation algorithms significantly increase the number of false positive DEGs in pseudobulk analysis. These results suggested that pseudobulk processing may serve as a smoothing step and thus heavily decreased the influence of dropouts in DE analysis.

GSEA is an extension use of DE results to study the enrichments of DEGs in specific biological processes or molecular functions. Whether imputation can improve GSEA was not studied yet. Our results showed that performance with GSEA followed that of DE analysis and it depended on the DE statistics used. Using either MAST DE sign -log_10_P or logFC as input, higher Spearman correlations between bulk GSEA and afMF-imputed GSEA results were observed for all types of data (**Figure 2B** and **Figure S12**). In contrast, MAGIC / MAGIC-log and AutoClass performed generally better as well but showed no improvement when using logFC or sign -log_10_P as input respectively in mixture data. These conclusions held when limiting to enrichment terms with P<0.05 (i.e., in bulk results) (**Figure S13**). When using Wilcoxon Rank Sum DE results, MAGIC/MAGIC-log, afMF and ALRA showed steady improvements (**Figure S14**). When using pseudobulk DE results, all imputations with sign -log_10_P as input showed no improvements, while kNN-smoothing, ALRA, I-Impute, DCA and afMF increased the correlations using logFC as input (**Figure S15**). These results suggested that imputation could improve the discoveries of gene programs under specific conditions.

**Reference**

1. Law CW, Chen Y, Shi W, Smyth GK. voom: Precision weights unlock linear model analysis tools for RNA-seq read counts. *Genome Biol*. 2014;15(2):R29. doi:10.1186/gb-2014-15-2-r29

2. Finak G, McDavid A, Yajima M, et al. MAST: a flexible statistical framework for assessing transcriptional changes and characterizing heterogeneity in single-cell RNA sequencing data. *Genome Biol*. 2015;16:278. doi:10.1186/s13059-015-0844-5

3. Squair JW, Gautier M, Kathe C, et al. Confronting false discoveries in single-cell differential expression. *Nat Commun*. 2021;12(1):5692. doi:10.1038/s41467-021-25960-2

4. Crowell HL, Soneson C, Germain PL, et al. muscat detects subpopulation-specific state transitions from multi-sample multi-condition single-cell transcriptomics data. *Nat Commun*. 2020;11(1):6077. doi:10.1038/s41467-020-19894-4

5. Yu G, Wang LG, Han Y, He QY. clusterProfiler: an R package for comparing biological themes among gene clusters. *OMICS*. 2012;16(5):284-287. doi:10.1089/omi.2011.0118

**Figure S6. Performance of imputations in differential expression analysis (single cell level with MAST) (purified cell type and time course data)**

**
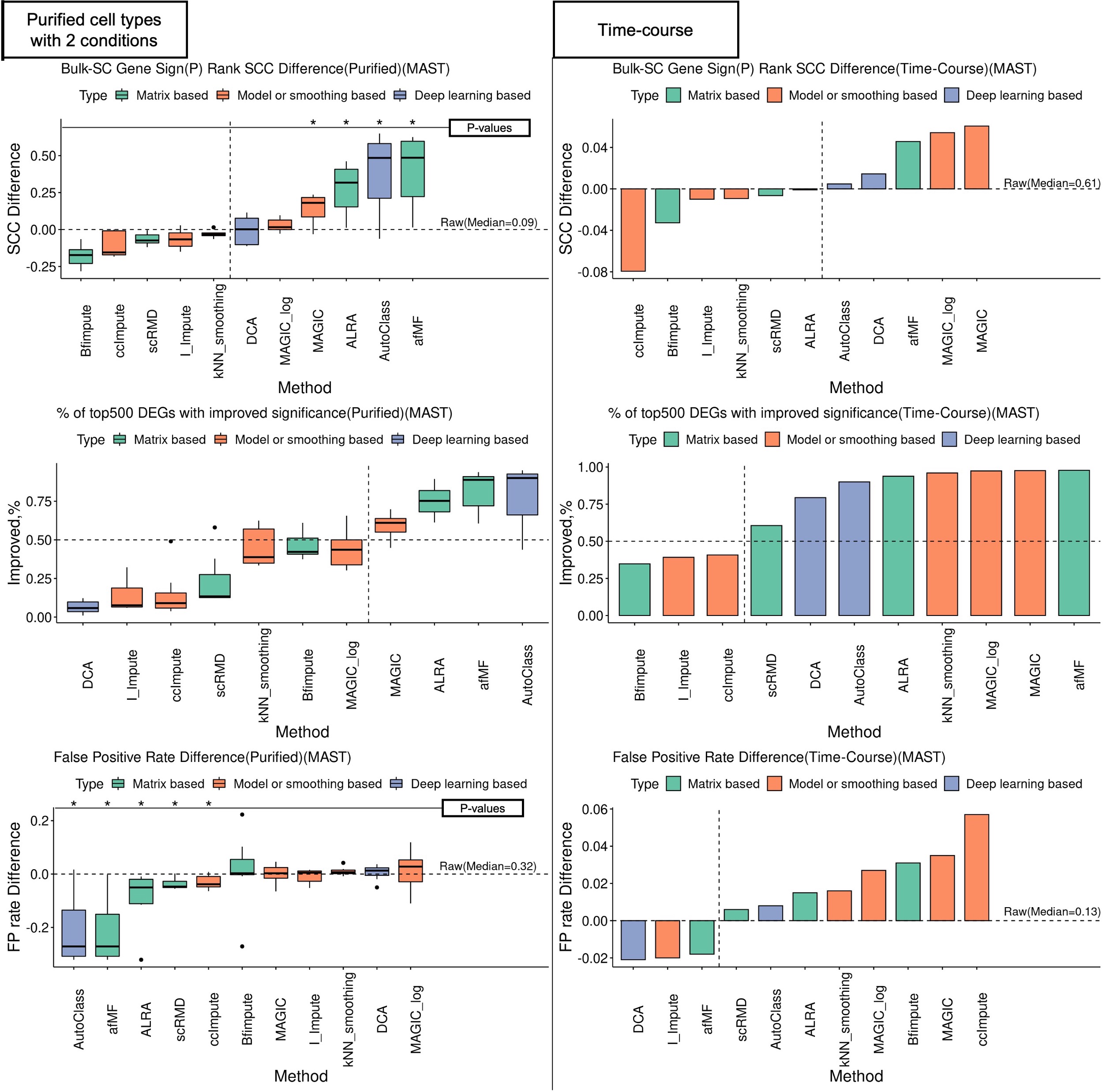
**

**Figure S7. Performance of imputations on Differential Expression Analysis with Wilcox Rank Sum test**


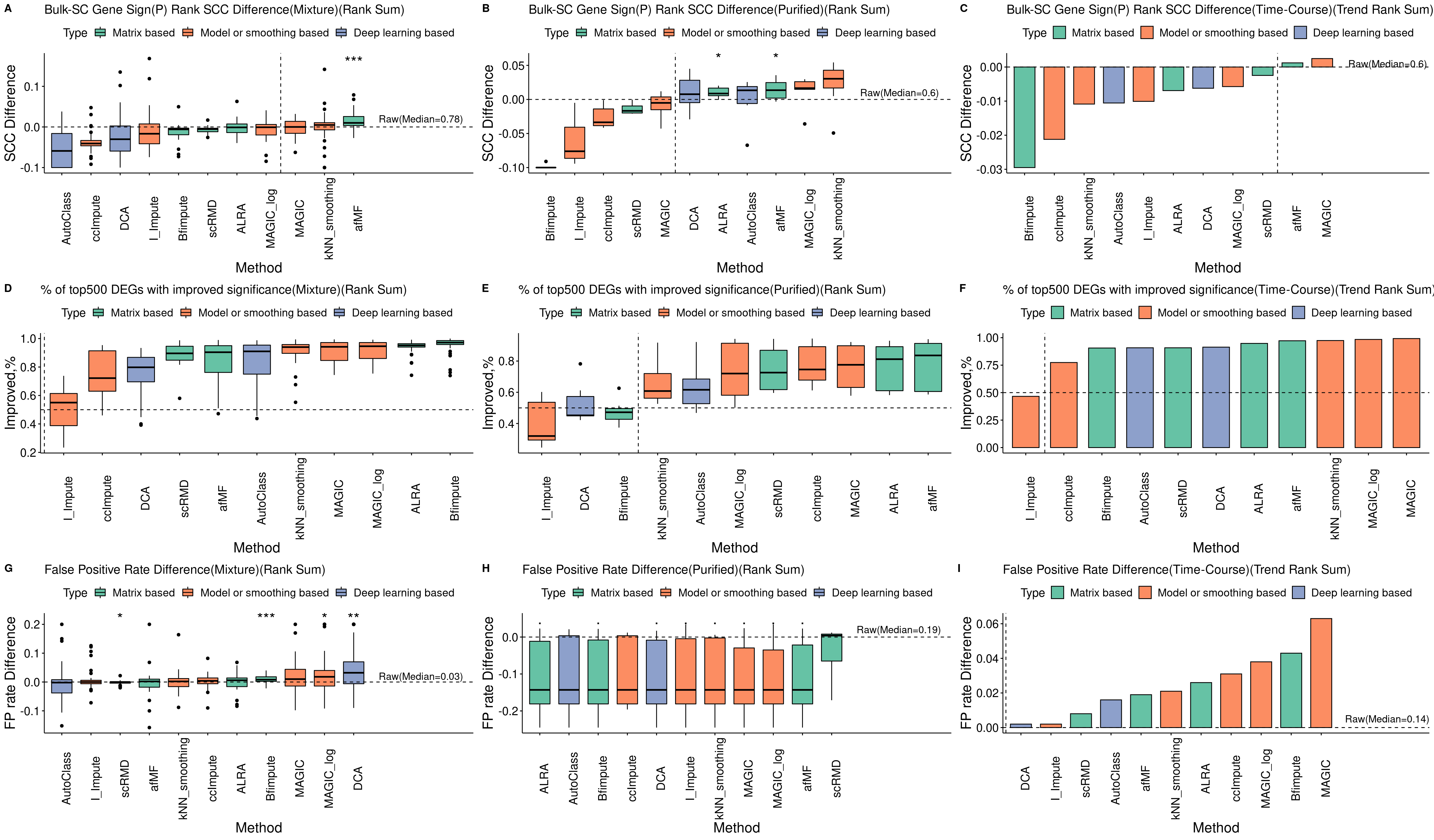


**Figure S8. Performance of imputations on Differential Expression Analysis with MAST & Wilcox Rank Sum test: SCC for Top 1000 DEGs**


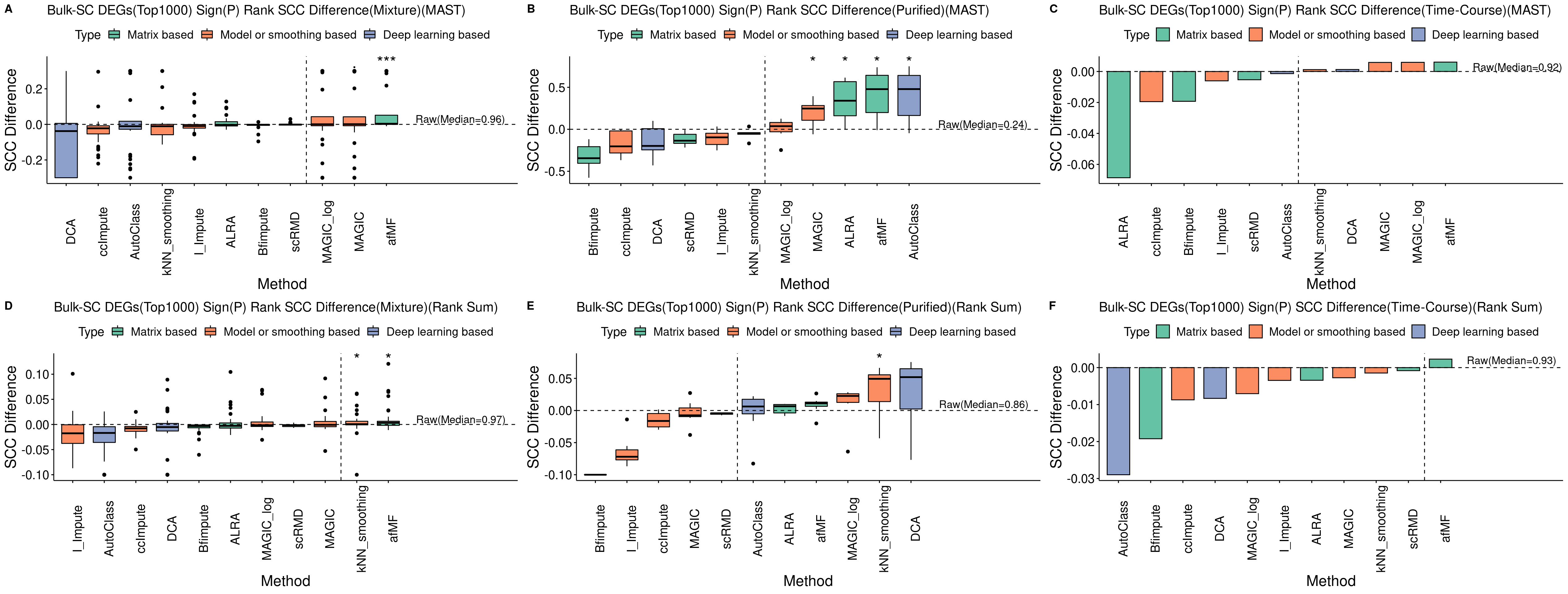


**Figure S9. Performance of imputations on Differential Expression Analysis with MAST & Wilcox Rank Sum test: false positive rates (percentile tail-ranked genes in bulk presented in top 500 single cell DEGs)**

**
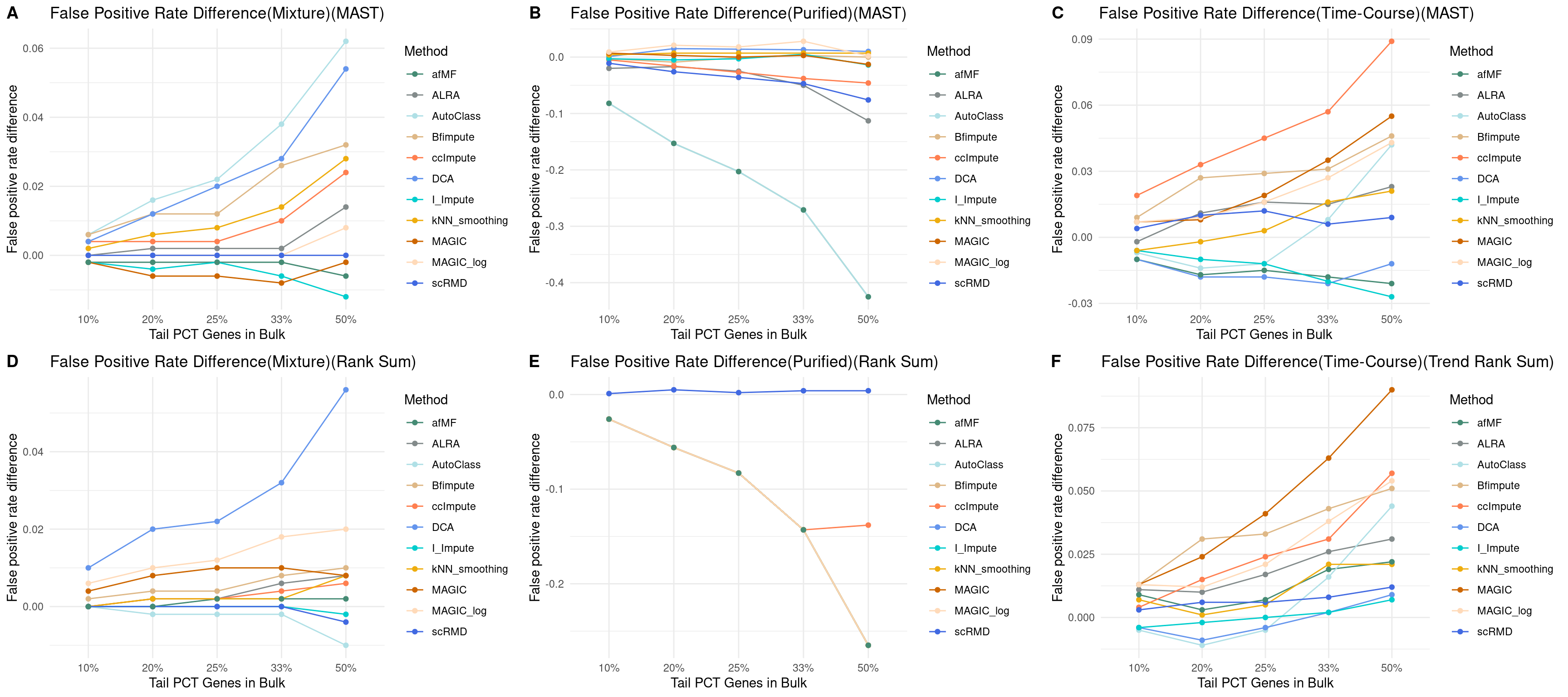
**

**Figure S10. Performance of imputations on Differential Expression Analysis with MAST: correlations of logFC**


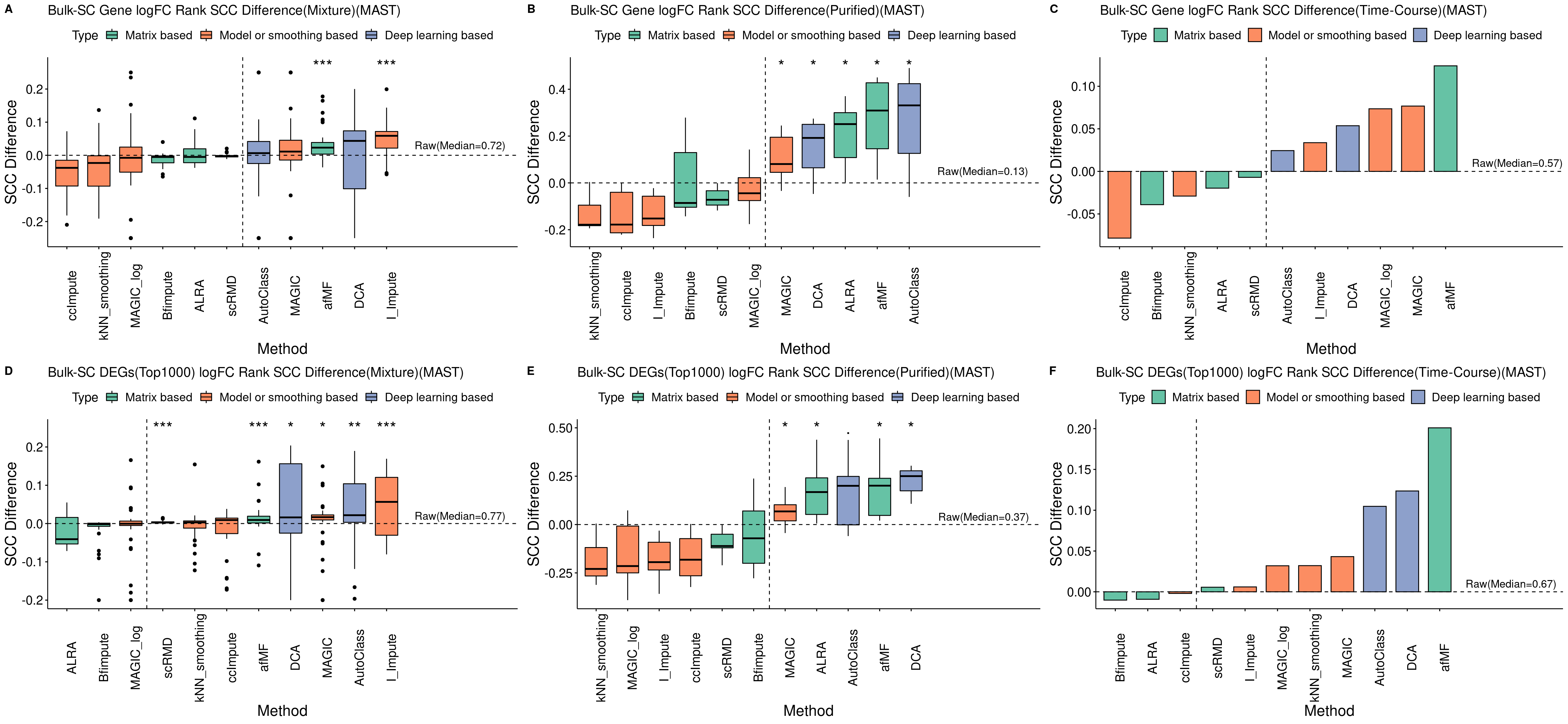


**Figure S11. Performance of imputations on Differential Expression Analysis with pseudobulk-limma-trend**


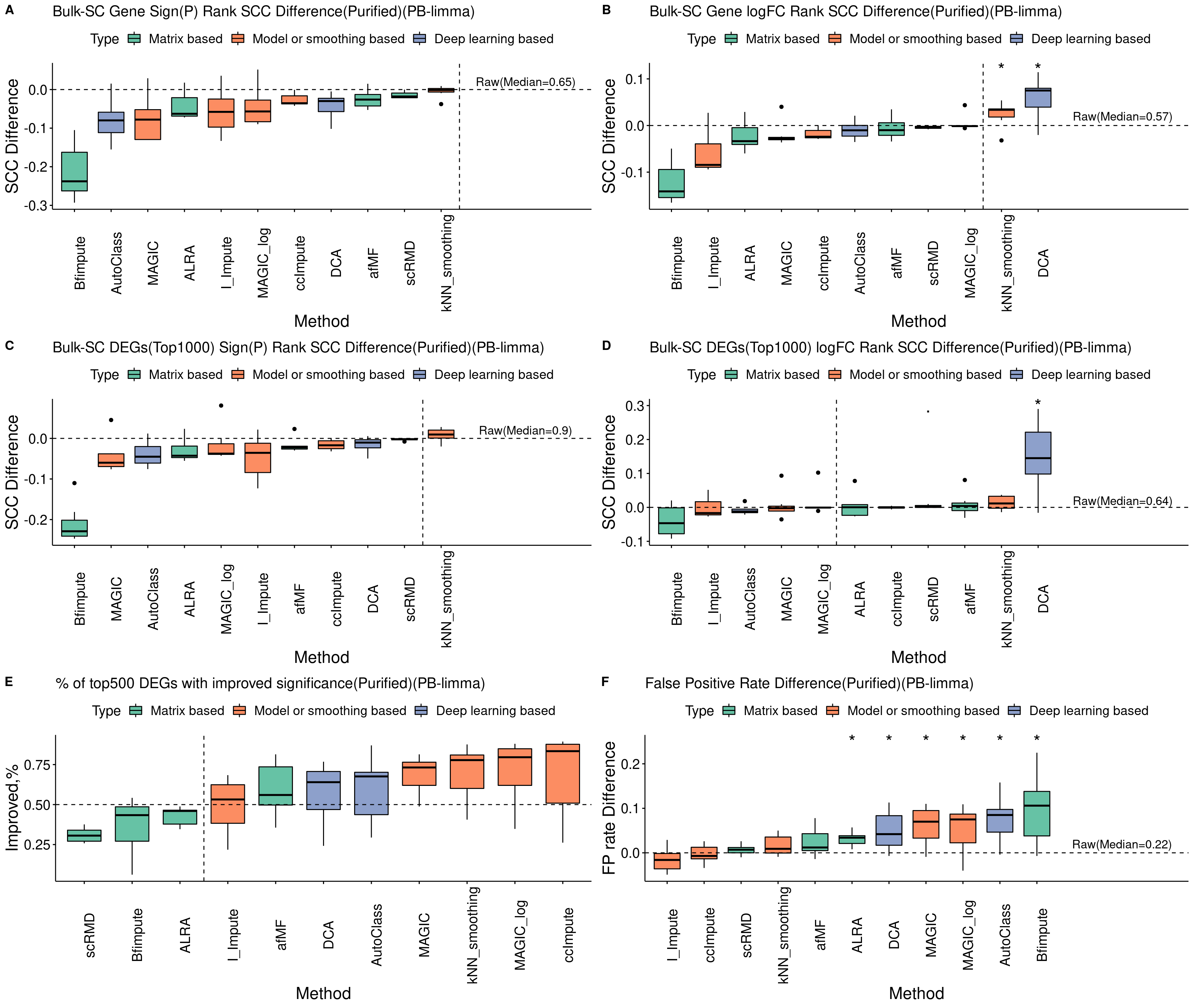


**Figure S12. Performance of imputations in GSEA (purified cell type and time course data)**

**
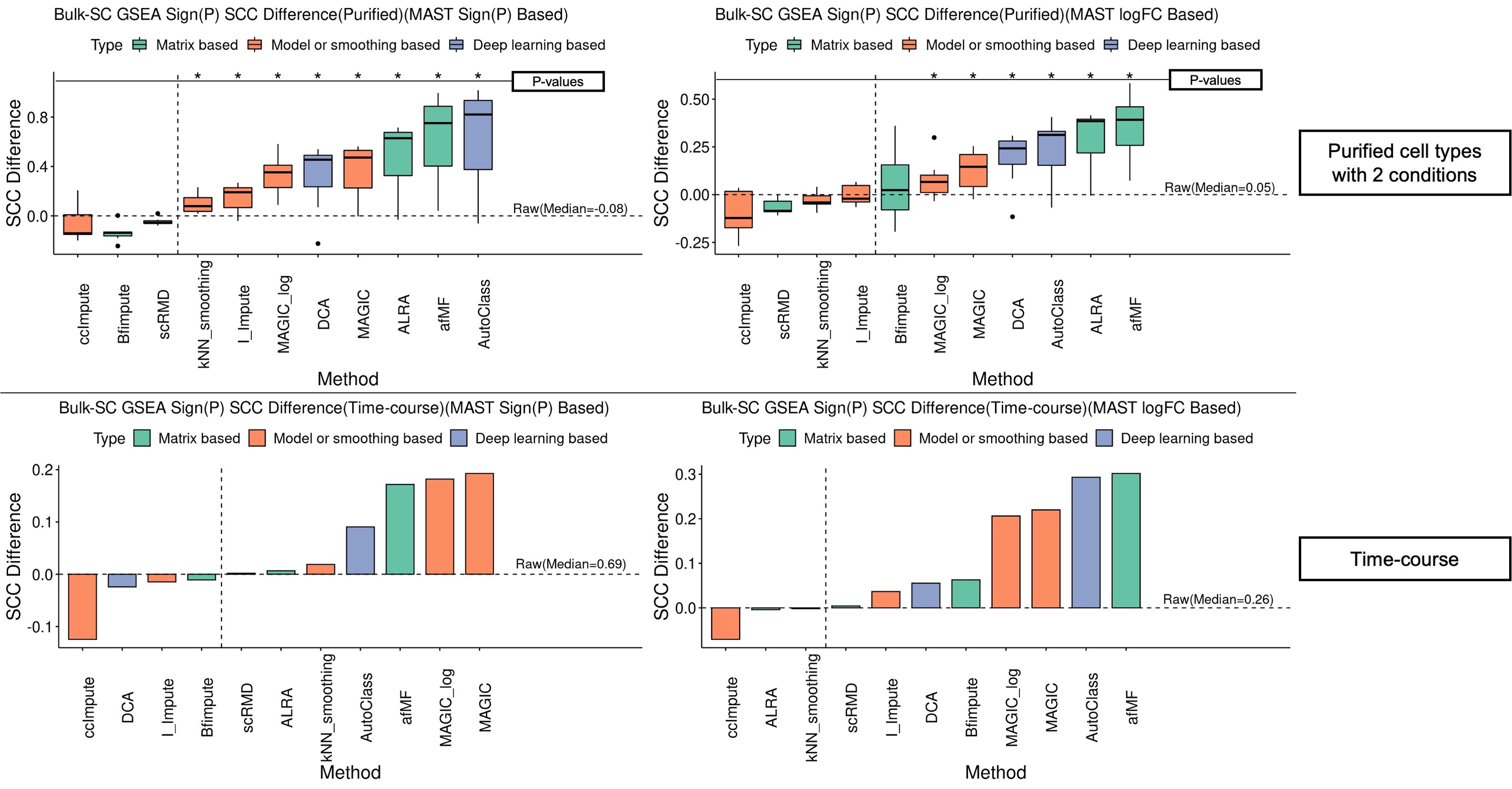
**

**Figure S13. Performance of imputations on MAST-based GSEA: correlations of P<0.05 GO terms (bulk)**


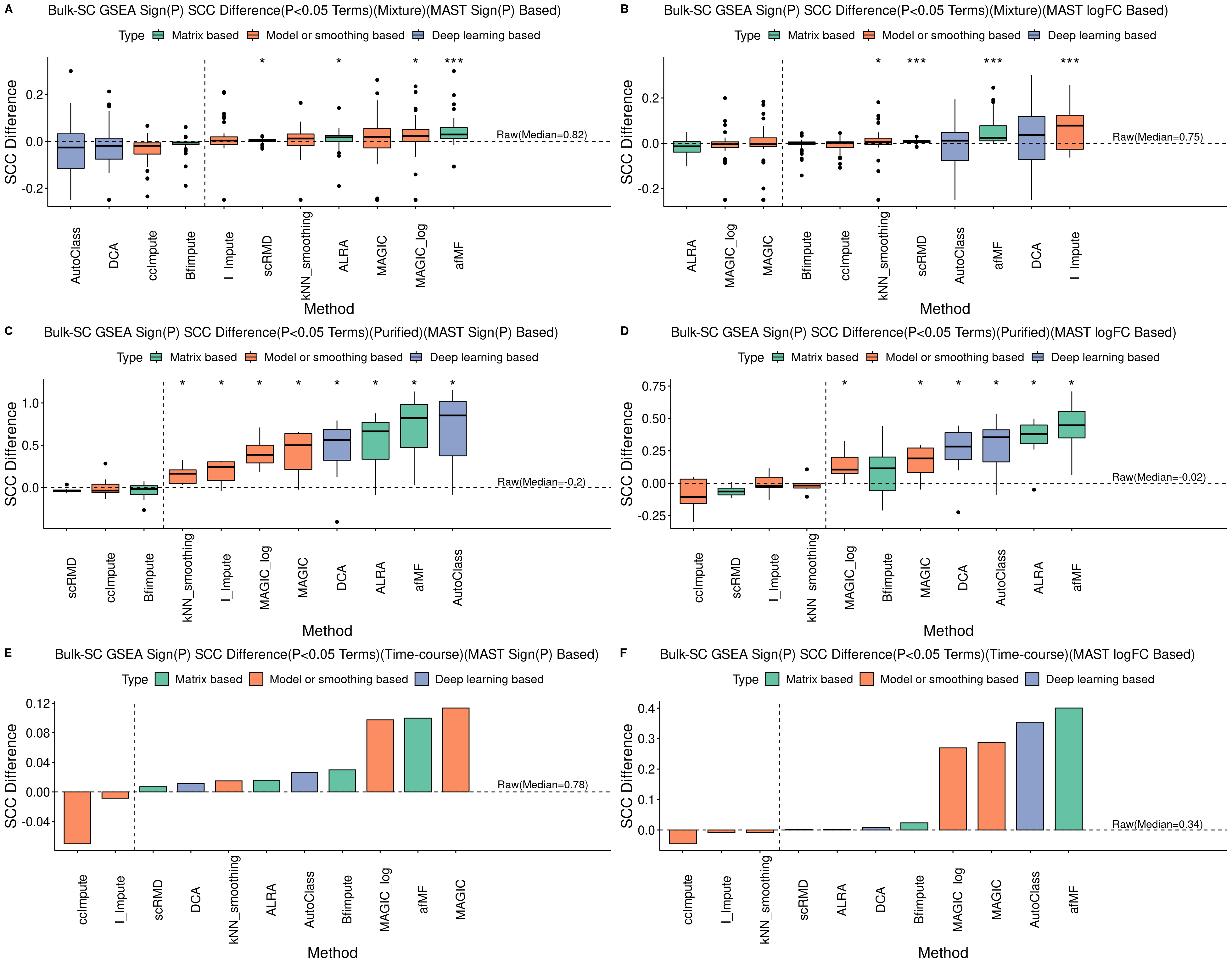


**Figure S14. Performance of imputations on Wilcox Rank Sum-based GSEA**


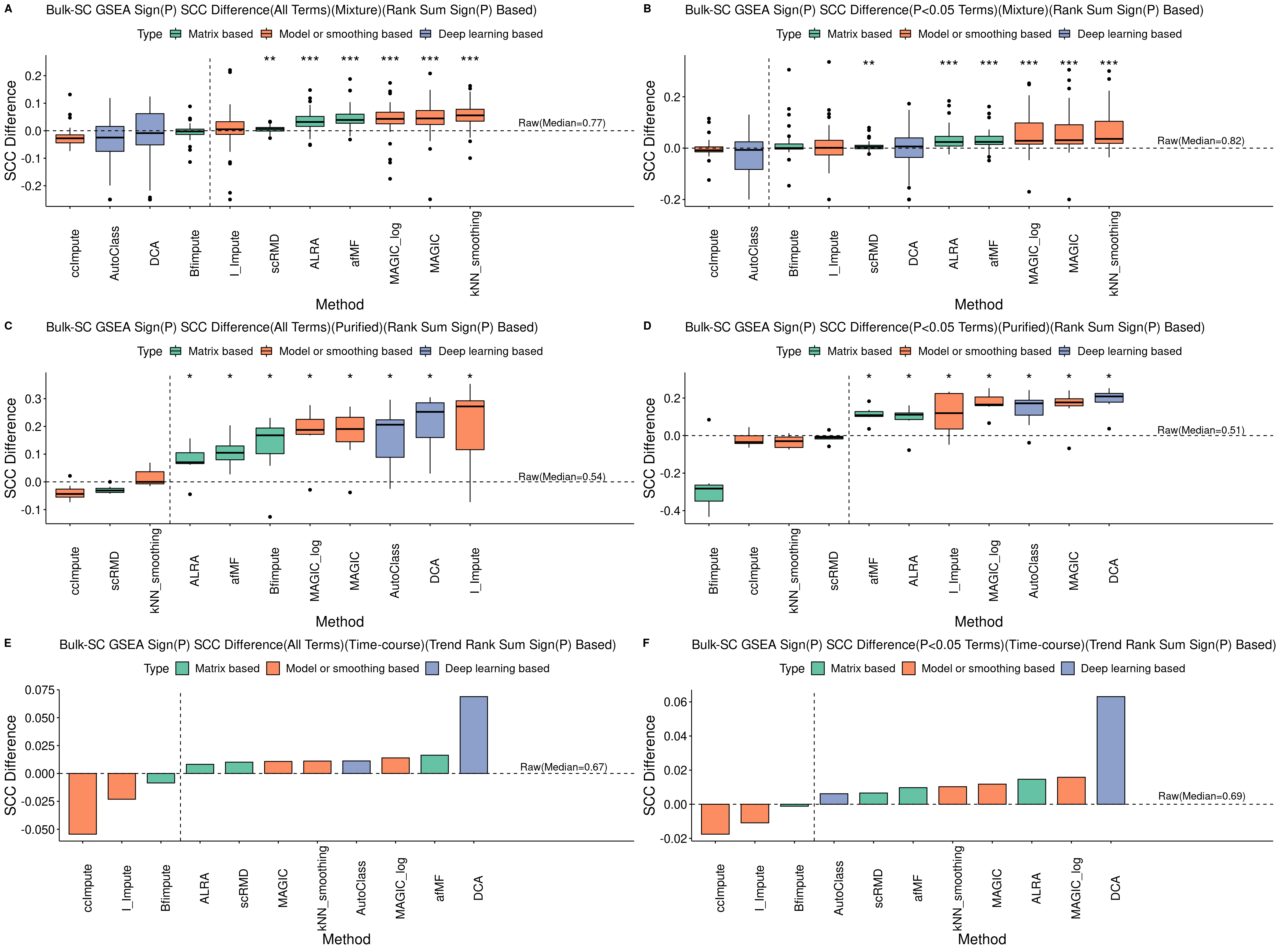


**Figure S15. Performance of imputations on pseudobulk-limma-trend-based GSEA**


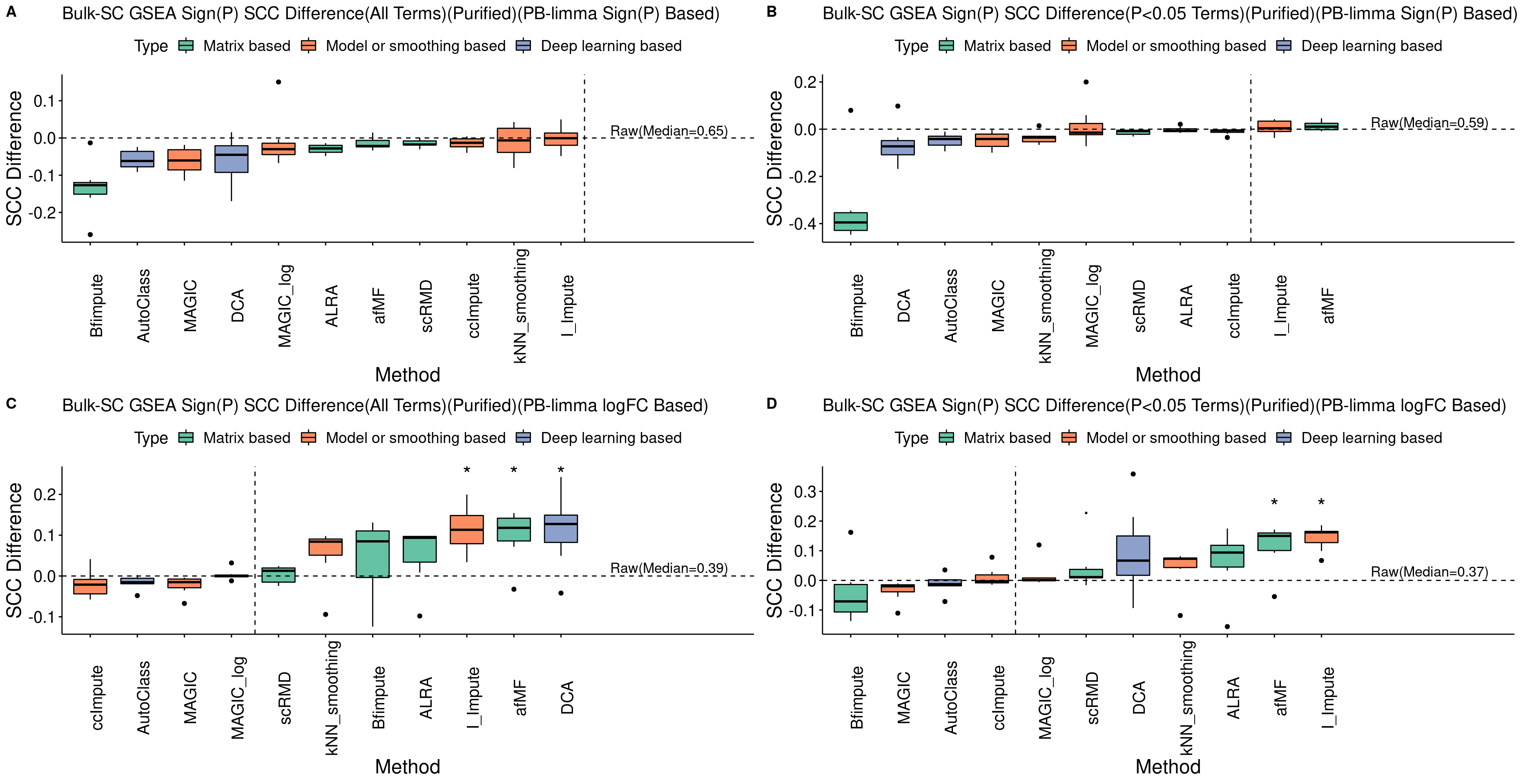

Supplement: Supplementary file 3 — Supporting Information [file CTM2-15-e70283-s009.docx]
