# Supplementary material for: An in‐depth benchmark framework for evaluating single cell RNA‐seq dropout imputation methods and the development of an improved algorithm afMF
Source: Clin Transl Med. 2025 Mar 22;15(4):e70283. doi: 10.1002/ctm2.70283 (PMC11928879; doi:10.1002/ctm2.70283)

**Method S4. Supporting analysis: SC-Bulk/Protein profiling similarities**

*Correlations between bulk RNA-seq profiles and single cell RNA-seq profiles*

In this part, we adopted Hou’s method^1^, made some revisions and extended to different data (GSE75748, GSE81861 and CellBench-10X5CL).

Specifically, Spearman correlation coefficients were calculated for each single cell profile and the bulk profile (within same group):

$${Cor}_{spearman}({Single Cell}_{group1},{Bulk}_{group1})$$

We noticed some methods can equally improve the correlations of different groups. Therefore, to reduce this influence we subtracted the results by the max different-group correlation coefficients:

$${Cor}_{spearman}\left( {Single Cell}_{group1},{Bulk}_{group1} \right)-max({Cor}_{spearman}\left( {Single Cell}_{group1},{Bulk}_{group2 or 3 or 4} \right))$$

The median value of each result was calculated for comparison.

We performed the same analysis on pseudobulk profiles. Spearman correlation coefficients were calculated for each pseudobulk profile (i.e., median-aggregate of the same group) and the bulk profile (same group):

$${Cor}_{spearman}({Pseudobulk}_{group1},{Bulk}_{group1})$$

Then subtracted the results by the max different-group correlation coefficients:

$${Cor}_{spearman}\left( {Pseudobulk}_{group1},{Bulk}_{group1} \right)-max({Cor}_{spearman}\left( {Pseudobulk}_{group1},{Bulk}_{group2 or 3 or 4} \right))$$

Next, we conducted pair-wise calculations of log fold changes between different groups in both pseudobulk profiles and bulk profiles and performed the Spearman correlation analysis:

$${Cor}_{spearman}({Pseudobulk:logFC}_{group1-2},{Bulk:logFC}_{group1-2})$$

The metric values were subtracted by the results of the unimputed log-normalized data. Extreme values were limited to a cutoff value for better visualization.

*Correlations between surface protein profiles and mRNA profiles in single cell*

We used the data GSE100866 to study the impact of imputation on the correlations between measured mRNA and surface protein^2^. Six marker genes in two tissues (CD4, CD2, CD19, CD14, CD34, CCR7 in PBMC and CBMC) with corresponding surface protein measurement were selected and compared. Spearman correlation coefficients were calculated between the two measurements for each gene. The metric values were subtracted by the results of the unimputed log-normalized data.

**Note S4.**

Additional support of using imputation can be gathered from datasets with additional data after cell sorting or protein assay to validate transcriptome results. For one cell type, gene expression measured from bulk RNA-seq are more accurate than single cell RNA-seq. Bulk RNA-seq measures gene expression of groups of cells and thus better reflects the ground truth in some circumstances, i.e., when no need to consider cell-cell variability. Using three mixture datasets with matched bulk data, higher relative (same minus others) and absolute (same) Spearman correlations between same-cell-type single cell/pseudobulk (median-aggregated) and bulk profiling were observed for most algorithms with statistical significance, except for scRMD, Bfimpute and DCA (**Figure 2C upper** and **Figure S16A-D**). Correlations of the pairwise cell type logFC between pseudobulk and bulk were higher in most imputations as well, except for Bfimpute and ccImpute (**Figure 2C bottom left** and **Figure S16E**).

Though not all mRNA measured in scRNA-seq will translate to protein, some correlations between mRNA and protein are still expected. Using a CITE-seq dataset (GSE100866 PBMC and CBMC) with both mRNA and surface protein measurement, higher Spearman correlations between selected mRNA and surface protein were observed in ALRA, kNN-smoothing, AutoClass, afMF and DCA (**Figure 2C bottom right** and **Figure S16F**).

**Reference**

1. Hou W, Ji Z, Ji H, Hicks SC. A systematic evaluation of single-cell RNA-sequencing imputation methods. *Genome Biol*. 2020;21(1):218. doi:10.1186/s13059-020-02132-x

2. Linderman GC, Zhao J, Roulis M, et al. Zero-preserving imputation of single-cell RNA-seq data. *Nat Commun*. 2022;13(1):192. doi:10.1038/s41467-021-27729-z

**Figure S16. Performance of imputations in various supporting analysis and heatmaps showing each comparison**


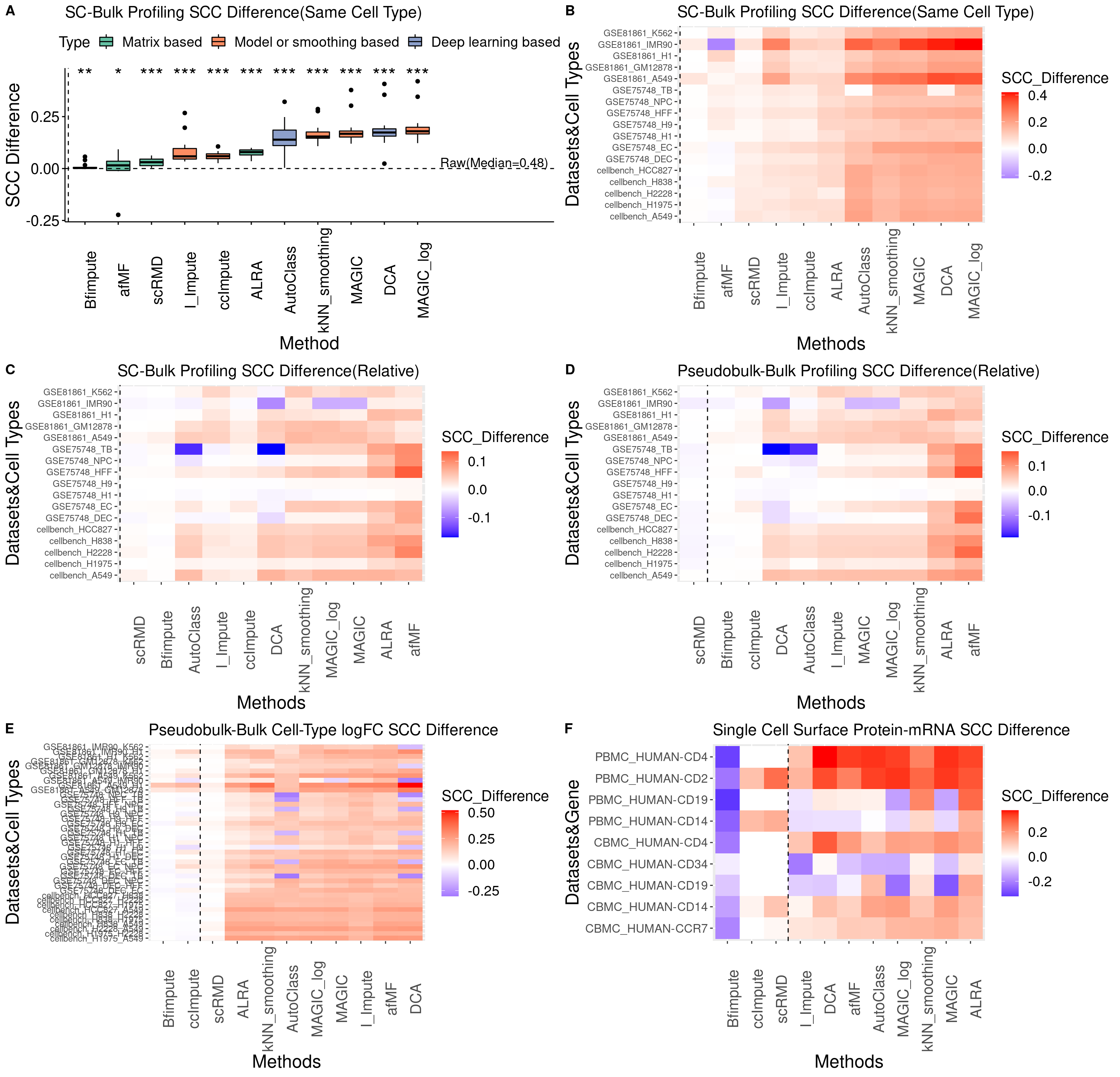

Supplement: Supplementary file 4 — Supporting Information [file CTM2-15-e70283-s008.docx]
