# Supplementary material for: An in‐depth benchmark framework for evaluating single cell RNA‐seq dropout imputation methods and the development of an improved algorithm afMF
Source: Clin Transl Med. 2025 Mar 22;15(4):e70283. doi: 10.1002/ctm2.70283 (PMC11928879; doi:10.1002/ctm2.70283)

**Method S5. Classification, biomarker prediction, and automatic cell type annotation.**

*Classification and Biomarker Predictive Performance*

Classifications for different cell types, conditions and time point in mixture, purified and time-course data were performed using Random Forest (RF) model from R RandomForest package. The top 10 DEGs for each comparison (i.e., one vs. others) in bulk data were used as input features. For each dataset, 70% samples were used as training set and the other 30% were for testing. The classification accuracy and RF predict probability for true label were compared.

The biomarker predictive performances of different methods were evaluated. Top 10 DEGs for pairwise cell types/conditions comparisons in bulk data were used as biomarkers for evaluation. The predictive performance was evaluated by calculating the area under the receiver operating characteristic curve (AUROC) for each of the selected genes in corresponding comparisons. The metric values were subtracted by the results of the unimputed log-normalized data. All the analyses were performed with the default parameters unless other specified.

*Automatic cell type annotation*

Two well-established R packages, SCINA^1^ and ScType^2^, were applied to evaluate the performance of different imputations on automatic cell type annotation. Two well-labeled data (GSE155673 and ROSMAP brain) were used. The cell-type-specific marker genes used as input were collected from Azimuth (https://azimuth.hubmapconsortium.org/) and PanglaoDB^3^. The annotation accuracy, F1 score, true-label-annotation probability and unknown rate were calculated and compared. Extreme values have been limited to a cutoff value for better visualization. All the analyses were performed with the default parameters unless other specified.

**Note S5.**

Classification would be useful when identifying cell subtypes or predicting cells with unknown labels. Using Random Forest model, we observed higher classification accuracy in nearly all the imputation methods for all types of data, except for DCA & kNN-smoothing (**Figure 2D** and **Figure S17**). Most imputation methods showed improvements in terms of correct cell type prediction probabilities except I-Impute and Bfimpute (**Figure 2D middle** and **Figure S18A-B**).

Cell-type-specific marker genes can be used to identify different cell types. Using Area Under Curve (AUC), higher AUC values for biomarker genes to discriminate cell types/conditions in mixture/purified data were discovered for MAGIC/MAGIC-log, AutoClass, kNN-smoothing, afMF, ALRA and DCA (**Figure 2D right** and **Figure S18C-F**). We also analyzed the extent of potential false positives and the non-marker genes were selected (based on bulk DE). Much higher percentage of non-markers with AUC>0.9 were discovered in AutoClass, MAGIC/MAGIC-log and DCA for both mixture and purified data (**Figure S18D** and **F**). Therefore, only kNN-smoothing, afMF, and ALRA enhanced the detection of cell type marker genes without heavily increasing the false positive rate, indicating that they are suitable for cell-type-specific marker identification.

Cell type annotation^4^ is the key step in scRNA-seq analysis. The use of imputation for cell type annotation may be underestimated. Since it usually takes much time and effort to do manual annotation, researchers have developed automatic tools to easily annotate cell types, such as SingleR^5^, scmap^6^ and Azimuth that require reference data, or other tools only require cell type marker genes as input. As imputation showed some advantages in classifications and biomarker predictions, we further investigated whether imputation was compatible with automatic cell type annotations. Using two well-established packages SCINA^1^ and ScType^2^, higher annotation accuracy, F1 scores and true prediction probabilities were observed for nearly all the selected imputation methods (**Figure 2E** and **Figure S19**), except for DCA that performed worse in some cell types. As a result, all the selected imputation methods resulted in lower unknown rates (**Figure 2E right**).

**Reference**

1. Zhang Z, Luo D, Zhong X, et al. SCINA: A Semi-Supervised Subtyping Algorithm of Single Cells and Bulk Samples. *Genes (Basel)*. 2019;10(7):531. doi:10.3390/genes10070531

2. Ianevski A, Giri AK, Aittokallio T. Fully-automated and ultra-fast cell-type identification using specific marker combinations from single-cell transcriptomic data. *Nat Commun*. 2022;13(1):1246. doi:10.1038/s41467-022-28803-w

3. Franzén O, Gan LM, Björkegren JLM. PanglaoDB: a web server for exploration of mouse and human single-cell RNA sequencing data. *Database (Oxford)*. 2019;2019:baz046. doi:10.1093/database/baz046

4. Clarke ZA, Andrews TS, Atif J, et al. Tutorial: guidelines for annotating single-cell transcriptomic maps using automated and manual methods. *Nat Protoc*. 2021;16(6):2749-2764. doi:10.1038/s41596-021-00534-0

5. Aran D, Looney AP, Liu L, et al. Reference-based analysis of lung single-cell sequencing reveals a transitional profibrotic macrophage. *Nat Immunol*. 2019;20(2):163-172. doi:10.1038/s41590-018-0276-y

6. Franchini M, Pellecchia S, Viscido G, Gambardella G. Single-cell gene set enrichment analysis and transfer learning for functional annotation of scRNA-seq data. *NAR Genom Bioinform*. 2023;5(1):lqad024. doi:10.1093/nargab/lqad024

**Figure S17. Performance of imputations in Classification and Biomarker Prediction (purified cell type and time course data)**

**
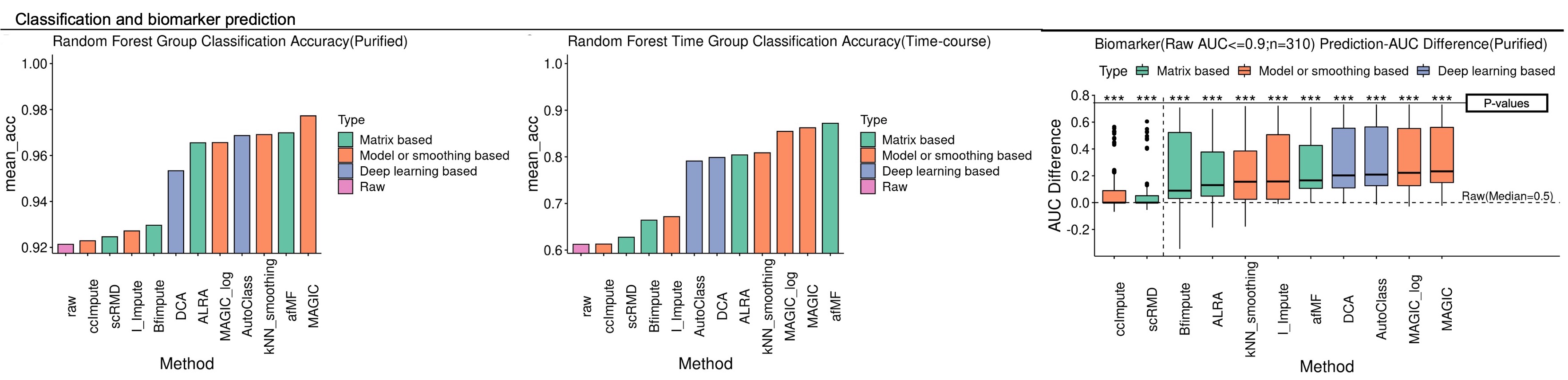
**

**Figure S18. Performance of imputations on Classification and Biomarker Predictions (supplementary results)**


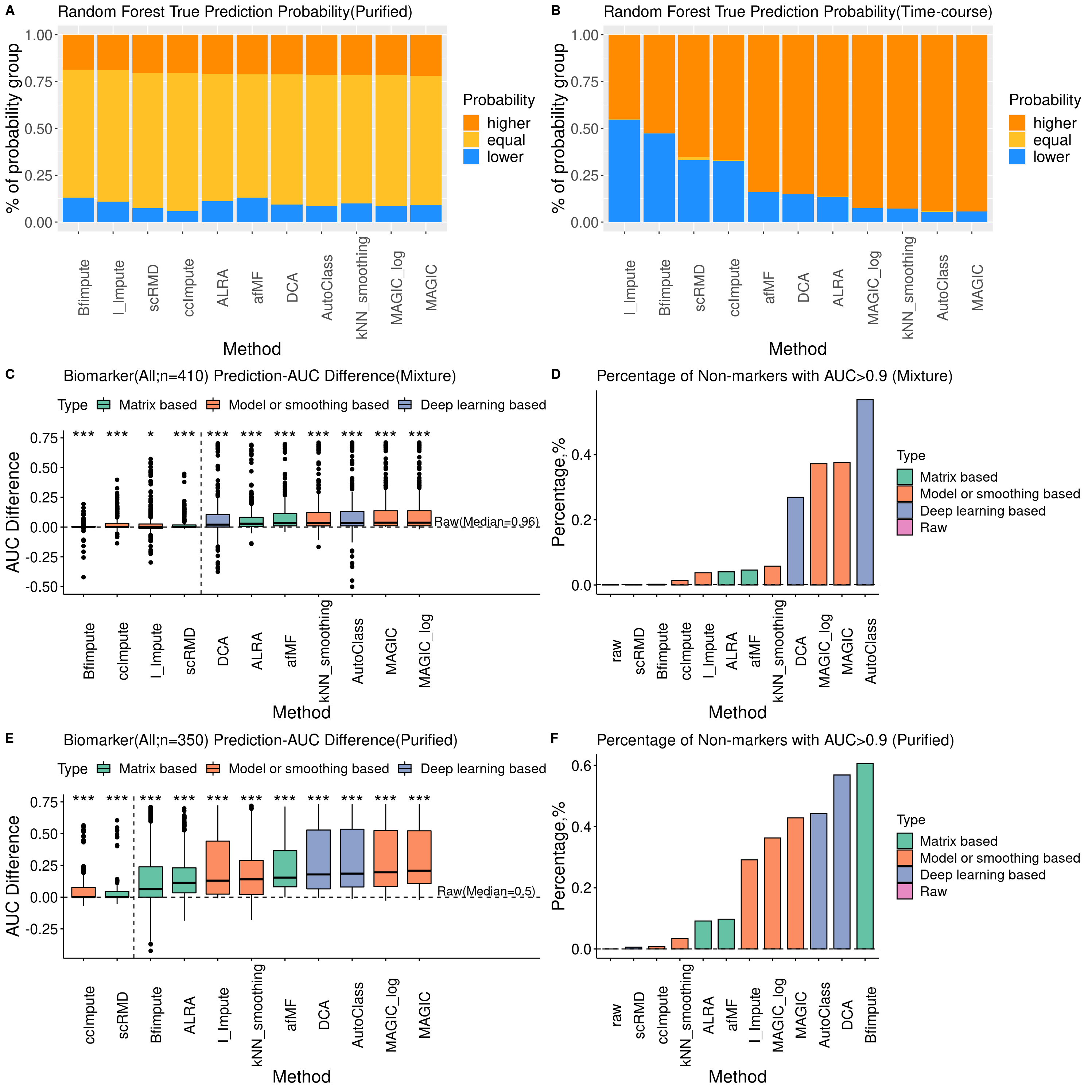


**Figure S19. Performance of imputations on Automatic Cell Type Annotation: SCINA and scType**


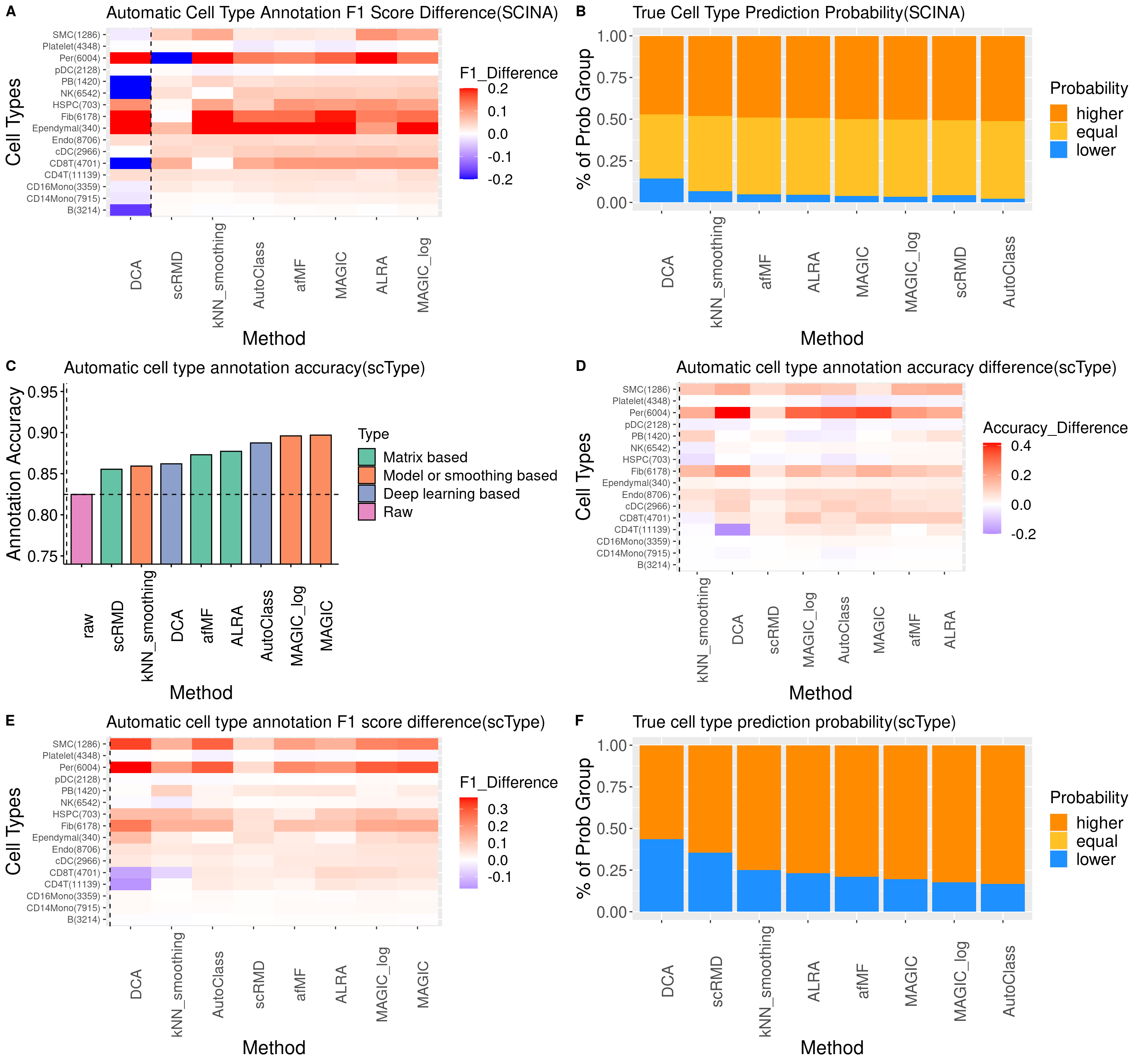

Supplement: Supplementary file 5 — Supporting Information [file CTM2-15-e70283-s004.docx]
