# Supplementary material for: An in‐depth benchmark framework for evaluating single cell RNA‐seq dropout imputation methods and the development of an improved algorithm afMF
Source: Clin Transl Med. 2025 Mar 22;15(4):e70283. doi: 10.1002/ctm2.70283 (PMC11928879; doi:10.1002/ctm2.70283)

**Method S6. Dimension reduction, clustering, and cell cycle dynamics**

*Dimension reduction, clustering, and cell cycle dynamics*

To quantitatively evaluate the clustering performance of different methods, we adopted four evaluation metrics, Entropy of accuracy ($H_{acc}$), Entropy of purity ($H_{pur}$), Adjusted Rand Index (ARI) and Normalized mutual information (NMI) as previously suggested^1,2^ using K-means Clustering and Louvain Clustering algorithms. Three simply labelled (i.e., with ground truth cell type label) datasets GSE75748, GSE81861, and CellBench 10X 5CL were used. Note that we did not include datasets with complicated design (i.e., with multiple conditions, tissues, cell types, batches, diseases, individuals, etc.) as there will be multiple labels for testing. In brief, the number of clusters was set to the number of known cell type labels in each dataset. The Seurat FindVariableFeatures() function with ‘vst’ methods were used to select high variable genes (top 3000 genes), followed by running PCA. The top 10 PCs were used for the two clustering algorithms. The detailed implementation of the algorithms was described previously^1^.

$H_{acc}$ evaluates the difference of the true groups within each predicted cluster, defined as:

$$H_{acc}=-\frac{\sum_{i=1}^{M} \sum_{j=1}^{N_{i}} p_{i}(x_{j})log(p_{i}(x_{j}))}{M}$$

$$0\leq H_{acc}\leq log(M)$$

where $M$ is the number of predicted clusters; $N_{i}$ is the number of true groups in the $i^{th}$ predicted cluster; $x_{j}$are cells in the $j^{th}$ true group; and $p_{i}(x_{j})$ are the proportions of cells in the $j^{th}$ true group relative to the total number of cells in the $i^{th}$ predicted cluster.

$H_{pur}$ evaluates the difference of the predicted clusters within each true group, defined as:

$$H_{pur}=-\frac{\sum_{i=1}^{N} \sum_{j=1}^{M_{i}} p_{i}(x_{j})log(p_{i}(x_{j}))}{N}$$

$$0\leq H_{pur}\leq log(N)$$

where $N$ is the number of true group; $M_{i}$ is the number of predicted clusters in the $i^{th}$ true group; $x_{j}$are cells in the $j^{th}$ predicted cluster; and $p_{i}(x_{j})$ are the proportions of cells in the $j^{th}$ predicted cluster relative to the total number of cells in the $i^{th}$ true group. Smaller $H_{acc}$ and $H_{pur}$ indicates better clustering performance^1^.

Adjusted Rand index (ARI) that evaluates the similarities between two data distributions was calculated using adjustedRandIndex() function in mclust package^3^, defined as:

$$ARI=\frac{\sum_{ij} \binom{n_{ij}}{2}-\left[ \sum_{i} \binom{a_{i}}{2}\sum_{j} \binom{b_{j}}{2} \right]/\binom{n}{2}}{\frac{1}{2}\left[ \sum_{i} \binom{a_{i}}{2}+\sum_{j} \binom{b_{j}}{2} \right]-\left[ \sum_{i} \binom{a_{i}}{2}\sum_{j} \binom{b_{j}}{2} \right]/\binom{n}{2}}$$

$$n_{ij}=\sum_{k,g} I(u_{k}=i)I(v_{g}=j)$$

$$a_{i}=\sum_{k} I(u_{k}=i)$$

$$b_{j}=\sum_{g} I(v_{g}=j)$$

$$I\left( x=y \right)=\left\{ \begin{aligned} 1, x=y \\ 0,otherwise \end{aligned} \right.$$

Where $i$ and $j$ enumerate the $k$ clusters; ${\{u_{i}\}}_{i}^{m}$ is the predicted cluster label; ${\{v_{j}\}}_{j}^{m}$ is the true group label.

Normalized mutual information (NMI) that measures the correlation between two random variables^2^ is calculated using NMI() function from aricode package, defined as:

$$NMI=2\frac{I(G,\hat{G})}{H\left( G \right)+H(\hat{G})}$$

$$I\left( G,\hat{G} \right)=\sum_{a\in G,b\in\hat{G}} p\left( a,b \right)log\frac{p(a,b)}{p\left( a \right)p(b)}$$

$$H\left( G \right)=\sum_{a\in G} p\left( a \right)logp(a)$$

Where $G$ is the true group, $\hat{G}$ is the predicted cluster. $p\left( a \right)$, $p\left( b \right)$ and $p\left( a,b \right)$ are the probabilities that the cell belongs to cluster a, cluster b and both, respectively. For both ARI and NMI, higher values indicate better clustering performance.

For cell cycle dynamics, the dataset GSE64016 (with known cell cycle label G1, S, and G2M) was used and the Seurat function CellCycleScoring() was applied. Briefly, the cell cycle status and score for each cell was predicted by the Seurat algorithm. To compare the performance of the predictions in each imputation, Wilcoxon rank sum tests were performed on the predicted cell cycle scores between different known cell cycles (i.e., S vs. Others, G2M vs. Others) and the p-values were compared. The prediction accuracy (predicted cell cycle vs. true cell cycle) and the F1 score were calculated for each imputation.

To visualize the cell type/cell cycle clustering, PCA was performed followed by running UMAP with first 50 PCs. The two-dimension UMAP plots were generated for clusters/cell types/cell cycles.

To compare the clustering metrics among different methods, we first subtracted all the ARI and NMI values by the results of the unimputed data (vice versa for $H_{acc}$ and $H_{pur}$ for better visualization) and then took the median values of the three datasets. Extreme values were limited to a cutoff value for better visualization in plots. UMAP plots were generated for CellBench 10X 5CL and GSE64016 to evaluate the performance of imputations on dimension reductions. All the analyses were performed with the default parameters unless other specified.

**Note S6.**

Clustering is the essential step for exploring subtypes. In general, various imputation enhanced cell clustering to various extents. Using Louvain and K-means algorithms on three mixture datasets with four clustering metrics (Entropy of Accuracy ($H_{acc}$), Entropy of Purity ($H_{pur}$), Adjusted Rand Index (ARI) and Normalized Mutual Information (NMI)), afMF and MAGIC-log showed improvements across all metrics compared to no-imputation (**Figure 3A**). AutoClass and ccImpute performed well only with Louvain algorithms, while MAGIC and ALRA performed better with K-means algorithms. In UMAP visualization for CellBench-10X5CL, afMF, ALRA, Bfimpute, ccImpute and scRMD remained similar and consistent structure of cell clusters, while others (e.g., kNN_smoothing, DCA, I_impute) showed artifacts by producing strange shapes and generated unexpected cell cluster patterns that might lead to false discoveries (**Figure S20-21**).

Cell cycle dynamics have been well studied at single cell level. Using Seurat Cell Cycle function on a cell cycle dataset with ground truth labels, we observed some improvements of the predicted cell cycle scores after imputed by afMF, ALRA, kNN-smoothing and MAGIC/MAGIC-log. The statistical significance of the comparisons of predicted cell cycle scores between different known cell cycles were improved in these imputations (**Figure 3B**). The cell cycle prediction accuracy and F1 scores were higher in afMF, ALRA and AutoClass as well (**Figure S22**). As a results, more distinct and clearer separations between different cell cycles were also observed with data after imputation by MAGIC, afMF and ALRA in UMAP (**Figure 3C**).

**Reference**

1. Hou W, Ji Z, Ji H, Hicks SC. A systematic evaluation of single-cell RNA-sequencing imputation methods. *Genome Biol*. 2020;21(1):218. doi:10.1186/s13059-020-02132-x

2. Dai C, Jiang Y, Yin C, et al. scIMC: a platform for benchmarking comparison and visualization analysis of scRNA-seq data imputation methods. *Nucleic Acids Res*. 2022;50(9):4877-4899. doi:10.1093/nar/gkac317

3. Scrucca L, Fop M, Murphy TB, Raftery AE. mclust 5: Clustering, Classification and Density Estimation Using Gaussian Finite Mixture Models. *R J*. 2016;8(1):289-317.

**Figure S20. UMAP plots for CellBench-10X5CL cell types**

**
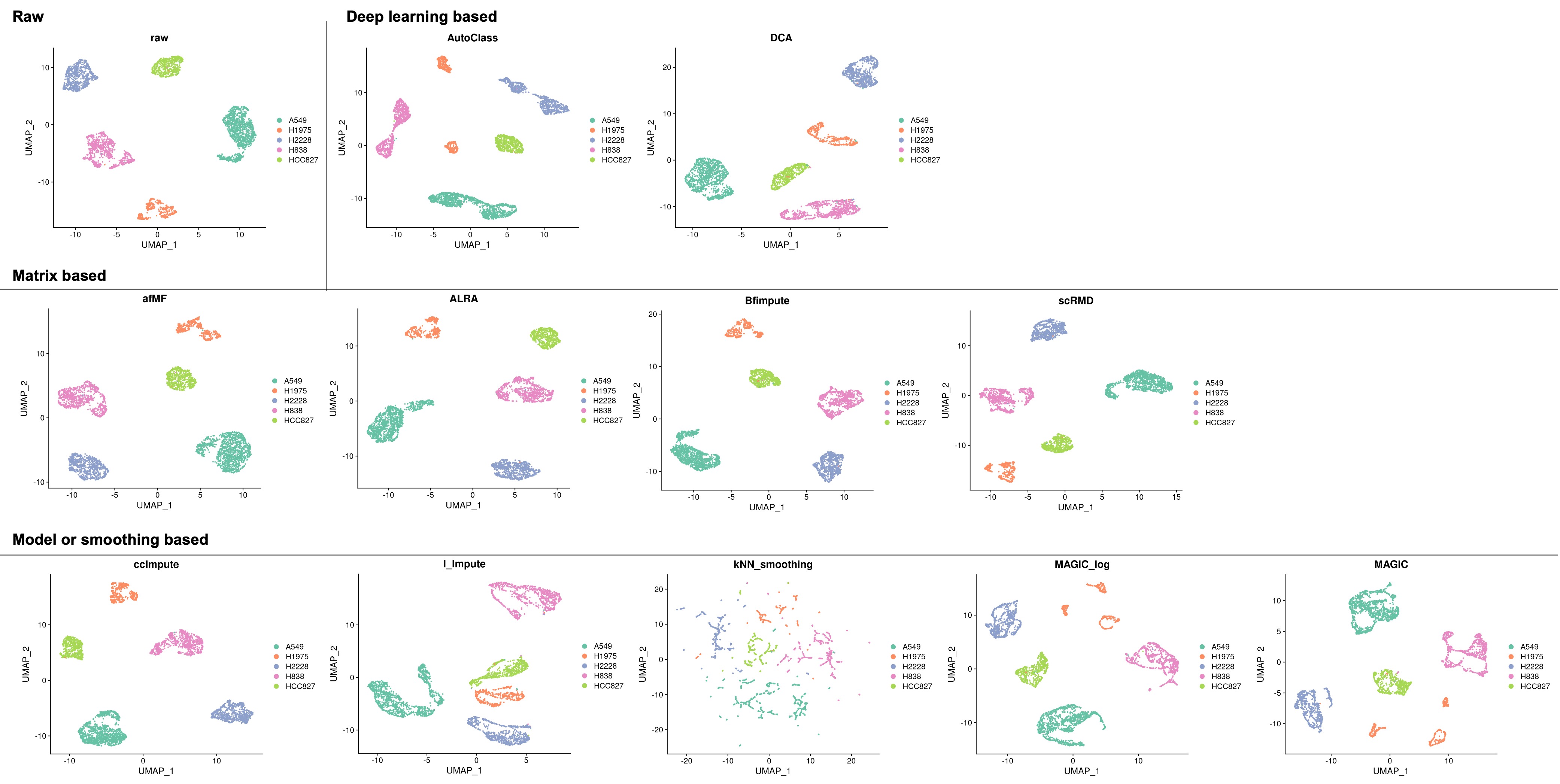
**

**Figure S21. UMAP plots for CellBench-10X5CL Louvain clusters**

**
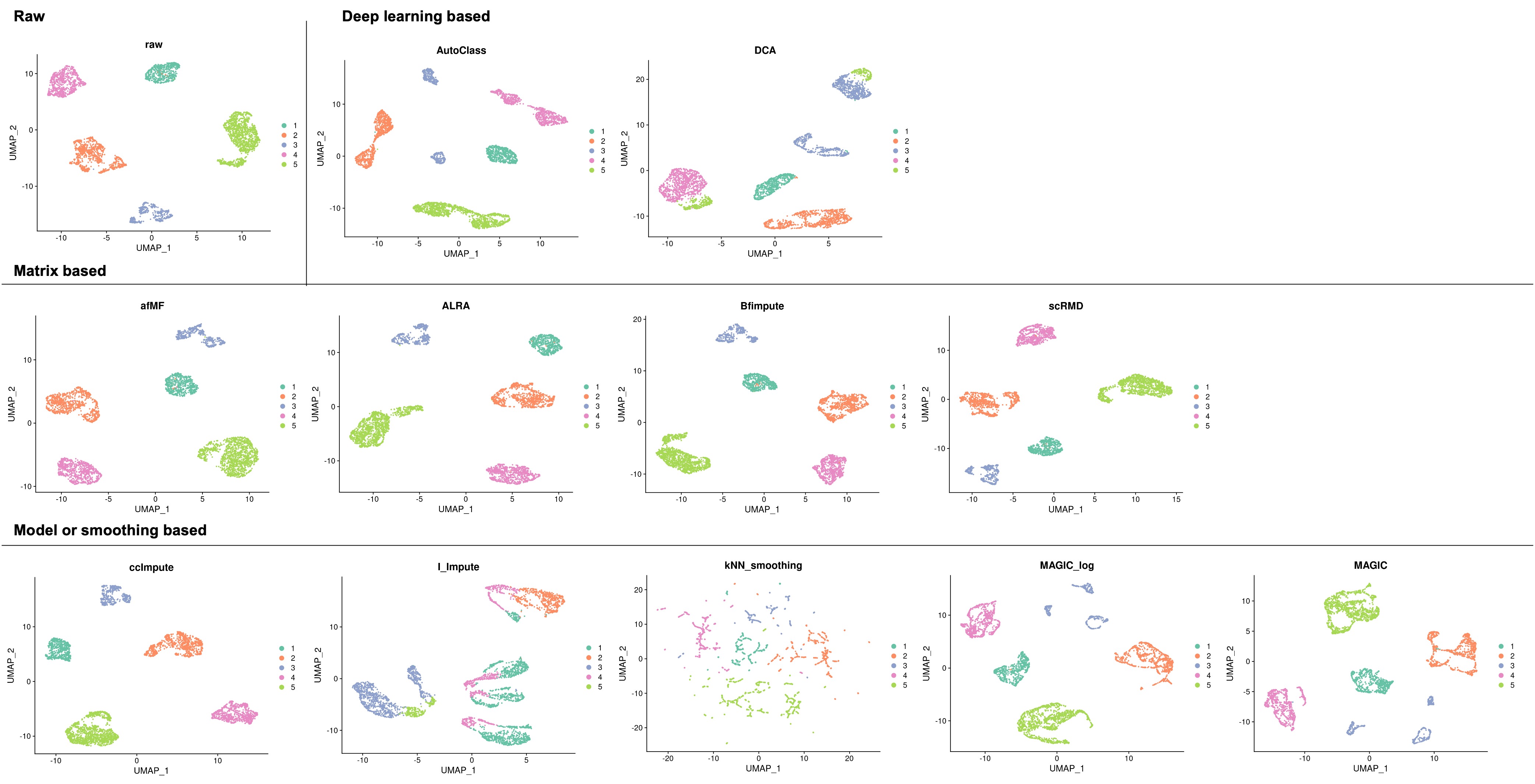
**

**Figure S22. Performance of imputations on Cell Cycle Dynamics: Seurat predicted cell cycle accuracy and F1 scores**


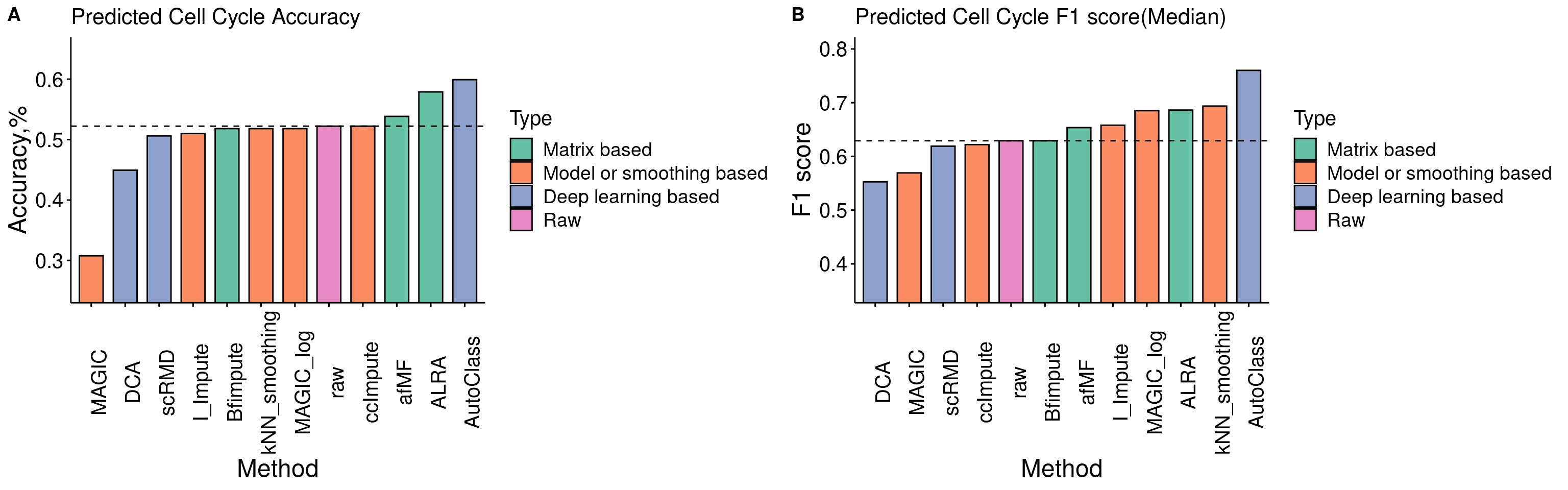

Supplement: Supplementary file 6 — Supporting Information [file CTM2-15-e70283-s005.docx]
