# Supplementary material for: An in‐depth benchmark framework for evaluating single cell RNA‐seq dropout imputation methods and the development of an improved algorithm afMF
Source: Clin Transl Med. 2025 Mar 22;15(4):e70283. doi: 10.1002/ctm2.70283 (PMC11928879; doi:10.1002/ctm2.70283)

**Method S7. Pseudotime trajectory analysis.**

*Pseudotime trajectory analysis*

The impact of imputation on pseudotime trajectory analysis was evaluated by Monocle3^1^, DPT^2^ and Slingshot^3^ on eight datasets CellBench cellmix1-4 (four datasets with two lineages), GSE118068 (embryonic and postnatal), GSE75748 time-course, GSE79578 and GSE90047 that with real time/lineage labels.

In Monocle3, the starting point of the trajectory was set based on known information and other parameters were set to default. Monocle3-normalization was also applied for comparison. Next, Spearman correlation coefficients were calculated between the predicted pseudotime and the real lineage/time labels. The Pseudo-temporal ordering score (POS) which measures cell orders were calculated using orderscore() in TSCAN package, defined as:

$$POS=\sum_{i=1}^{n-1} \sum_{j>i} g(i,j)$$

Where n is the number of cells and $g(i,j)$ is the score that evaluates how well the predicted order of the i^th^ and j^th^ cells in the ordered path matches their known order^4^. 2-D PCA and UMAP plots were generated for visualizing trajectories.

In DPT, the starting point and end point were set as ‘tips’ based on known information with other parameters set to default. Next, Spearman correlation coefficients and POS were calculated between the predicted DPT values and the real lineage/time labels. Note that due to the limitations of the software (i.e., always result in two branches), only CellBench data with two branches were used in DPT. The predicted branches by DPT were further evaluated with clustering metrics $H_{acc}$, $H_{pur}$ ARI, and NMI (as described in Clustering). 2-D diffusion map plots were generated for visualizing trajectories.

In Slingshot, the start and end point of the trajectory was set based on known information and other parameters were set to default. Next, Spearman correlation coefficients and POS were calculated between the predicted pseudotime and the real lineage/time labels. For trajectory with multiple branches, the predicted branches and the real branches were matched by their largest overlaps of cells. For instance, for datasets of three cell types (A, B, and C where A will differentiate to B and C) with even number, ideally predicted branch 1 would have 100% overlap rate with A-to-B known branch while only have 50% overlap rate with A-to-C known branch (vice versa for predicted branch 2). Then the true overlap rates (i.e., largest overlaps between predicted branches and real branches, as matched branches) were calculated and compared. 2-D PCA plots were generated for visualizing Slingshot results. Slingshot-normalization was applied for comparison as well.

The metric values were subtracted by the results of the unimputed log-normalized data. Extreme values were limited to a cutoff value for better visualization.

**Note S7.**

Pseudotime trajectory analysis enables the study of cell differentiation and development. Though it has been evaluated previously, here we used imputed data with three more well-established algorithms: DPT, monocle3, and Slingshot with additional datasets.

When using DPT, ALRA, afMF, MAGIC/MAGIC-log and ccImpute improved the pseudotime analysis (**Figure 7D** and **Figure S23**). The predicted branches were further evaluated by the clustering metrics with true branch labels as ground truth. ALRA, afMF, ccImpute and AutoClass showed improvements in branch predictions (**Figure 7D right**). In diffusion map visualization, afMF, ALRA and AutoClass showed better continuum trajectories and branches, while others either showed less improvements or heavily distorted the trajectory patterns (**Figure S23**).

As shown in **Figure S24** for monocle3, higher Spearman correlations and pseudo-temporal ordering scores (POS) between predicted pseudotime and real time were observed in MAGIC/MAGIC-log with statistical significance. While afMF and AutoClass only showed slight improvements, the rest of the algorithms had no or negative influence. In 2-D PCA visualizations for cell types and pseudotime (**Figure S24C-D**), afMF and ALRA showed relatively consistent patterns as no-imputation, while MAGIC-log presented more concentrated and dense trajectories as cells from the same cell types were much closer to each other and formed clearer continuum.

On the other hand, Slingshot were found to be incompatible with most imputation algorithms as imputed data gave inferior or no improvement in trajectory analysis compared to raw data (**Figure S25**).

**Reference**

1. Qiu X, Mao Q, Tang Y, et al. Reversed graph embedding resolves complex single-cell trajectories. *Nat Methods*. 2017;14(10):979-982. doi:10.1038/nmeth.4402

2. Haghverdi L, Büttner M, Wolf FA, Buettner F, Theis FJ. Diffusion pseudotime robustly reconstructs lineage branching. *Nat Methods*. 2016;13(10):845-848. doi:10.1038/nmeth.3971

3. Street K, Risso D, Fletcher RB, et al. Slingshot: cell lineage and pseudotime inference for single-cell transcriptomics. *BMC Genomics*. 2018;19(1):477. doi:10.1186/s12864-018-4772-0

4. Dai C, Jiang Y, Yin C, et al. scIMC: a platform for benchmarking comparison and visualization analysis of scRNA-seq data imputation methods. *Nucleic Acids Res*. 2022;50(9):4877-4899. doi:10.1093/nar/gkac317

**Figure S23. Diffusion map plots using DPT (raw, afMF and MAGIC)**

**
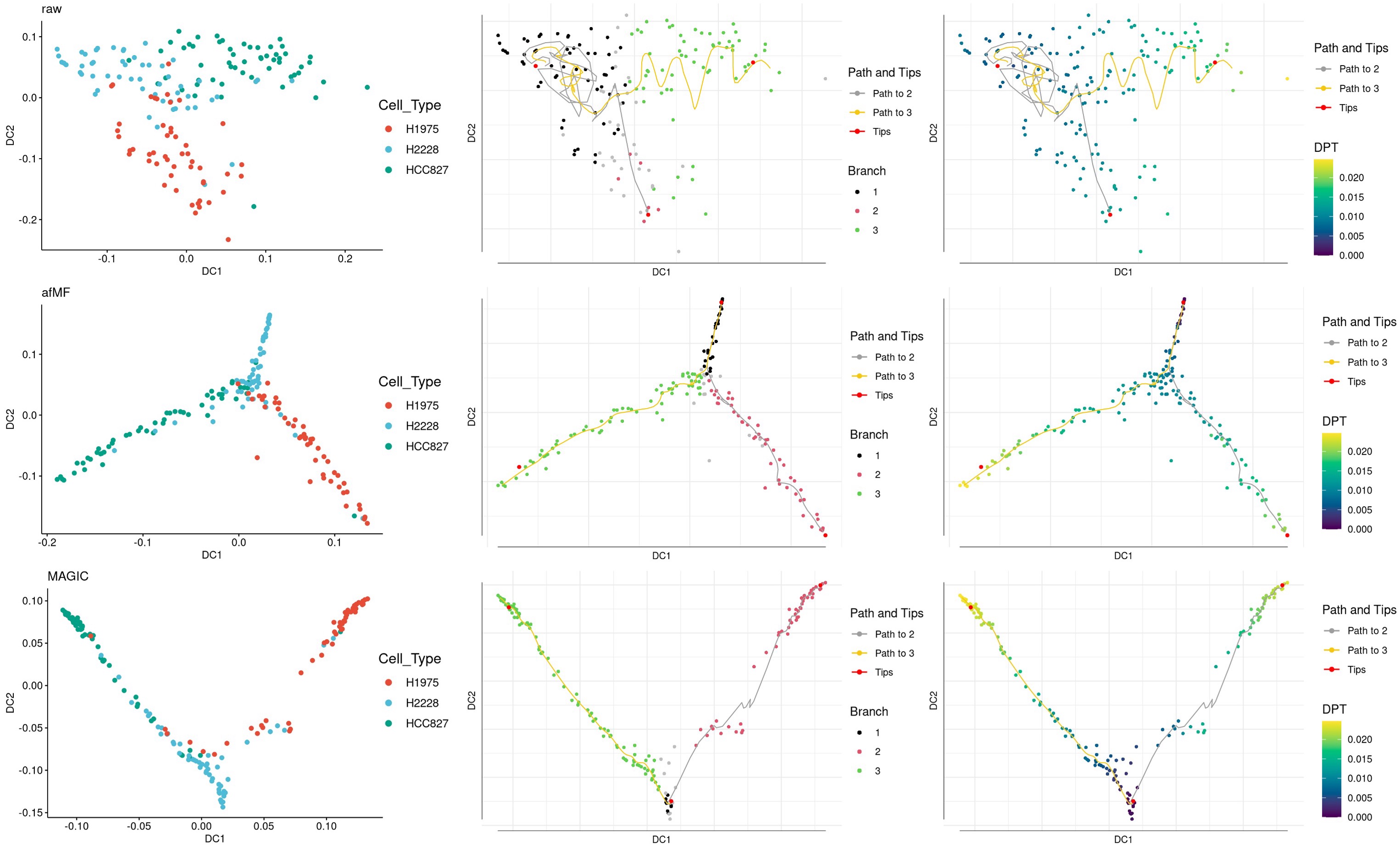
**

**Figure S24. Performance of imputations on Pseudotime Trajectory Analysis: Monocle3.**


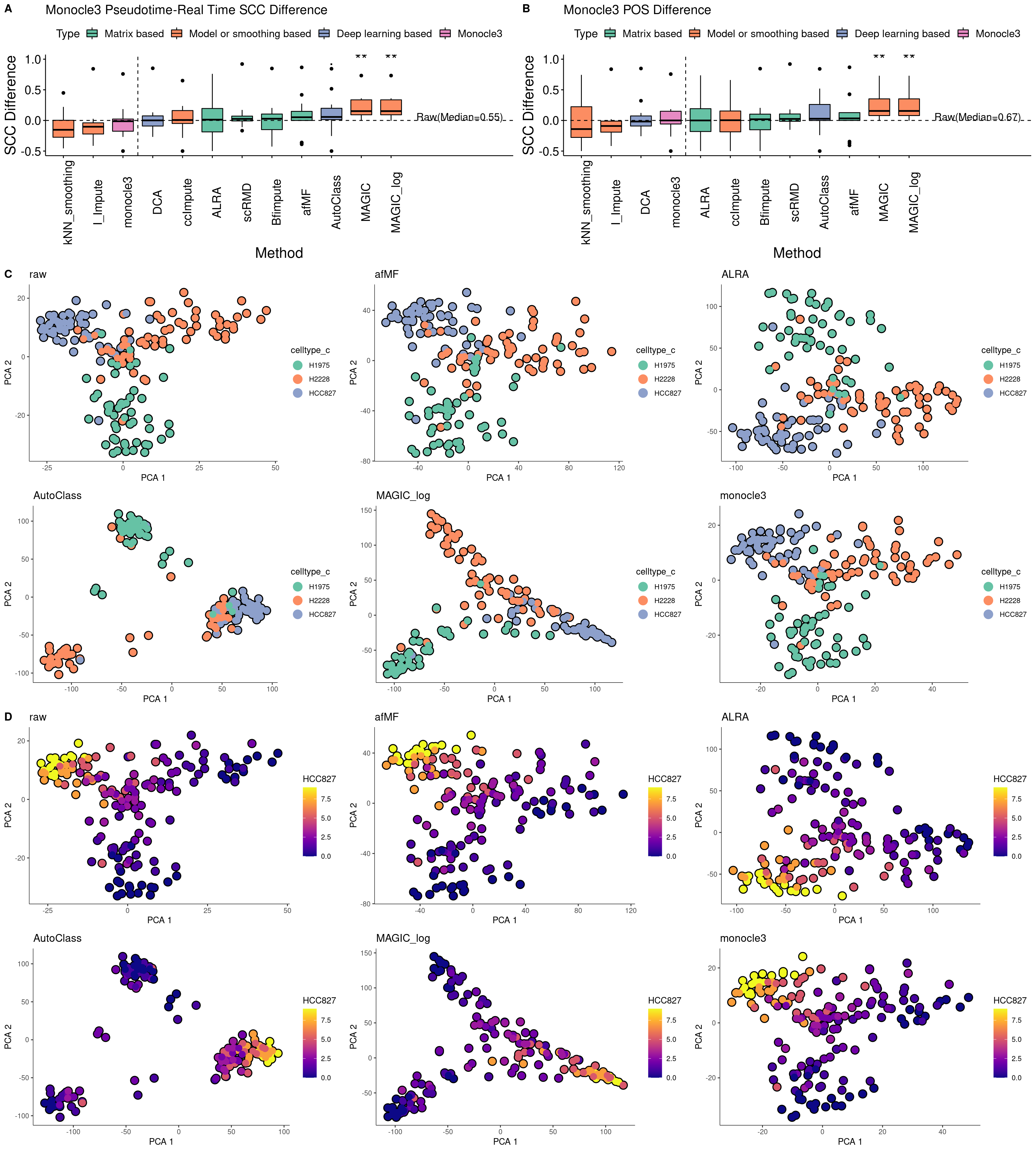


**Figure S25. Performance of imputation on Pseudotime Trajectory Analysis: Slingshot**


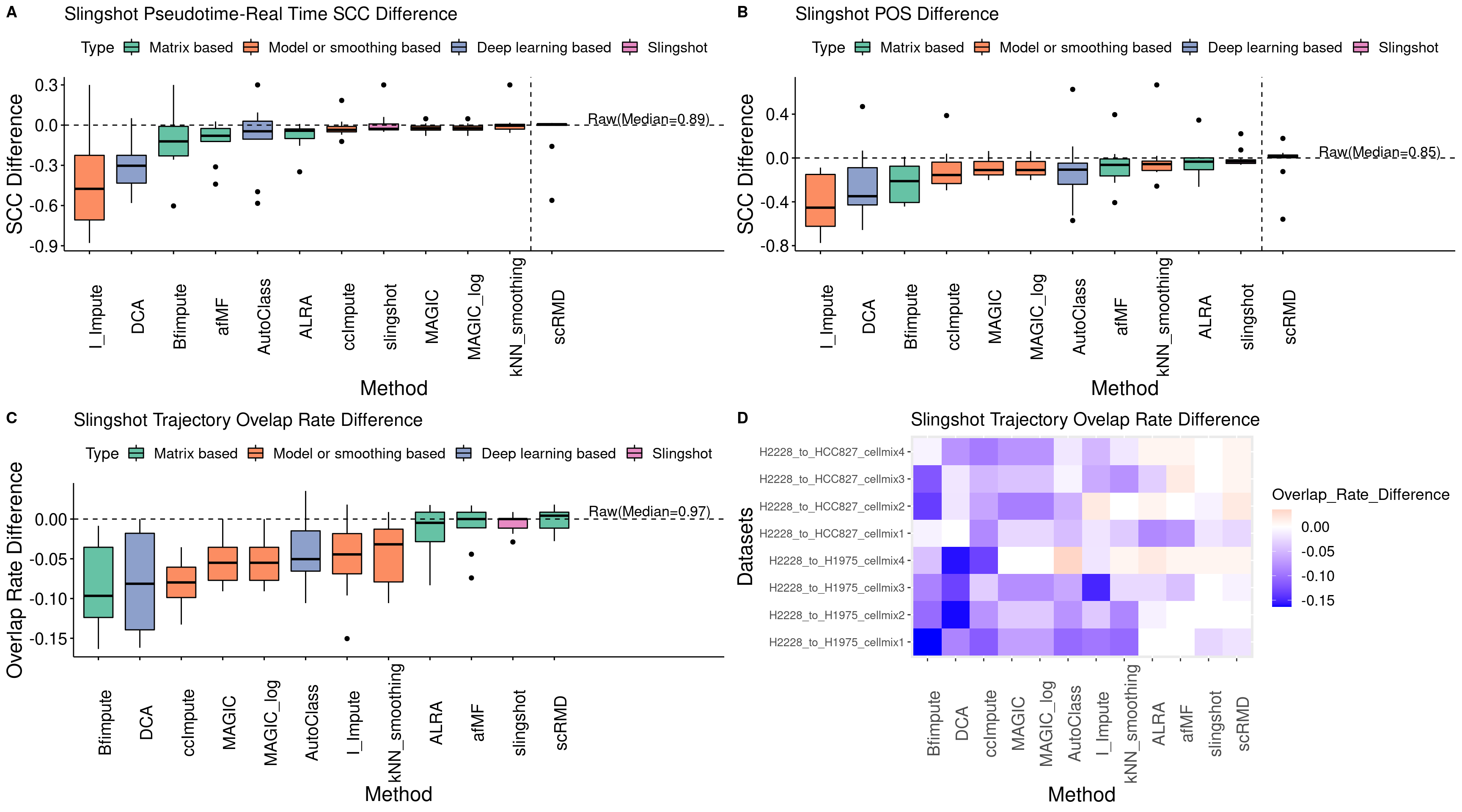

Supplement: Supplementary file 7 — Supporting Information [file CTM2-15-e70283-s010.docx]
