# Supplementary material for: An in‐depth benchmark framework for evaluating single cell RNA‐seq dropout imputation methods and the development of an improved algorithm afMF
Source: Clin Transl Med. 2025 Mar 22;15(4):e70283. doi: 10.1002/ctm2.70283 (PMC11928879; doi:10.1002/ctm2.70283)
Supplement: Supplementary file 8 — Supporting Information [file CTM2-15-e70283-s007.docx]

**Method S8. Advanced analysis: AUCell & SCENIC and cell-cell communication.**

*AUCell for pathway activation and SCENIC for gene regulatory analysis*

We evaluated the AUCell application among different imputations on GSE155673, where the activations of the interferon (IFN) related pathways between COVID-19 and healthy controls were compared within monocytes. Briefly, the IFN-related pathways were collected from both Hallmark^1^ gene sets (2 terms) and GO^2^ gene sets (31 terms). Each cell was then assigned a binary status (activated or not) for each pathway by the AUCell pipeline and the percentages of monocytes with activated pathway were compared between COVID-19 and healthy controls. All the analyses were performed with the default parameters unless other specified.

We next investigated the compatibility of SCENIC application with different imputations. We followed the pipeline as previously suggested^3^ using the same dataset GSE115978 and the various normalized/imputed data were used as input. Regulons with Z-score$\geq$3 for each of the method were collected and visualized through heatmap and Venn diagram for comparison. Next, seven well-established regulons (i.e., TCF7, EOMES, TBX21, MITF, MYC, MAFB, PAX5) as previously demonstrated^3^ were picked out. AUCell (implemented in SCENIC) were used to determine whether the selected regulon was activated in a selected cell. Ideally, a cell-type-specific regulon was expected to be activated only in the specific cell type, but not the other cell types. The percentages of cells that with activated regulons within expected cell types and other cell types were calculated and compared. The metric values were subtracted by the results of the unimputed log-normalized data. Extreme values were limited to a cutoff value for better visualization. All the analyses were performed with the default parameters unless other specified.

*CellPhoneDB and CellChat for Cell-Cell Communication*

We evaluated the potential impacts of imputation on cell-cell communications using the two packages CellPhoneDB^4^ and CellChat^5^ on datasets E-MTAB-6701, CellBench-10x-5cl (GSE126906), GSE75748-cell-types and E-MTAB-13384. For CellPhoneDB, we collected the interaction pairs of ligand-receptors-cell type as used in previous protocol^4^ and followed the pipeline with all the parameters set to default. Bubble plots showing active interactions with adjusted p-values and mean expressions were generated for comparisons. The adjusted P-values and the proportion of significant interactions under different significant levels were compared. In CellChat analysis, we aimed to evaluate the communications from monocytes/macrophages to fibroblasts or other cell types as demonstrated in previous paper^6^. Note that in the original research this communication was not replicated in this mice dataset and therefore we aim to verify if imputation could make validations. We followed the pipeline with all the parameters set to default and normalized/imputed data as input. The numbers/percentages of significant interactions (all or from-monocyte, stratified by CHIP status) were compared. Additionally, network plots for source-Monocyte were generated for comparisons.

*Integration with spatial transcriptomics*

A spatial transcriptomics mouse brain dataset (stxBrain) (<https://support.10xgenomics.com/spatial-gene-expression/datasets>) and the reference scRNA-seq dataset (adult mouse cortical cell taxonomy)^7^ were used for evaluating the impact of imputation on the integration of spatial transcriptomics with scRNA-seq. The Seurat integration pipeline^8^ was used and the four top imputation algorithms ALRA, MAGIC, AutoClass and ALRA were compared to the recommended algorithm Seurat SC-Transform. The reference scRNA-seq data was imputed by these algorithms. Because ground truth cell type labels are not available for spatial transcriptomics data, we used prior knowledge of spatial organization to make evaluations. Within spatial data, we subset the frontal cortex region and focused on the known spatial localization patterns of both neuronal and non-neuronal subsets, including laminar excitatory, layer-1 astrocytes, and the cortical grey matter. The cell type prediction scores resulted from different algorithms were compared and visualized using SpatialFeaturePlot() in Seurat.

**Note S8.**

AUCell is an advanced application for investigating the activities of gene sets (e.g., pathways) in each cell. As there is no ground truth, a well-studied pathway (i.e., interferon (IFN)) between COVID-19 and healthy controls were used. As shown in **Figure 3E**, in afMF and ALRA-imputed data, increased percentages of monocytes with Hallmark IFN-Alpha/Gamma response activated were only observed within COVID-19 subjects (true positive); in contrast, cells remained low levels of activation within healthy controls (true negative) as expected. On the other hand, other imputation algorithms showed IFN activation in both normal (false positive) and COVID-19 subjects (true positive) at the same time. When projected by UMAP, after imputation by AutoClass, activation of both IFN-Alpha and IFN-Gamma were found among healthy controls (normal cells), which were false positives activation (**Figure 3F**). Similar conclusions were obtained for GO IFN-related pathways. These results suggested that only Matrix based algorithms (ALRA and afMF) better revealed activation of IFN pathways in monocytes in COVID-19 patients while avoiding having excessive false positive activation among healthy controls.

SCENIC is an advanced application that incorporates AUCell for exploring gene regulatory networks and transcriptional factors. Similar as for IFN in AUCell, seven well-established cell-type-specific ‘regulons’ were selected as previously demonstrated^3^. afMF, MAGIC/MAGIC-log and AutoClass performed well as they increased the percentages of cells with activated regulons within expected cell types (correct calls) while remained consistent levels as no-imputation within other unrelated cell types (false positive calls) (**Figure 3G**). Next, we compared all the identified regulons (Z-score>3) across all the cell types and observed that most selected imputation methods could recover the patterns in raw data but also added some unique significant regulons (**Figure 3H** and **Figure S26**). Of note, these newly generated significant regulons should be further validated through other experiments.

CellPhoneDB and CellChat are two popular tools that enable researchers to study cell-cell communication networks through ligand-receptor databases. However, in CellPhoneDB analysis using three well-established datasets, abnormally huge increments of significant interactions were discovered after imputations (**Figure S27-29)**. Though no ground truth is available for demonstration, they were believed to be the false positives as most of the pairwise cell-cell interaction were detected only after imputation (e.g., MAGIC, AutoClass, afMF). Similar as for CellChat analysis, we also found large increase of significant interactions after imputations (**Figure S30**); on the other hand, we discovered that CHIP status had more interactions than NO-CHIP status after imputations, which was expected from biological perspective.

Integrating with scRNA-seq data is an important step to study spatial transcriptomics. A spatial mouse brain dataset (stxBrain) and the reference scRNA-seq dataset (adult mouse cortical cell taxonomy) were used to evaluate the impact of imputation on the integration of spatial data with scRNA-seq data. Using Seurat integration pipeline, we observed a clear recovery of the known spatial localization patterns of both neuronal and non-neuronal subsets with raw SC-Transform data as reference (**Figure S31**). Reference using MAGIC-imputed data replicated the patterns generally, while ALRA led to much weaker cell type prediction scores within the specific locations (e.g., L4 and L5 PT/IT regions). AutoClass did not generate any pattern, and no algorithms were found to make obvious enhancement. When running with afMF, an error was returned due to unknown technical reason which suggested the incompatibility between afMF imputed normalized matrix and Seurat integration pipeline. Notably, many integration methods including RCTD and Seurat recommended to use untransformed count-level scRNA-seq data or their specific transformation (e.g., sc-transform in Seurat) as reference input, and thus most of the outputs of the imputation methods did not meet these criteria as they were designed for normalized data or had internal normalization / transformation step. This explained why some imputation methods such as AutoClass and afMF did not work well for this task. The compatibilities between imputation algorithms and these integration algorithms can be addressed in the future and other new integration algorithms could also be tested to see if they would benefit from imputation.

**Reference**

1. Subramanian A, Tamayo P, Mootha VK, et al. Gene set enrichment analysis: a knowledge-based approach for interpreting genome-wide expression profiles. *Proc Natl Acad Sci U S A*. 2005;102(43):15545-15550. doi:10.1073/pnas.0506580102

2. Ashburner M, Ball CA, Blake JA, et al. Gene ontology: tool for the unification of biology. The Gene Ontology Consortium. *Nat Genet*. 2000;25(1):25-29. doi:10.1038/75556

3. Van de Sande B, Flerin C, Davie K, et al. A scalable SCENIC workflow for single-cell gene regulatory network analysis. *Nat Protoc*. 2020;15(7):2247-2276. doi:10.1038/s41596-020-0336-2

4. Efremova M, Vento-Tormo M, Teichmann SA, Vento-Tormo R. CellPhoneDB: inferring cell-cell communication from combined expression of multi-subunit ligand-receptor complexes. *Nat Protoc*. 2020;15(4):1484-1506. doi:10.1038/s41596-020-0292-x

5. Jin S, Guerrero-Juarez CF, Zhang L, et al. Inference and analysis of cell-cell communication using CellChat. *Nat Commun*. 2021;12(1):1088. doi:10.1038/s41467-021-21246-9

6. Shumliakivska M, Luxán G, Hemmerling I, et al. DNMT3A clonal hematopoiesis-driver mutations induce cardiac fibrosis by paracrine activation of fibroblasts. *Nat Commun*. 2024;15(1):606. doi:10.1038/s41467-023-43003-w

7. Tasic B, Menon V, Nguyen TN, et al. Adult mouse cortical cell taxonomy revealed by single cell transcriptomics. *Nat Neurosci*. 2016;19(2):335-346. doi:10.1038/nn.4216

8. Hao Y, Hao S, Andersen-Nissen E, et al. Integrated analysis of multimodal single-cell data. *Cell*. 2021;184(13):3573-3587.e29. doi:10.1016/j.cell.2021.04.048

**Figure S26. Comparisons of all the imputation-identified regulons (Z-score>3) across all the cell types**

**
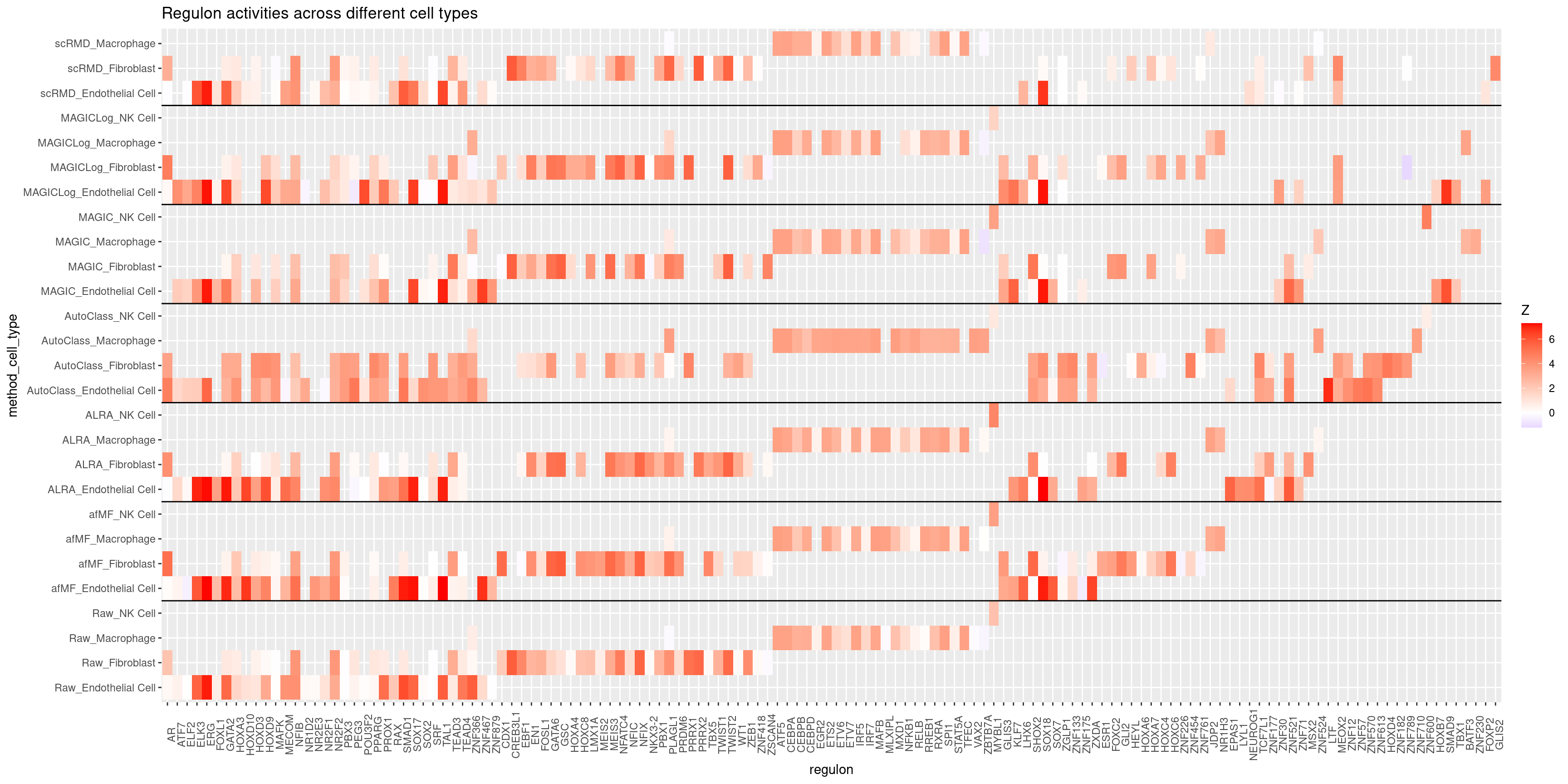
**

**Figure S27. Comparisons of ligand-receptor cell-cell communications across different imputations using CellPhoneDB**

**
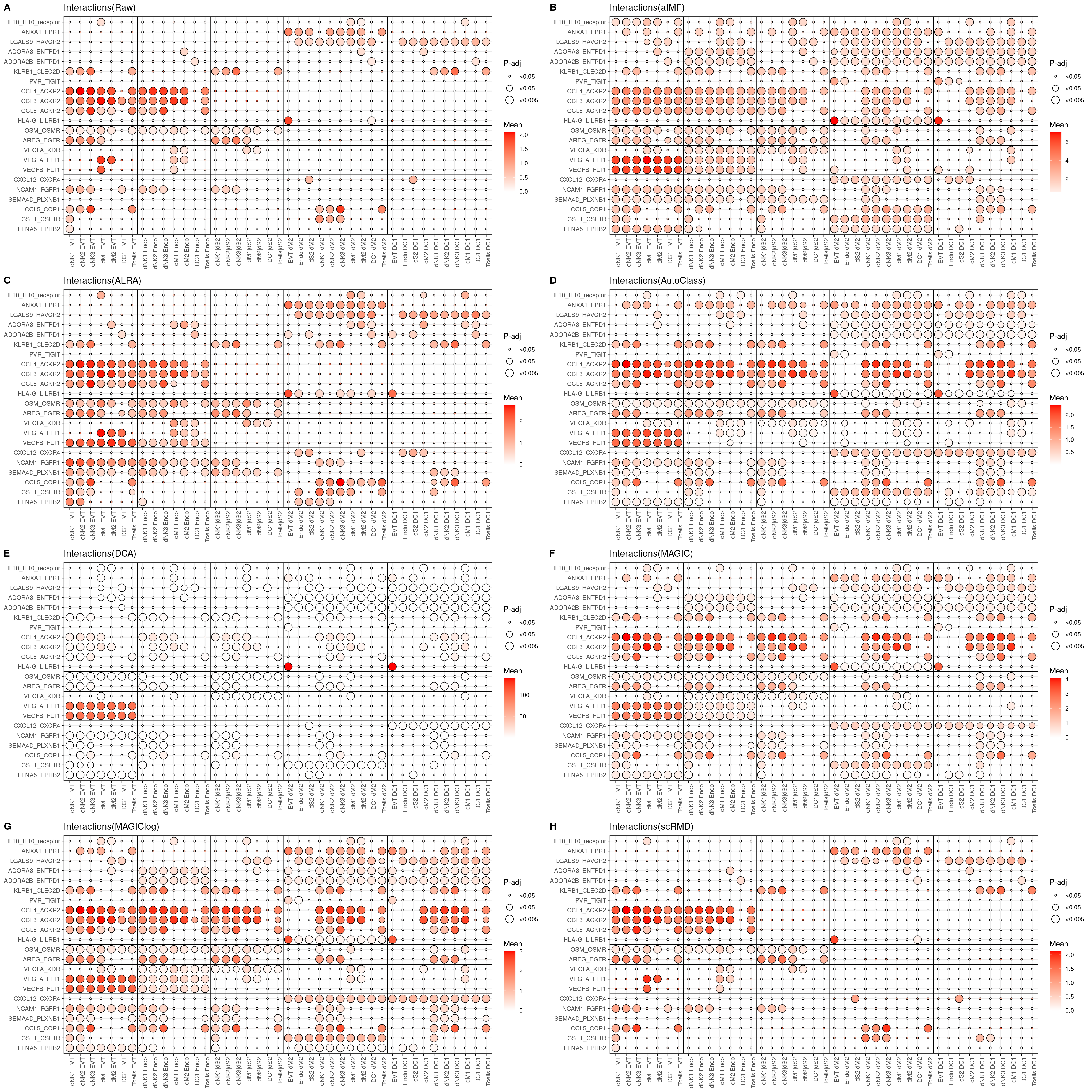
**

**Figure S28. Performance of imputations on cell-cell communications using CellPhoneDB (E-MTAB-6701)**


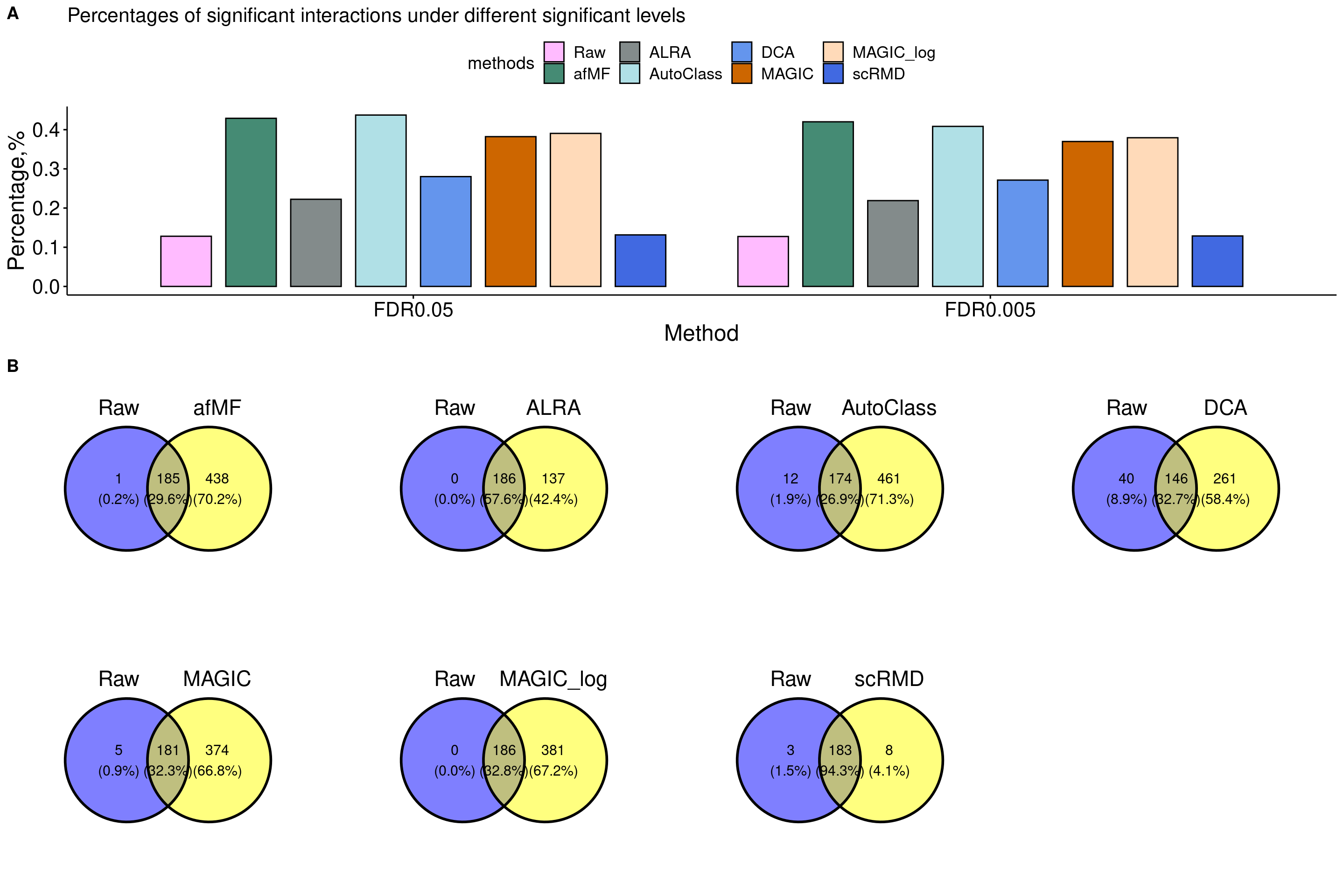


**Figure S29. Performance of imputations on cell-cell communications using CellPhoneDB (Cell line datasets)**


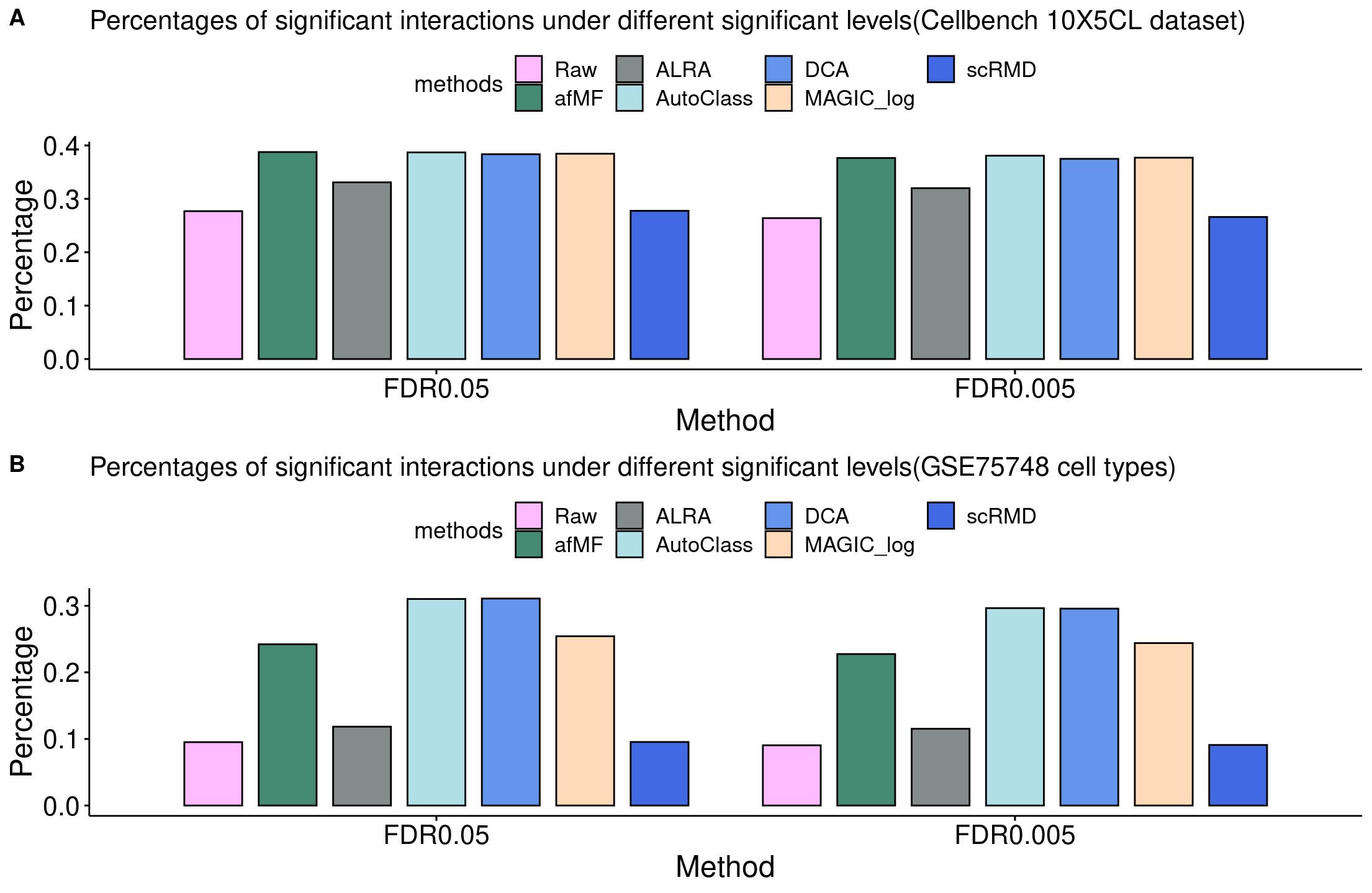


**Figure S30. Performance of imputations on cell-cell communications using CellChat**

**Figure S31. Performance of imputations on integration of spatial transcriptomics with scRNA-seq data**

**
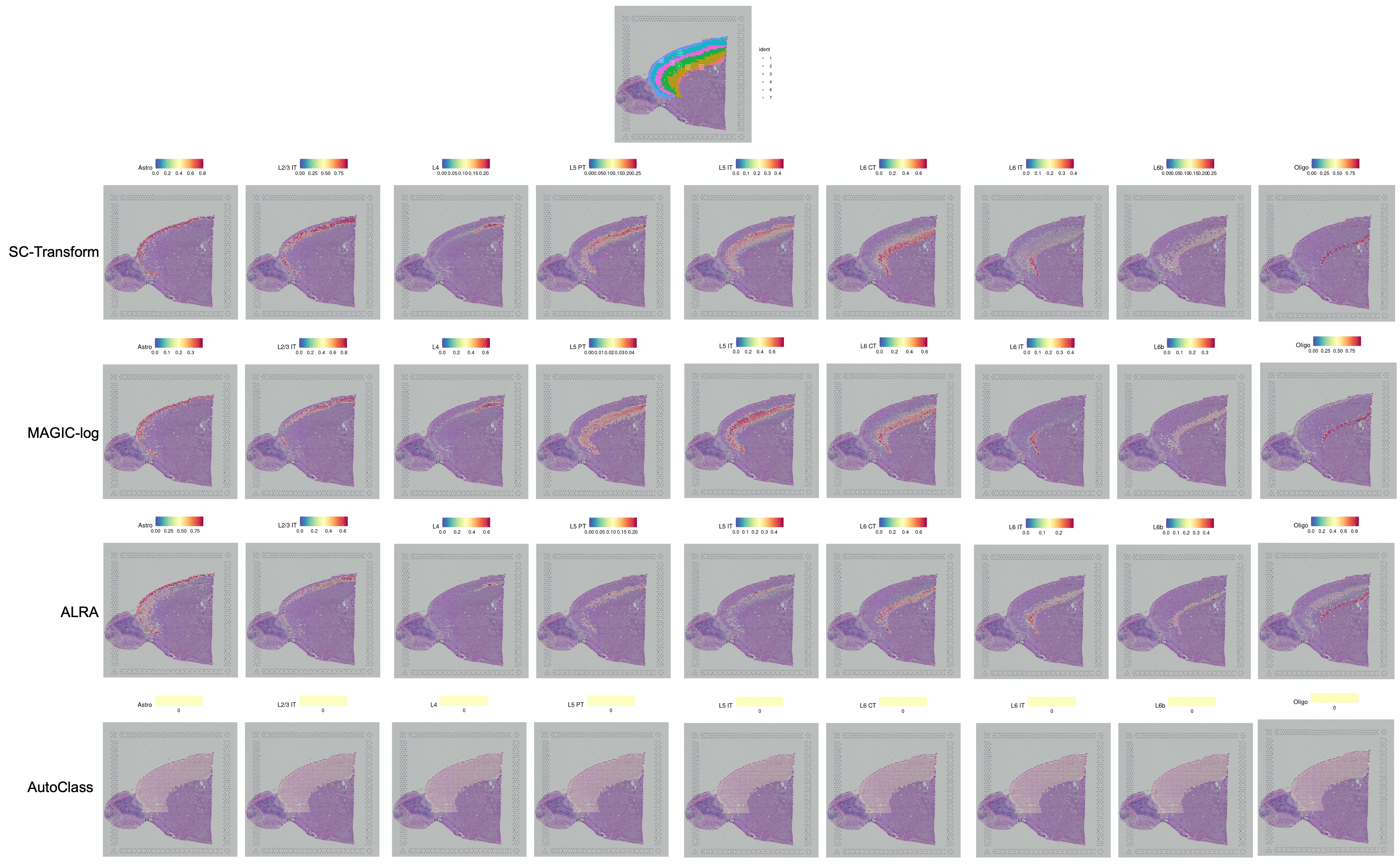
**
