# Supplementary material for: An in‐depth benchmark framework for evaluating single cell RNA‐seq dropout imputation methods and the development of an improved algorithm afMF
Source: Clin Transl Med. 2025 Mar 22;15(4):e70283. doi: 10.1002/ctm2.70283 (PMC11928879; doi:10.1002/ctm2.70283)
Supplement: Supplementary file 9 — Supporting Information [file CTM2-15-e70283-s002.docx]

**Method S9. Simulated data analysis.**

*Simulated data analysis*

The simulated datasets with ground truth were generated using Splatter^1^ (Mock90) and SplatPop^2^ (SplatPop90) with parameters shown in **Table S1**. The evaluations for simulated data included Differential Expression Analysis, Classification, Biomarker Prediction, Automatic Cell Type Annotation, Dimension reduction & Clustering, and Imputed SC-Ground Truth Similarity as described previously. Note that the ground truth (e.g., known DEGs, groups, true matrix) instead of bulk data were used as gold standard in these evaluations.

*Distinguish dropouts and real biological zeros and calculate balanced accuracy*

We used the Mock90 dataset (where the real biological zeros and technical dropouts are known) to explore the performances of various imputation algorithms on the balance of dropout imputation and biological zero preservation. This evaluation was once proposed in previous study^3^, and here we calculated the confusion matrix of various imputations:

|  | **Imputed** | **Not imputed** |
| --- | --- | --- |
| **Dropouts** | True positive (TP) | False negative (FN) |
| **Real biological zeros** | False positive (FP) | True negative (TN) |

Then the sensitivity, specificity and balanced accuracy were calculated as follows:

Sensitivity = TP / (TP+FN)

Specificity = TN / (FP+TN)

Balanced accuracy = (Sensitivity+ Specificity) / 2

**Note S9.**

Real-life scRNA-seq data are complicated and difficult to simulate. Nonetheless, simulated datasets have the advantage that they come with the ground truth. Using simulated datasets generated from Splatter and SplatPop, we found afMF, ALRA, MAGIC/MAGIC-log, AutoClass and kNN-smoothing performed generally better in DE analysis (MAST), Classification, Biomarker Prediction, Automatic Cell Type Annotation, and Cell-Ground Truth Profiling Similarity (**Figure S31-32**). MAGIC, ALRA, kNN-smoothing and afMF showed improvements in simulated data K-means clustering (**Figure S32H**), while all methods performed worse with Louvain clustering.

In distinguishing technical dropouts and biological zeros in Mock90 dataset, most of the methods increased the balanced accuracy except for two deep-learning-based methods (DCA and AutoClass) and I-impute. Generally, MAGIC and afMF can impute most of the dropouts, while ccImpute, kNN-smoothing and scRMD can preserve most of the biological zeros. ALRA, kNN-smoothing and Bfimpute are the three algorithms with the best balanced accuracy (**Figure S33**). However, we also realized two potential issues in this evaluation. First, many algorithms worked as smoothing, so they are likely to result in non-zero small float values rather than zeros which are also acceptable in various downstream analyses. Second, many imputation algorithms had their internal normalizations or specific transformations, which made the data quite different from the ground truth data that was either untransformed or transformed by one particular normalization method, e.g., Seurat log-normalization. Therefore, we reasoned that this evaluation and comparison is not quite fair for all of the methods. We believed that better performances in downstream tasks are more important than in theoretical supporting analysis.

In cell-cell correlation heatmaps for Mock90 dataset, afMF, ALRA and MAGIC / MAGIC-log improved the visualizations (**Figure S34**).

**Reference**

1. Zappia L, Phipson B, Oshlack A. Splatter: simulation of single-cell RNA sequencing data. *Genome Biol*. 2017;18(1):174. doi:10.1186/s13059-017-1305-0

2. Azodi CB, Zappia L, Oshlack A, McCarthy DJ. splatPop: simulating population scale single-cell RNA sequencing data. *Genome Biol*. 2021;22(1):341. doi:10.1186/s13059-021-02546-1

3. Zheng W, Min W, Wang S. TsImpute: an accurate two-step imputation method for single-cell RNA-seq data. *Bioinformatics*. 2023;39(12):btad731. doi:10.1093/bioinformatics/btad731

**Figure S31. Performance of imputations on simulated data with ground truth**


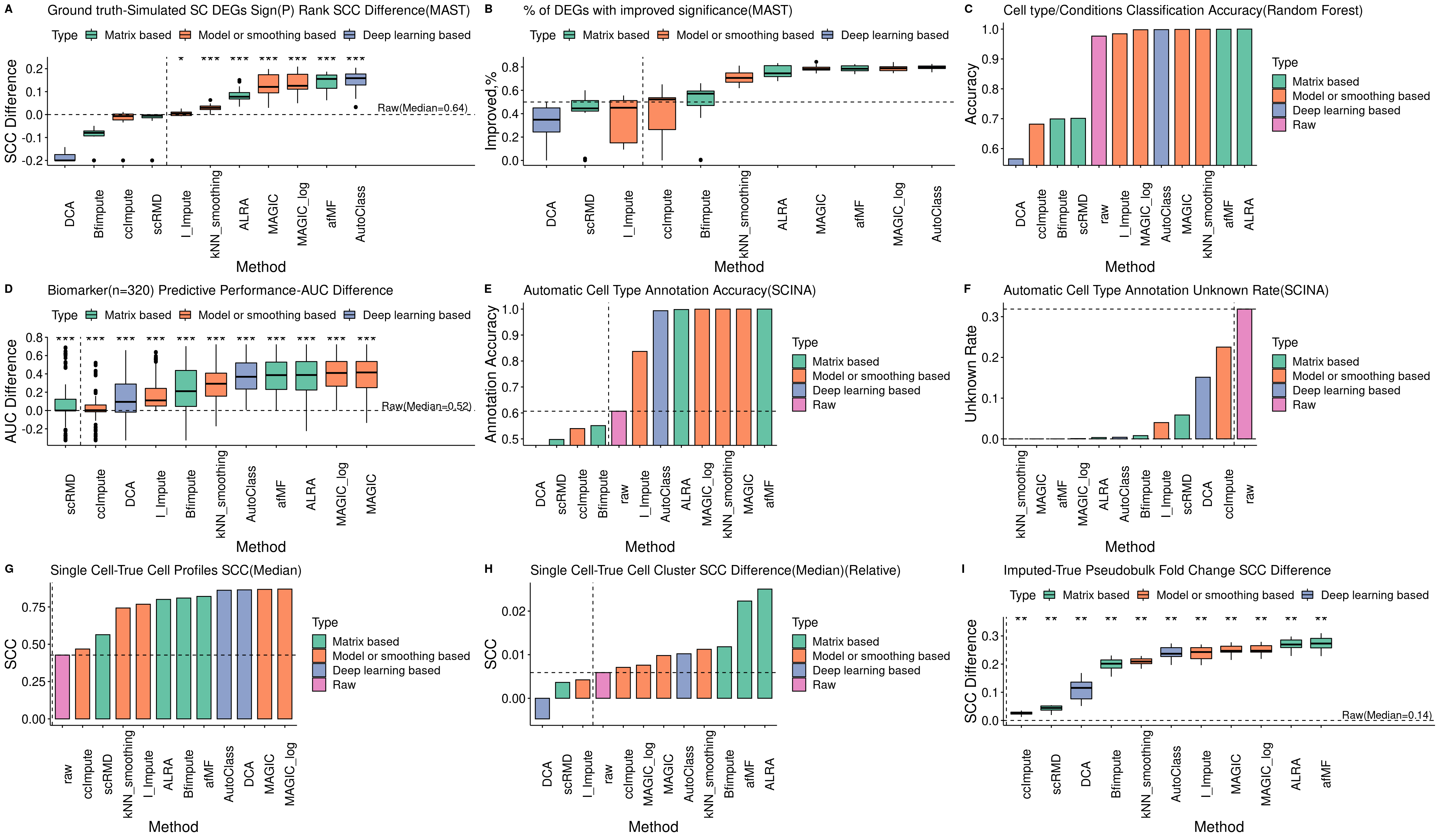


The simulated datasets with ground truth were generated using Splatter (Mock90) and SplatPop (SplatPop90). The ground truth (e.g., known DEGs, groups, matrix) instead of bulk data were used as gold standard in these evaluations. Some metrics were subtracted by the values of the raw results. Extreme values have been limited to a cutoff value for better visualization. The evaluations included: (**A-B**) Differential Expression Analysis; (**C**) Classification; (**D**) Biomarker Prediction, (**E-F**) Automatic Cell Type Annotation; Clustering (Supplementary) and (**G-I**) Imputed SC-Ground Truth Similarity (Imputed Cell-True Cell; Imputed Cell-True Cell Cluster; Imputed Pseudobulk-True Pseudobulk).

**Figure S32. Performance of imputations on simulated data with ground truth (supplementary results)**


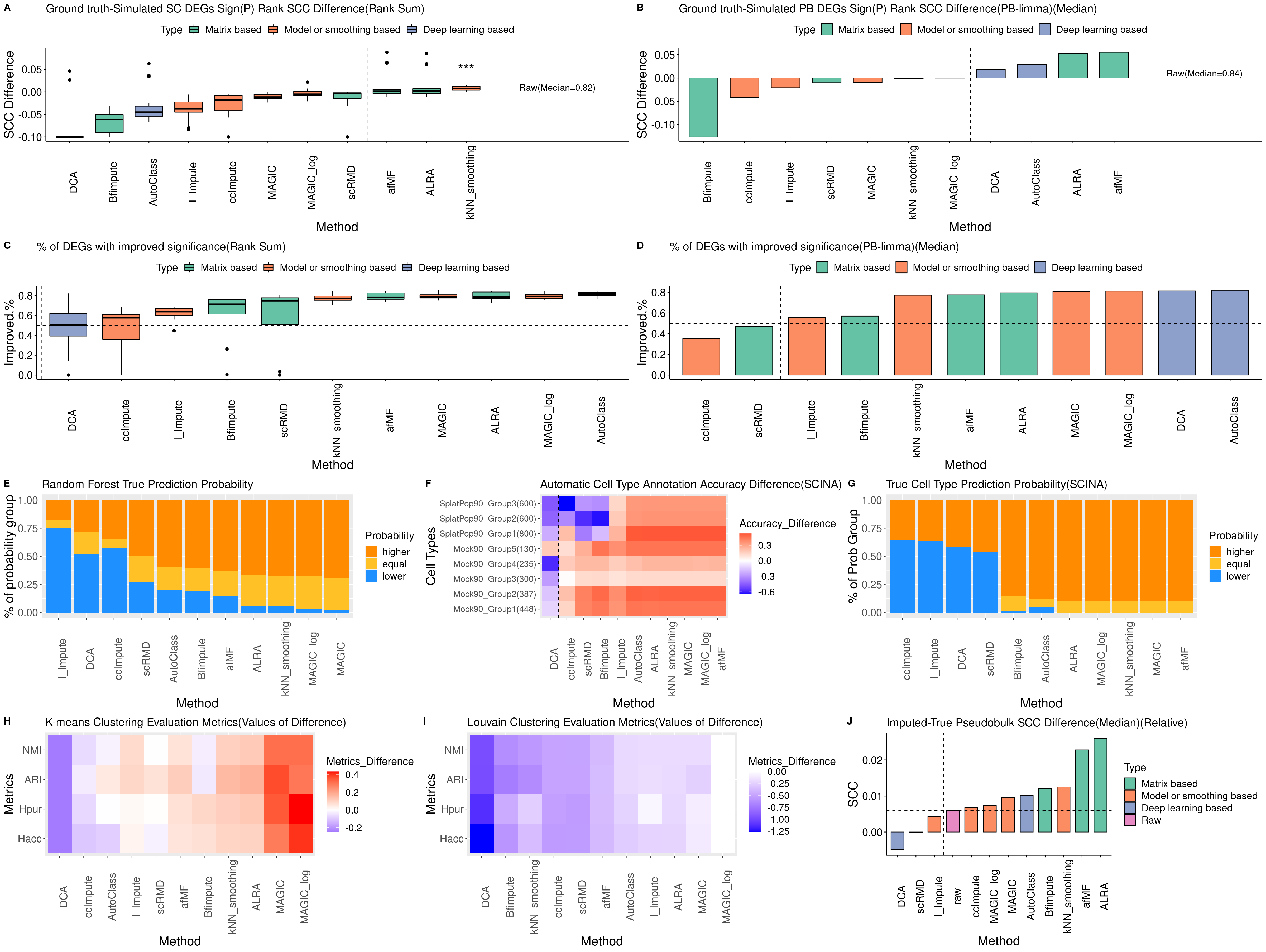


**Figure S33. Balanced accuracy and proportion of imputed dropouts and preserved real biological zeros in Mock90 dataset**

**
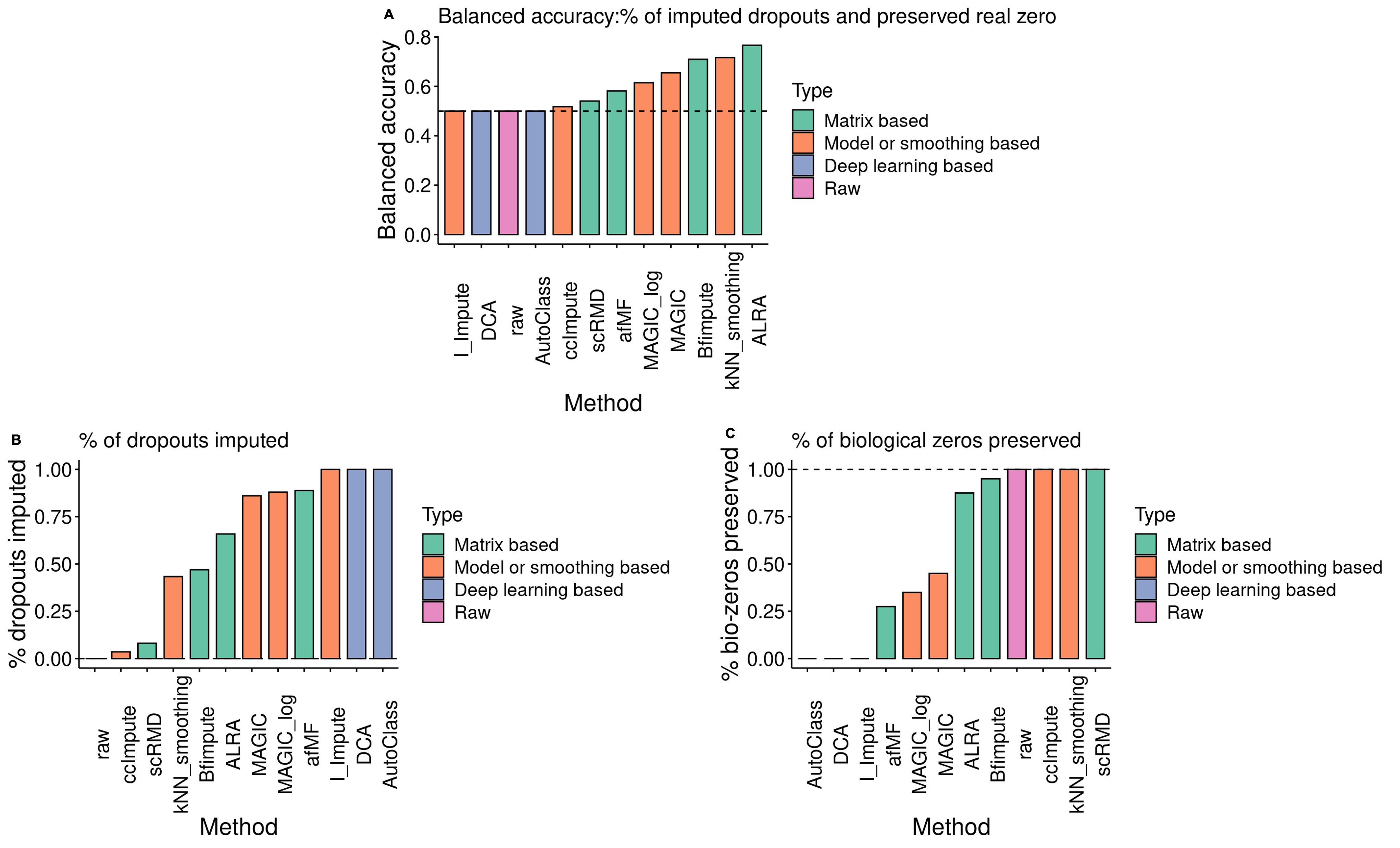
**

**Figure S34. Cell-Cell Correlation heatmaps in different imputations using simulated data (Mock90)**

**
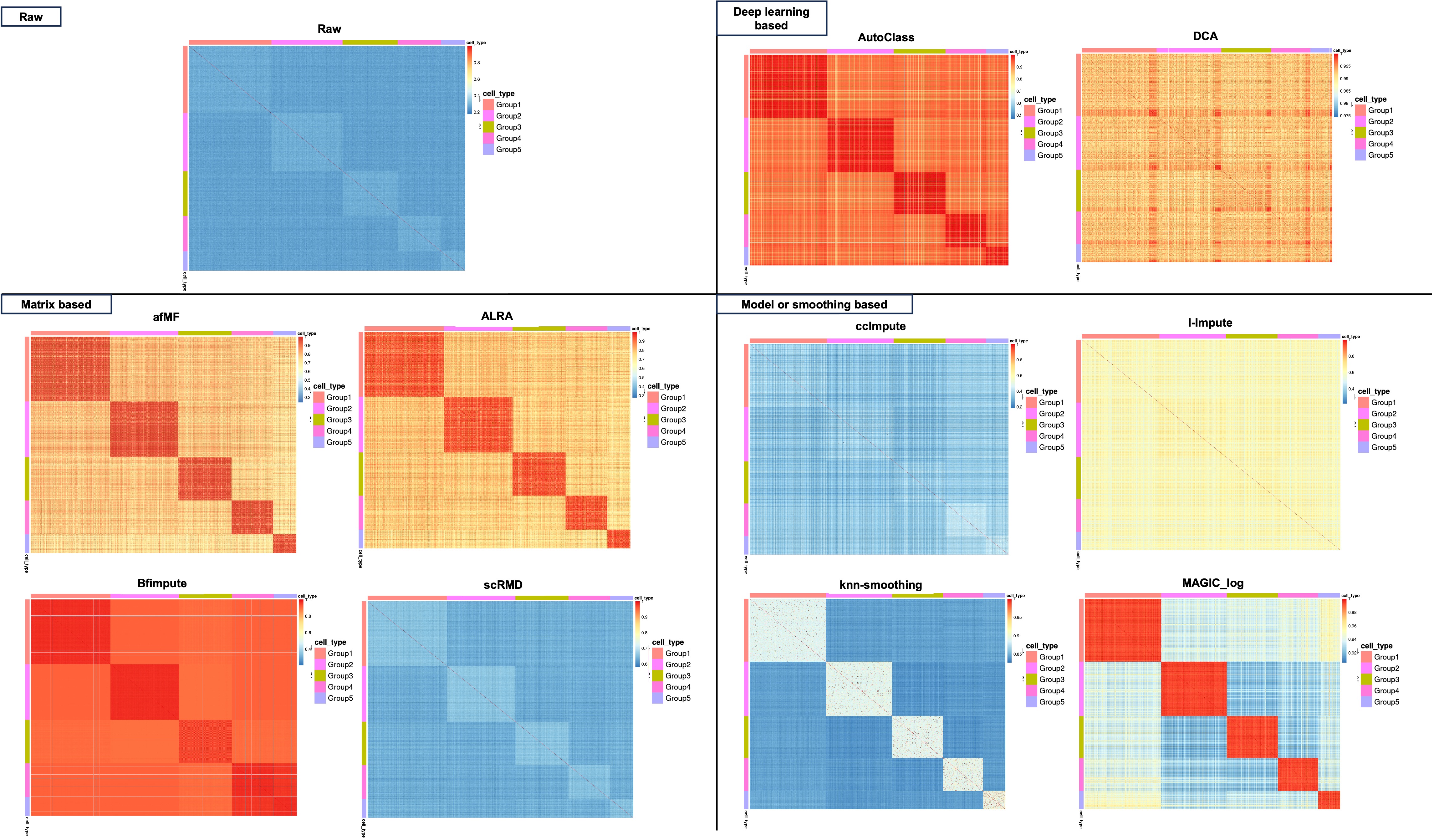
**
