# Supplementary material for: An in‐depth benchmark framework for evaluating single cell RNA‐seq dropout imputation methods and the development of an improved algorithm afMF
Source: Clin Transl Med. 2025 Mar 22;15(4):e70283. doi: 10.1002/ctm2.70283 (PMC11928879; doi:10.1002/ctm2.70283)
Supplement: Supplementary file 10 — Supporting Information [file CTM2-15-e70283-s001.docx]

**Method S10. Running time, memory usage and recommendation.**

*Running time, memory usage and recommendation*

We used four datasets (10000$\times$1500, 10000$\times$5000, 10000$\times$10000 and 10000$\times$50000 matrix) to evaluate the time spent and memory usage of different imputation methods. The time spent and memory usage were plotted in log scale.

A summary heatmap was generated based on the evaluations. The performances were classified into five levels ‘Generally Worse’, ‘Slightly Worse’, ‘No Obvious Difference’, ‘Slightly Better’ and ‘Generally Better’ and were highlighted in different colors. Note that algorithm-evaluation were labelled as ‘No Obvious Difference’ if that algorithm had both obvious advantages and disadvantages based on different metrics in that evaluation. 1-8 cores were used depending on datasets and algorithms, and the maximum memory usage for afMF (i.e., 50,000 cells) is about 22.3 GB.

**Note S10.**

*Running time, memory usage and recommendation*

Good imputation algorithms should have acceptable running time and memory usage. Using four datasets with 1500, 5000, 10000 and 50000 cells, most of the algorithms showed acceptable running time (i.e., within 10 hours), except for I-Impute (**Figure 4A**). Meanwhile, only ccImpute and Bfimpute showed unacceptable memory usage (i.e., >512 GB) on large datasets (**Figure 4B**). Next, performance of different imputation algorithms on different downstream applications & supporting analysis were rated by comparison with no imputation (raw). Better performance was defined if enhancement was seen compared with raw data and it was further divided into two rating: slightly better and generally better. Generally, afMF, ALRA and MAGIC/MAGIC-log had relatively stable performance while others showed less or no improvements or were incompatible with various downstream tools (**Figure 4C-D** and **Table S3**).

We further compared the ALRA and the improved afMF performance in various scRNA-seq analysis. **Table S4** detailly showed the performance of the two algorithms. While performances of some downstream analyses were comparable, afMF gave better results on DE analysis, GSEA, classification and biomarker prediction, and cell clustering.

*Discussion*

Our evaluations revealed that matrix theory-based methods such as afMF and ALRA did not adversely distort the data matrix while handling dropouts. As a class, matrix theory algorithms were demonstrated to have the top performance in most downstream analyses; in contrast, many deep-learning-based algorithms or model-based algorithms were found to overfit the data or generate artefact data structure leading to false positive findings. Of note, choosing a method for conceptual reasons does not necessarily lead to better downstream task results^1^. It is believed that the simpler imputation algorithms such as matrix factorization may be preferred for various scRNA-seq design.

There are several algorithms based on matrix theory to perform imputation. scRMD^2^ targeted to determine the matrices for dropout and noise. Although the concept is good, it needs solve two more additional latent matrices in addition to the underlying latent expression matrix. Bfimpute^3^ is different from most other low-rank MF algorithms in that it subdivided cells into different cell-groups before performing Bayesian based MF. Among the two top algorithms, unlike ALRA, an iterative process is used in afMF to optimize two low-rank matrices which may account for the added benefits shown in these evaluations. While ALRA employs randomized SVD for imputation, afMF utilized full matrix factorization. The iterative process as the key feature of this algorithm allows afMF to refine the matrices and progressively improve the imputation accuracy for the task of matrix completion. Moreover, the algorithms used randomized SVD to approximate the training target which may sacrifice the information enclosed in the original cell-gene information. afMF keeps the original matrix as training target to avoid any information loss with coordinate gradient descent to converge effectively. Meanwhile, afMF keeps the latent dimension selection methods from ALRA with enhanced training method with no information loss.

In addition to the previous established evaluation metrics, we provided more in-depth evaluations incorporating current well-known single cell tools to benchmark imputation methods and explored their compatibilities. While most analyses are compatible with prior imputed data, we revealed one new but important feature in the evaluation of pseudobulk DE analysis. Most imputation methods gave inferior results in limma-trend pseudobulk DE analysis. We reasoned that pseudobulk processing may serve as a smoothing step, and therefore, it may be already less affected by the influence of dropouts. Besides, the compatibility between Pseudotime Trajectory Analysis and prior data imputation depended on the algorithms used. In contrast, imputation was likely to produce false positives in Cell-Cell Communication.

Cell-Cell Communication such as CellPhoneDB used mean values throughout the analysis and should be sensitive to dropout zero counts. The exact reason why it gave inferior results when using imputed data is not clear. We reasoned that imputation may somehow add noise to the mean values of ligands and receptors and thus produced suspicious high level interaction results. Additionally, imputation showed limited or no improvement in 2 out of 3 popular pseudotime trajectory analysis (i.e., Monocle3 and Slingshot), which was also reported by Hou et al.^4^ when they applied different trajectory tools to imputed data. Note that we had used other trajectory tools such as Monocle2 and TSCAN, but they showed worse compatibilities with most imputation methods as well (data not shown). It is suggested that imputation may not be helpful for describing cell-to-cell or gene-to-gene interaction and thus downstream analyses that utilized such information gave inferior results with imputation.

There are correlations between downstream analyses, such as DE analysis and GSEA, biomarker prediction and automatic cell type annotation. And that’s the reason why we see algorithms performed well in one analysis also performed well in another related analysis. For CellPhoneDB, we applied the default routine which had different internal algorithms from what we used in clustering evaluations. Therefore, better clustering results in our assessments may not necessarily result in better CCI analysis. Notably, many factors may affect the true impact of imputation, especially for clustering. There are many clustering algorithms for scRNA-seq data so far, and the various parameters used and the number of PCs selected may also make a difference. What we did is to simplify the problem by using the most common algorithms and the real datasets with simple ground truth label, i.e., cell types. We avoid using datasets with complicated design as they may have multiple ground truth labels for clustering, such as conditions, which obscured the evaluations.

As imputation algorithms are emerging these years, little attention has been put on the compatibility issue. From biologist perspective, it is essential to have an imputation method that is not only to impute the dropout, but also to improve various downstream analyses. Indeed, researchers do not know if they should do prior data imputation in real scRNA-seq analysis so far. Imputation is more widely used in eQTL study^5^ and, ironically, in pseudotime trajectory analysis^6^. According to our evaluations (i.e., DE analysis), imputation is a great option for conducting cell-type-specific eQTL study. Interestingly, imputation was not only applied in scRNA-seq data, but also in qPCR data and genomic data. For instance, genomic variant imputation^7^ has now been widely used in current research. Our study provided a comprehensive illustration of imputation in scRNA-seq, and the methods and framework may be extended to other techniques such as spatial single cell transcriptomics.

The size of the scRNA-seq data is getting larger and larger and thus imputation could take much time and memory usage. Therefore, algorithms with insufficient scalability were not evaluated in this study. According to our results, imputation could be applied on purified cell type or subset data for downstream analysis and thus dramatically reduce time spent and memory usage. Additionally, GPU could be used to accelerate the process as well.

The downstream tools that are designed for analysis of spare data matrix can reduce the effect of the dropout problem in various ways. This raised another question: which is better, imputation in advance vs. algorithms designed for handling sparse data. For example, many model-based algorithms including a zero-inflated model will not be benefited by using prior imputed data. This may somehow explain why imputation does not work well when using some downstream tools as the prior imputation will be redundant. Indeed, many researchers have chosen zero-inflated-model-based tools instead of imputing them in advance, but there are also studies declared that zero-inflated models were not fit for scRNA-seq data^8^. In our point of view, both imputations and models for sparse data are great statistical tools to deal with scRNA-seq data and increase the power to detect biological signals. They can be conducted together as sensitivity analysis to provide stronger evidence. Which one is better has not been evaluated so far. However, researchers should be aware of the compatibilities between imputation and different downstream task software.

It is worth to note that benchmarking various imputation algorithms with various downstream analyses on various datasets is a time-consuming and space-consuming work. Therefore, the data and tools used here are still limited. Our study is served as a comprehensive demonstration of the compatibility between imputation and various popular downstream applications, and it is encouraged that researchers can conduct the imputation benchmark for each downstream application individually^9^ with more datasets, metrics, and tools in the future. Additionally, it would be of great interest to develop downstream applications with internal implementation of imputation algorithms.

Notably, as deep learning based methods are emerging these years, many of the advanced deep learning models such as scGPT^10^ or scFoundation^11^ were developed. However, most of their outputs represent cell embeddings rather than specific imputed gene expression, and thus we did not evaluate them here as many of our evaluations required specific and exact gene information. However, these embedding values are quite useful when performing clustering, pseudotime trajectory analysis, multi-omics integration, or gene module inference that require multiple gene information, etc. We are planning to make in-depth and systematic evaluations specifically for deep-learning models for single cell RNA-seq and these newly developed large models will be our main focus in the next step.

scRNA-seq has been widely used in clinical research and translational medicine. Scientists applied this technology to discover new biomarkers for disease in specific cell types, decipher the cell lineage, disease progression and regulatory activities, and uncover the relationships between various cell types and states in diseases. Due to the sparsity of scRNA-seq data, the power to detect the biological signals may not be enough, and many true positives may be masked by those dropouts. Under this circumstance, imputation provided a way to improve the power to detect those weak true signals, and therefore, promote the new biological discoveries such as disease biomarkers and pathway activities.

**Reference**

1. Ahlmann-Eltze C, Huber W. Comparison of transformations for single-cell RNA-seq data. *Nat Methods*. 2023;20(5):665-672. doi:10.1038/s41592-023-01814-1

2. Chen C, Wu C, Wu L, Wang X, Deng M, Xi R. scRMD: imputation for single cell RNA-seq data via robust matrix decomposition. *Bioinformatics*. 2020;36(10):3156-3161. doi:10.1093/bioinformatics/btaa139

3. Wen ZH, Langsam JL, Zhang L, Shen W, Zhou X. A Bayesian factorization method to recover single-cell RNA sequencing data. *Cell Rep Methods*. 2022;2(1):100133. doi:10.1016/j.crmeth.2021.100133

4. Hou W, Ji Z, Ji H, Hicks SC. A systematic evaluation of single-cell RNA-sequencing imputation methods. *Genome Biol*. 2020;21(1):218. doi:10.1186/s13059-020-02132-x

5. van der Wijst MGP, Brugge H, de Vries DH, et al. Single-cell RNA sequencing identifies celltype-specific cis-eQTLs and co-expression QTLs. *Nat Genet*. 2018;50(4):493-497. doi:10.1038/s41588-018-0089-9

6. SoRelle ED, Dai J, Reinoso-Vizcaino NM, Barry AP, Chan C, Luftig MA. Time-resolved transcriptomes reveal diverse B cell fate trajectories in the early response to Epstein-Barr virus infection. *Cell Rep*. 2022;40(9):111286. doi:10.1016/j.celrep.2022.111286

7. Taliun D, Harris DN, Kessler MD, et al. Sequencing of 53,831 diverse genomes from the NHLBI TOPMed Program. *Nature*. 2021;590(7845):290-299. doi:10.1038/s41586-021-03205-y

8. Kim TH, Zhou X, Chen M. Demystifying “drop-outs” in single-cell UMI data. *Genome Biol*. 2020;21(1):196. doi:10.1186/s13059-020-02096-y

9. Lasri A, Shahrezaei V, Sturrock M. Benchmarking imputation methods for network inference using a novel method of synthetic scRNA-seq data generation. *BMC Bioinformatics*. 2022;23(1):236. doi:10.1186/s12859-022-04778-9

10. Cui H, Wang C, Maan H, et al. scGPT: toward building a foundation model for single-cell multi-omics using generative AI. *Nat Methods*. 2024;21(8):1470-1480. doi:10.1038/s41592-024-02201-0

11. Hao M, Gong J, Zeng X, et al. Large-scale foundation model on single-cell transcriptomics. *Nat Methods*. 2024;21(8):1481-1491. doi:10.1038/s41592-024-02305-7

**Table S3. Summary of benefits and drawbacks of imputations with various applications.**

| **Methods** | **Matrix theory based** | **Model or smoothing based** | **Deep learning based** |
| --- | --- | --- | --- |
| **Differential expression analysis** |  |  |  |
| - Wilcox rank sum | -only afMF enhanced | -no enhancement or inferior | -no enhancement or inferior |
| - MAST | -only afMF enhanced | -MAGIC slightly enhanced | -no enhancement or inferior |
| - Pseudobulk-limma-trend | -no enhancement or inferior | -no enhancement or inferior | -no enhancement or inferior |
| **Gene set enrichment analysis (GSEA)** |  |  |  |
| - -sign log_10_P based | -only afMF enhanced | -only MAGIC enhanced | -no enhancement or inferior |
| - logFC based | -only afMF enhanced | -no enhancement or inferior | -only AutoClass enhanced |
| **Cell type classification and biomarker prediction** | -enhanced | -enhanced | -enhanced |
| **Automatic cell type annotation** |  |  |  |
| - SCINA | -enhanced | -enhanced | -enhanced; except DCA for some cell types |
| - scType | -enhanced | -enhanced | -enhanced; except DCA for some cell types |
| **Cell clustering and cell cycle dynamics** |  |  |  |
| - Louvain | -only afMF enhanced | -MAGIC_log & ccImpute enhanced | -AutoClass enhanced |
| - K-means | -afMF, ALRA & scRMD enhanced | -MAGIC enhanced | -no enhancement or inferior |
| **Dimension reduction (PCA & UMAP)** | -showed consistent patterns | -kNN_smoothing & I_impute  generated artefact in UMAP | -DCA generated artefact in UMAP |
| **pseudotime trajectory analysis** |  |  |  |
| - DPT | -afMF & ALRA enhanced | -all enhanced except I_impute | -no enhancement or inferior |
| - Monocle3 | -no enhancement | -only MAGIC enhanced | -no enhancement |
| - Slingshot | -no enhancement or inferior | -inferior to using raw data | -inferior to using raw data |
| **Advanced analysis: AUCell & SCENIC** | -afMF enhanced | -can generate false positives | -can generate false positives |
| **Advanced analysis: CellPhoneDB and CellChat** | -generated a lot of interactions, likely to be false positives | -generated a lot of interactions, likely to be false positives | -generated a lot of interactions, likely to be false positives |
| **Integration of spatial transcriptomics with scRNA-seq (Seurat)** | -inferior or incompatible | -MAGIC showed consistent patterns | -AutoClass inferior |
| **Supporting analysis** |  |  |  |
| - SC-Bulk profiling similarities | -afMF & ALRA enhanced | -all enhanced | -no enhancement |
| - Surface Protein-mRNA correlation - Distinguish dropouts and real biological zeros | -afMF & ALRA enhanced  -all enhanced | -knn_smoothing enhanced  -enhanced except I-Impute | -all enhanced  -no enhancement |

**Table S4. Performance of the two matrix theory algorithms: ALRA and afMF**

| **Methods** | **ALRA** | **afMF** |
| --- | --- | --- |
| **Dropout elimination for HK genes and cell type marker genes** | Incomplete | Complete |
| **DE analysis** | Only improved in purified datasets | Improved; better than ALRA |
| **GSEA** | Only improved in purified datasets | Improved; better than ALRA |
| **Classification and biomarker prediction** | Improved | Improved; generally better than ALRA |
| **Automatic cell type annotation** | Improved; the extent of improvement over raw data is similar | |
| **Cell Clustering and Dimension reduction** | Improved in K-means algorithm | Improved in both Louvain and K-means algorithms; better than ALRA |
| **Dimension reduction (PCA & UMAP)** | Decent patterns; separate cell types well; similar | |
| **Pseudotime trajectory analysis by DPT** | Improved; the extent of improvement over raw data is similar | |
| **Pseudotime trajectory analysis by Monocle3** | No improvement | |
| **Pseudotime trajectory analysis by Slingshot** | Incompatible; worse than raw log-normalization | |
| **Advanced analysis: AUCell and SCENIC** | Improved; the extent of improvement over raw data is similar | |
| **Advanced analysis: CellPhoneDB and CellChat** | Incompatible; produced false positive interactions | Incompatible; produced even more false positive interactions than ALRA |
| **Integration of spatial transcriptomics with scRNA-seq (Seurat)** | Inferior or incompatible | |
| **SC-Bulk profiling similarities** | Improved | Improved; better than ALRA |
| **Surface Protein-mRNA correlation** | Improved | Improved; worse than ALRA |
| **Distinguish dropouts and real biological zeros** | Improved; better than afMF | Improved |
| **Running time** | Very quick | More running time needed than ALRA but is acceptable (e.g., for 50,000 cells). |
| **Memory usage** | Acceptable | |
